# Syntheses and Structure–Activity Relationships of *N*-Phenethyl-Quinazolin-4-yl-Amines as Potent Inhibitors of Cytochrome *bd* Oxidase in *Mycobacterium tuberculosis*

**DOI:** 10.3390/app11199092

**Published:** 2021-09-29

**Authors:** Sarah M. Hopfner, Bei Shi Lee, Nitin P. Kalia, Marvin J. Miller, Kevin Pethe, Garrett C. Moraski

**Affiliations:** 1 Department of Chemistry and Biochemistry, Montana State University, 103 Chemistry and Biochemistry Building, Bozeman, Montana, MT 59717, USA; 2 School of Biological Sciences, Nanyang Technological University, Singapore 637551, Singapore; 3 Department of Biological Sciences, National Institute of Pharmaceutical Education and Research, Hyderabad, Telangana 500037, India; 4 Department of Chemistry and Biochemistry, University of Notre Dame, 251 Nieuwland Science Hall, Notre Dame, Indiana, IN 46556, USA; 5 Lee Kong Chian School of Medicine, Nanyang Technological University, Experimental Medicine Building, 59 Nanyang Drive, Singapore 636921, Singapore

**Keywords:** tuberculosis, drug development, structure–activity relationships, quinazoline, energy metabolism, cytochrome *bd* oxidase

## Abstract

The development of cytochrome *bd* oxidase (cyt-*bd*) inhibitors are needed for comprehensive termination of energy production in *Mycobacterium tuberculosis* (Mtb) to treat tuberculosis infections. Herein, we report on the structure-activity-relationships (SAR) of 22 new *N*-phenethyl-quinazolin-4-yl-amines that target cyt-*bd*. Our focused set of compounds was synthesized and screened against three mycobacterial strains: *Mycobacterium bovis* BCG, *Mycobacterium tuberculosis* H37Rv and the clinical isolate *Mycobacterium tuberculosis* N0145 with and without the cytochrome *bcc:aa*_*3*_ inhibitor Q203 in an ATP depletion assay. Two compounds, **12a** and **19a**, were more active against all three strains than the naturally derived cyt-*bd* inhibitor aurachin D.

## 1. Introduction

Tuberculosis (TB) is still a leading cause of death from an infectious agent. According to the World Health Organization’s 2020 Global Tuberculosis Report, over 10 million people became ill with the disease in 2019 [1]. Action must be taken to combat this continuing public health threat. TB is caused by the bacterium *Mycobacterium tuberculosis* (Mtb) and spread by aerosols of an infected person through a cough or sneeze. Treatment of individuals infected with TB consists of several medications that are taken together for 6–9 months. First-line drugs include isoniazid (INH), rifampin (RIF), ethambutol (EMB) and pyrazinamide (PZA) [1]. Other barriers, such as lengthy treatment regimens and access to care, have made adherence to treatment challenging. Shorter treatment schedules are strongly desired. Despite the advances in drug therapies and modern medications, such as bedaquiline, delamanid and pretomanid, there is a need for comprehensive drug combinations and new treatment regimens to combat drug resistant TB [2]. Consideration of how Mtb survives onslaughts of chemotherapies gives insight into more effective treatment. Investigating the mechanism of action of antibiotics provides critical information that can be used to ultimately cure TB infections [3]. Targets within energy metabolism processes, specifically those involved in the oxidative phosphorylation pathway, have become of increased interest. Mtb can persist in a hypoxic, non-replicating, drug-tolerant state [4]. It has been demonstrated that interrupting oxidative phosphorylation in Mtb is an encouraging strategy [5–7]. While compounds have been made to inhibit various targets within the oxidative phosphorylation pathway, limitations have been observed, including the inability to inhibit oxygen respiration and clear Mtb infection due to a redundancy between the terminal oxidases cytochrome *bcc:aa*_*3*_ (cyt-*bcc:aa*_*3*_) and cytochrome *bd* (cyt-*bd*) [8]. Cytochrome *bcc:aa*_*3*_ is the main aerobic terminal oxidase in Mtb [9]. The compound Q203 (**4,** Telacebec®) inhibits cytochrome *bcc* by binding to the QcrB subunit, thereby inhibiting the function of the cytochrome *bcc:aa*_*3*_ super-complex [10]. When cytochrome *bcc:aa*_*3*_ is inactive, cyt-*bd* is upregulated [11]. This adaptation provides protection against cytochrome *bcc:aa*_*3*_ inhibitors such as Q203. We have shown that targeting cyt-*bd* in combination with Q203 is bactericidal against replicating and non-replicating Mtb [12]. This makes cyt-*bd* an enticing target for the development of antituberculosis agents to effectively eliminate infection.

**Figure 1.**
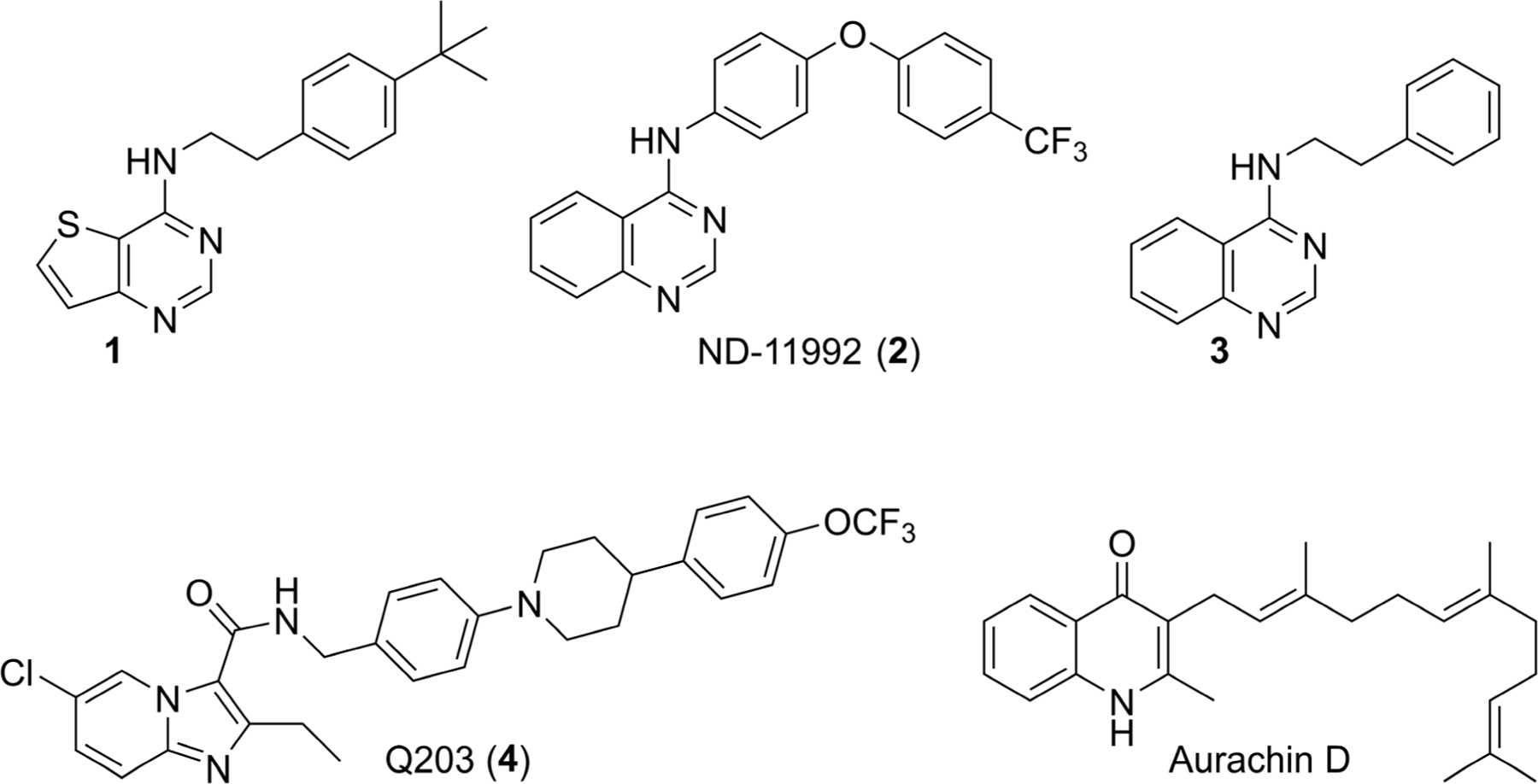
Known synthetically accessible small molecule cyt-*bd* inhibitors: thieno[3,2-*d*]pyrimidin-4-amine (**1**) [13], ND-11992 (**2**) [12], and *N*-phenethylquinazolin-4-amine (**3**) screening hit. The selective cyt-*bcc:aa*_*3*_ inhibitor Q203 (**4**) [10] and natural cyt-*bd* inhibitor aurachin D [7].

Interested in the inhibition of this target, we screened a small set (56) of antibacterial compounds that originated from our decades-old antibacterial program. This led to the identification of cyt-*bd* compounds like the thieno[3,2-*d*]pyrimidin-4-amines in which (**1**) is an example, the in vivo active optimized inhibitor ND-11992 (**2**) and hit compound **3** (Figure 1) [13].

The *N*-phenethylquinazolin-4-amine class, exemplified by compound **3** (Figure 1), has an IC_50_ of 11 µM against *Mycobacterium bovis* BCG and an IC_50_ of 27 µM against *Mycobacterium tuberculosis* H37Rv as determined by our published ATP depletion assay [13]. This assay is purposefully run in the presence and absence of Q203 (**4**, a selective cyt-*bcc:aa*_*3*_ inhibitor) to indicate whether a compound inhibits alone or synergizes due to the conditional essentiality of cyt-*bd* to maintain ATP homeostasis once cyt-*bcc:aa*_*3*_ is selectively inhibited by Q203. Compound **3** was inactive (IC_50_ > 25 µM) in the absence of Q203 which is an indication of on-target potency based upon assay design. Similarly, compound **3** was amenable to chemical modifications and structure–activity relationship (SAR) development. Herein, we report our initial design, synthesis, and activity assessment of various quinazoline compounds for activity against cyt-*bd* of *Mycobacterium bovis* BCG and *Mycobacterium tuberculosis*.

## 2. Materials and Methods

### 2.1 Chemistry

#### General.

All anhydrous solvents, reagent grade solvents for chromatography and starting materials were purchased from either Aldrich Chemical Co. (Milwaukee, WI, USA) or Fisher Scientific (Suwanee, GA, USA) unless otherwise noted. Water was distilled and purified through a Milli-Q water system (Millipore Corp., Bedford, MA, USA). General methods of purification of compounds involved the use of silica cartridges purchased from Practichem, LLC. (www.practichem.com, last accessed on 23 September 2021) and/or recrystallization. The reactions were monitored by TLC on precoated Merck 60 F254 silica gel plates and visualized using UV light (254 nm). All compounds were analyzed for purity by HPLC and characterized by ^1^H and ^13^C NMR using a Bruker Ascend Avance III HD Spectrometer (500 MHz). Chemical shifts are reported in ppm (δ) relative to the residual solvent peak in the corresponding spectra; chloroform δ 7.27 and δ 77.23, methanol δ 3.31 and δ 49.00 and coupling constants (*J*) are reported in hertz (Hz) (where, s = singlet, bs = broad singlet, d = doublet, dd = double doublet, bd = broad doublet, ddd = double doublet of doublet, t = triplet, tt = triple triplet, q = quartet, m = multiplet) and analyzed using MestreNova NMR data processing. ^19^F NMR were run without a standard and are uncorrected. Mass spectra values are reported as m/z. Melting points were measured on a Buchi B-545 melting point instrument and are uncorrected, measured against benzoic acid 118.8–120.2 °C. Compounds **3**, **6a**–**26a** appear within US Provisional Patent Application No. 62/783,984. The quinazolines (**5**, **16**–**26**) and amines (**6**–**15**) used in synthesis are all commercially available.

#### LC-MS method:

The liquid chromatography–mass spectrometry method was performed on an Agilent 1290 infinity coupled to Agilent 6538 Ultra High-Definition Quadrupole Time of Flight (UHD-QTOF) instrument. A separation was achieved by using reverse phase Waters Acuity UPLC HSS T3 1.8 µm (2.1 × 100mm) column from Waters (Milford, MA, USA). All solvents were purchased from Fischer Scientific LCMS Optima grade solvents. Water containing 0.1% formic acid was used as mobile phase A and acetonitrile containing 0.1% formic acid was used as mobile phase B. The injection volume was set at 1 µL. Samples were injected in a gradient of 95% mobile phase A and 5% mobile phase B in the initial condition to 5% mobile phase A and 95% mobile phase B in 9 min. The eluent was held at that composition for an additional 3 min and switched back to the initial condition at 12 min.

The MS data acquisition was performed from 50–1000 m/z at 1.0 spectra/sec scan rate. The source gas temperature was set at 350 °C with a flow of 8 l/min. The nebulizer gas was set at 55 psig. The capillary voltage was set at 3500 volts with fragmentor at 100, skimmer at 45 and octopole RF 500 volts. Prior to sample runs, the instrument was calibrated using Agilent low mass calibrant solution.

#### Data analysis:

The data collected in Agilent LC-MS was analyzed using Agilent Mass Hunter software for HRMS calculation.

#### General Syntheses of N-Phenethylquinazolin-4-Amine Analogs

General procedure A for base catalyzed S_N_Ar reaction for the preparation of compounds **6a**, **7a**, **9a**, **12a**-**26a**. In a sealed vial, 4-chloroquinazoline (**5**, 100 mg, 0.61 mmol, 1 equiv.), 2-[4-(trifluoromethoxy)phenyl]ethylamine (**7**, 125 mg, 0.61 mmol, 1 equiv.) and potassium carbonate (84 mg, 0.61 mmol, 1 equiv. were combined in DMSO (4 mL). The reaction was heated to 100 °C for 12 h. The reaction mixture was concentrated to dryness. The residue was extracted with CH_2_Cl_2_ and washed with 5% aqueous acetic acid solution (2x), water and brine. The organic phase was collected, dried over sodium sulfate, filtered, and concentrated in vacuo. Crude material obtained was purified by either silica gel chromatography with a gradient of CH_2_Cl_2_: ethyl acetate: solvent system (0–80%) or recrystallized from hot isopropanol or acetonitrile to afford the product.

General procedure B for acid catalyzed S_N_Ar reaction for the preparation of compounds **3**, **8a**, **10a**, **11a**. In a sealed vial, desired 4-chloroquinazoline **16**–**26** (1 equiv.) and 2-[4-(trifluoromethoxy)phenyl]ethylamine (**7**, 1 equiv.) were dissolved in a 3:1 tetrahydrofuran: 2-propanol solution (4 mL). Next, was added 12 M HCl (~0.4 equiv.). The solution was heated at 70 °C for 24 h. The reaction was concentrated to dryness. The residue was dissolved in CH_2_Cl_2_ and washed with saturated aqueous NaHCO_3_ solution, water, and brine. The organic phase was collected, dried over sodium sulfate, filtered, and concentrated in vacuo. Crude material obtained was purified by either silica gel column chromatography with a gradient of CH_2_Cl_2_: ethyl acetate: solvent system (0–80%) or recrystallized from hot isopropanol or acetonitrile to afford the product.

#### *N*-Phenethylquinazolin-4-amine, 3

Following general procedure B, using 4-chloroquinazoline (**5**, 100 mg, 0.59 mmol), 2-phenylethylamine (72 mg, 0.59 mmol), and 12 N HCl (11 µL, 0.14 mmol) gave **3** as pale-yellow crystals (43 mg, 28%). mp 171.5–171.9 °C; ^1^H (500 MHz, MeOD) δ ppm 8.47 (s, 1H), 8.06 (d, *J =* 8.3 Hz, 1H), 7.82–7.77 (m, 1H), 7.72 (d, *J =* 8.3 Hz, 1H), 7.55–7.50 (m, 1H), 7.31–7.26 (m, 4H), 7.23–7.17 (m, 1H), 3.86 (t, *J =* 7.4 Hz, 2H), 3.04 (t, *J =* 7.4 Hz, 2H) ^13^C (125 MHz, MeOD) δ ppm 160.0, 154.7, 148.2, 139.3, 132.7, 128.5, 128.1, 126.3, 126.0, 125.9, 121.9, 115.1, 42.5, 34.7. HRMS (EI), M + 1 calculated for C_16_H_15_N_3_, 250.1339, found 250.1351. These experimental results are consistent with previous reports [14–16].

#### Quinazolin-4-yl{2-[4-(trifluoromethyl)phenyl]ethyl}amine, 6a

Following general procedure A, using 4-chloroquinazoline (**5**, 100 mg, 0.59 mmol), 2-[4-(trifluoromethyl)phenyl]ethylamine (114 mg, 0.59 mmol) and potassium carbonate (81 mg, 0.59 mmol) gave **6a** as gold crystals (79 mg, 38%). mp 160.3–161.0 °C; ^1^H (500 MHz, CDCl_3_) δ ppm 8.73 (s, 1H), 7.88 (d, *J =* 7.9 Hz, 1H), 7.77 (ddd, *J =* 8.4, 7.0, 1.3 Hz), 7.63–7.57 (m, 3H), 7.47 (ddd, *J =* 8.2, 7.0, 1.2 Hz), 7.40 (d, *J =* 8.0 Hz, 2H), 5.79 (br.s, 1H), 3.99 (dt, *J* = 6.9, 5.9 Hz, 2H), 3.14 (t, *J =* 6.9 Hz, 2H). ^13^C (125 MHz, CDCl_3_) δ ppm 159.6, 155.4, 149.5, 143.2 (d, *J* = 1.5 Hz), 132.7, 129.2, 129.1 (q, *J* = 32.4 Hz), 128.2, 126.1, 125.6 (q, *J* = 3.8 Hz), 124.7 (q, *J* = 271.9 Hz), 120.2, 114.9, 42.1, 35.2. ^19^F (470 MHz, CDCl_3_) δ ppm −62.4 (s, 3F). HRMS (EI), M + 1 calculated for C_17_H_14_F_3_N_3_, 318.1213, found 318.1229. This compound appears in referenced patent and fungicidal activity was reported [17].

#### Quinazolin-4-yl{2-[4-(trifluoromethoxy)phenyl]ethyl}amine, 7a

Following general procedure A, using 4-chloroquinazoline (**5**, 100 mg, 0.59 mmol), 2-[4-(trifluoromethoxy)phenyl]ethylamine (125 mg, 0.59 mmol) and potassium carbonate (84 mg, 0.59 mmol) gave **7a** as white crystals (85 mg, 42%). mp 137.4–137.8 °C; ^1^H (500 MHz, CDCl_3_) δ ppm 8.73 (s, 1H), 7.88 (d, *J* = 8.2 Hz, 1H), 7.77 (ddd, *J* = 8.4, 7.0, 1.3 Hz, 1H), 7.60–7.56 (m, 1H), 7.48 (ddd, *J* = 8.3, 7.0, 1.1 Hz), 7.33–7.26 (m, 2H), 7.20 (d, *J* = 8.0 Hz, 2H), 5.73 (br.s, 1H), 3.97 (dt, *J* = 7.0, 5.9 Hz, 2H), 3.08 (t, *J* = 7.0 Hz, 2H). ^13^C (125 MHz, CDCl_3_) δ ppm 159.3, 155.4, 149.5, 148.0 (d, *J* = 1.8 Hz), 137.7, 132.6, 130.2, 128.8, 126.1, 121.3, 120.6 (q, *J* = 235.0 Hz), 120.1, 114.9, 42.3, 34.6. ^19^F (470 MHz, CDCl_3_) δ ppm −59.9 (s, 3F). HRMS (EI), M + 1 calculated for C_17_H_14_F_3_N_3_O, 334.1162, found 334.1155.

#### [2-(4-Chlorophenyl)ethyl]quinazolin-4-ylamine, 8a

Following general procedure B, using 4-chloroquinazoline (**5**, 100 mg, 0.59 mmol), 2-(4-chlorophenyl)ethylamine (94 mg, 0.59 mmol) and 12 N HCl (11 µL, 0.14 mmol) gave **8a** as yellow crystals (35 mg, 20%). mp 191.0–192.1 °C; ^1^H (500 MHz, MeOD) δ ppm 8.47 (s, 1H), 8.05 (dd, *J =* 8.3, 0.7 Hz, 1H), 7.80 (ddd, *J =* 8.3, 7.0, 1.3 Hz, 1H), 7.73–7.70 (m, 1H), 7.53 (ddd, *J =* 8.3, 7.0, 1.3 Hz, 1H), 7.30–7.25 (m, 2H), 3.88–3.83 (m, 2H), 3.03 (t, *J =* 7.3 Hz, 2H). ^13^C (125 MHz, MeOD) δ ppm 160.0, 154.6, 148.2, 138.1, 132.8, 131.8, 130.2, 128.1, 126.3, 126.1, 121.9, 115.1, 42.2, 34.0. HRMS (EI), M + 1 calculated for C_16_H_14_ClN_3_, 284.0949, found 284.0950. These experimental results are consistent with previous reports [18–20].

#### [2-(4-(Pentafluoro-(λ)^6^-sulfaneyl]quinazolin-4-ylamine, 9a

Following general procedure A, using 4-chloroquinazoline (**5**, 70 mg, 0.42 mmol) and 2-(4-(pentafluoro-(λ)^6^-sulfaneyl)phenyl)ethan-1-amine (105 mg, 0.42 mmol) and potassium carbonate (59 mg, 0.42 mmol) gave **9a** as white crystals (97 mg, 61%). mp 169.2–170.7 °C; ^1^H (500 MHz, CDCl_3_) δ ppm 8.73 (s, 1H), 7.89 (d, *J =* 8.3 Hz, 1H), 7.80–7.70 (m, 3H), 7.61 (d, *J =* 8.2 Hz, 1H), 7.51–7.46 (m, 1H), 7.37 (d, *J =* 8.3 Hz, 2H), 5.80 (br.s, 1H), 3.99 (q, *J* = 6.6 Hz, 2H), 3.14 (t, *J =* 6.9 Hz, 2H). ^13^C (125 MHz, CDCl_3_) δ ppm 159.3, 155.3, 152.5 (quintet, *J* = 17.6 Hz), 149.5, 143.2, 132.7, 129.1, 128.8, 126.3 (quintet, *J* = 4.5 Hz), 120.2, 114.9, 42.1, 34.9. ^19^F (470 MHz, CDCl_3_) δ ppm 84.8 (pent, *J* = 150.0 Hz, 1F), 63.1 (d, *J* = 150.0 Hz, 4F). HRMS (EI), M + 1 calculated for C_16_H_14_F_5_N_3_S, 376.0901, found 376.0908.

#### [2-(4-Methylpheny)ethyl]quinazolin-4-ylamine, 10a

Following general procedure B, using 4-chloroquinazoline (**5**, 100 mg, 0.59 mmol), 2-(4-methylphenyl)ethan-1-amine (82 mg, 0.59 mmol) and 12 N HCl (11 µL, 0.14 mmol) gave **10a** as white crystals (25 mg, 15%). mp 183.6–184.6 °C; ^1^H (500 MHz, CDCl_3_) δ ppm 8.71 (s, 1H), 7.86 (d, *J =* 8.2 Hz, 1H), 7.74 (ddd, *J =* 8.3, 7.0, 1.3 Hz, 1H), 7.56 (d, *J =* 7.8 Hz, 1H), 7.44 (ddd, *J =* 8.2, 7.0, 1.1 Hz, 1H), 5.79 (br.s, 1H), 3.95 (dt, *J =* 6.7, 5.8 Hz, 2H), 3.02 (t, *J* = 6.8 Hz, 2H), 2.37 (s, 3H). ^13^C (125 MHz, CDCl_3_) δ ppm 159.3, 155.4, 149.3, 136.3, 135.7, 132.6, 129.5, 128.7, 128.6, 126.0, 120.3, 115.0, 42.3, 34.8, 21.1. HRMS (EI), M + 1 calculated for C_17_H_17_N_3_, 264.1495, found 264.1496.

#### [2-(4-Methoxyphenyl)ethyl]quinazolin-4-ylamine, 11a

Following general procedure B, using 4-chloroquinazoline (**5**, 100 mg, 0.59 mmol), 2-(4-methoxyphenyl)ethylamine (91 mg, 0.59 mmol) and 12 N HCl (11 µL, 0.14 mmol) gave **11a** as off-white crystals (74 mg, 43%). mp 172.3–172.9 °C; 1H (500 MHz, CDCl_3_) δ ppm 8.71 (s, 1H), 7.86 (d, *J =* 8.3 Hz, 1H), 7.74 (ddd, *J =* 8.3, 7.0, 1.3 Hz, 1H), 7.56 (d, *J =* 8.3 Hz, 1H), 7.45 (ddd, *J =* 8.2, 7.0, 1.2 Hz, 1H), 7.20 (dt, *J =* 8.6, 2.9 Hz, 2H), 6.90 (dt, *J =* 8.6, 2.9 Hz, 2H), 5.78 (s, 1H), 3.93 (dt, *J =* 6.8, 5.7 Hz, 2H), 3.83 (s, 3H), 3.01 (t, *J =* 6.8 Hz, 2H). ^13^C (125 MHz, CDCl_3_) δ ppm 159.3, 158.4, 155.4, 149.4, 132.6, 130.8, 129.8, 128.6, 126.0, 120.3, 115.0, 114.2, 55.3, 42.4, 34.3. HRMS (EI), M + 1 calculated for C_17_H_17_N_3_O, 280.1444, found 280.1458.

#### {2-[4-(*tert*-Butyl)phenyl]ethyl}quinazolin-4-ylamine, 12a

Following general procedure A, using 4-chloroquinazoline (**5**, 100 mg, 0.59 mmol), 2-[4-(tert-butyl)phenyl]ethylamine (110 mg, 0.59 mmol) and potassium carbonate (81 mg, 0.59 mmol) gave **12a** as yellow crystals (58 mg, 29%). mp 149.5–151.3 °C; ^1^H (500 MHz, CDCl_3_) δ ppm 8.71 (s, 1H), 7.86 (dd, *J =* 8.4, 0.6 Hz, 1H), 7.74 (ddd, *J =* 8.4, 7.0, 1.3 Hz, 1H), 7.57 (dd, *J =* 8.3, 0.8 Hz, 1H), 7.45 (ddd, *J =* 8.2, 7.0, 1.2 Hz, 1H), 7.39 (dt, *J =* 8.3, 2.3 Hz, 2H), 7.22 (dt, *J =* 8.3, 2.2 Hz, 2H), 5.81 (br.s, 1H), 3.96 (dt, *J =* 6.8, 5.7 Hz, 2H), 3.04 (t, *J* = 6.8 Hz, 2H), 1.35 (s, 3H). ^13^C (125 MHz, CDCl_3_) δ ppm 159.3, 155.5, 149.6, 149.5, 135.8, 132.5, 128.7, 128.5, 125.9, 125.7, 120.3, 115.0, 42.3, 34.7, 34.5, 31.4. HRMS (EI), M + 1 calculated for C_20_H_23_N_3_, 306.1965, found 306.1967. These experimental results are consistent with a previous report [21].

#### Quinazolin-4-yl{2-[3-(trifluoromethoxy)phenyl]ethyl}amine, 13a

Following general procedure A, using 4-chloroquinazoline (**5**, 100 mg, 0.59 mmol), 3-(trifluoromethoxy)phenylamine (125 mg, 0.59 mmol) and potassium carbonate (84 mg, 0.59 mmol) gave **13a** as white crystals (136 mg, 67%). mp 144.0–144.9 °C; ^1^H (500 MHz, CDCl_3_) δ ppm 8.72 (s, 1H), 7.87 (d, *J =* 7.8 Hz), 7.76 (ddd, *J =* 8.4, 7.0, 1.3 Hz, 1H), 7.60 (ddd, *J =* 8.2, 7.0, 1.2 Hz, 1H), 7.39–7.35 (m, 1H), 7.22–7.19 (m, 1H), 7.16–7.12 (m, 1H), 5.83 (br.s, 1H), 3.97 (dt, *J =* 6.9, 5.9 Hz, 2H), 3.09 (t, *J =* 6.9 Hz, 2H). ^13^C (125 MHz, CDCl_3_) δ ppm 159.3, 155.4, 149.5 (q, *J* = 1.8 Hz), 141.3, 132.6, 130.1, 128.7, 127.3, 126.1, 121.4, 120.5 (q, *J* = 257.2 Hz), 120.2, 119.1, 114.9, 42.1, 35.1. ^19^F (470 MHz, CDCl_3_) δ ppm −57.7 (s, 3F). HRMS (EI), M + 1 calculated for C_17_H_14_F_3_N_3_O, 306.1965, found 306.1967.

#### [2-(3-Ethoxyphenyl)ethyl]quinazolin-4-ylamine, 14a

Following general procedure A, using 4-chloroquinazoline (**5**, 151 mg, 0.92 mmol), 3-ethoxyphenylethylamine (155 mg, 0.92 mmol) and potassium carbonate (127 mg, 0.92 mmol) gave **14a** as off-white crystals (211 mg, 78%). mp 148.5–148.8 °C; ^1^H (500 MHz, CDCl_3_) δ ppm 8.71 (s, 1H), 7.86 (d, *J =* 8.3 Hz, 1H), 7.74 (ddd, *J =* 8.3, 7.0, 1.2 Hz, 1H), 7.56 (d, *J* = 8.0 Hz, 1H), 7.44 (ddd, *J =* 8.0, 7.0, 1.1 Hz, 1H), 6.86 (d, *J =* 7.5 Hz, 1H), 6.83–6.79 (m, 2H), 5.79 (br.s, 1H), 4.02 (q, *J =* 7.0 Hz, 2H), 3.96 (dt, *J =* 6.7, 5.8 Hz, 2H), 1.42 (t, *J =* 7.0 Hz, 3H). ^13^C (125 MHz, CDCl_3_) δ ppm 159.4, 159.3, 155.5, 149.5, 140.4, 132.5, 129.8, 128.7, 126.0, 121.0, 120.3, 115.1, 115.0, 112.6, 63.4, 42.1, 35.3, 14.8. HRMS (EI), M + 1 calculated for C_18_H_19_N_3_O, 294.1601, found 294.1616.

#### Quinazolin-4-yl{2-[2-(trifluromethyl)phenyl]ethyl}amine, 15a

Following general procedure A outlined, using 4-chloroquinazoline (**5**, 100 mg, 0.59 mmol), 2-(2-trifluoromethylphenyl)ethylamine (117 mg, 0.59 mmol) and potassium carbonate (81 mg, 0.59 mmol) gave **15a** as a yellow flaky solid (68 mg, 34%). mp 134.4–137.4 °C; ^1^H (500 MHz, CDCl_3_) δ ppm 8.66 (s, 1H), 8.12–8.05 (m, 1H), 7.95 (d, *J =* 8.3 Hz, 1H), 7.80–7.74 (m, 1H), 7.66 (d, *J =* 7.8 Hz, 1H), 7.54–7.49 (m, 1H), 7.49–7.46 (m, 1H), 7.37–7.32 (m, 1H), 4.04 (dt, *J =* 6.9, 6.7 Hz, 2H), 3.30 (t, *J =* 7.2 Hz, 2H). ^13^C (125 MHz, CDCl_3_) δ ppm 159.9, 153.5, 145.7, 137.3, 133.6, 132.0, 131.7, 128.9 (q, *J* = 29.7 Hz), 126.9, 126.1 (q, *J* = 5.7 Hz), 126.8, 125.7, 124.5 (q, *J* = 254.0 Hz), 121.9, 114.4, 42.7, 31.8. ^19^F (470 MHz, CDCl_3_) δ ppm 59.2 (s, 3F). HRMS (EI), M + 1 calculated for C_17_H_14_F_3_N_3_, 318.1213, found 318.1220. This compound appears in referenced patent and fungicidal activity was reported [17].

#### (6-Fluoroquinazolin-4-yl){2-[4-(trifluormethoxy)phenyl]ethyl}amine, 16a

Following general procedure A, using 4-chloro-6-fluoroquinazoline (**16**, 100 mg, mmol), 2-[4-(trifluromethoxy)phenyl]ethylamine (112 mg, 0.55 mmol), and potassium carbonate (76 mg, 0.55 mmol) gave **16a** as a white solid (99 mg, 51%). mp 171.6–172.6 °C; ^1^H (500 MHz, CDCl_3_) δ ppm 8.70 (s, 1H), 7.89 (dd, *J =* 9.1, 5.3 Hz, 1H), 7.52 (ddd, 9.1, 8.2, 2.7 Hz, 1H), 7.32–7.29 (m, 2H), 7.23–7.19 (m, 3H), 5.57 (br.s, 1H), 3.96 (dt, *J =* 7.0, 5.8 Hz, 2H), 3.08 (t, *J =* 7.0 Hz, 2H). ^13^C (125 MHz, CDCl_3_) δ ppm 160.0 (d, *J* = 248.9 Hz), 159.0, 148.1 (q, *J* = 1.9 Hz), 146.5, 137.5, 131.4 (d, *J* = 8.4 Hz), 130.1, 122.2 (d, *J* = 24.4 Hz), 121.5, 121.3, 120.5 (q, *J* = 255.5 Hz), 115.2 (d, *J* = 7.9 Hz), 104.7 (d, *J* = 22.7 Hz), 42.4, 34.6. ^19^F (470 MHz, CDCl_3_) δ ppm −57.9 (s, 3F), −112.3 (ddd, *J* = 5.4, 8.5, 8.5 Hz, 1F). HRMS (EI), M + 1 calculated for C_17_H_13_F_4_N_3_O, 352.1068, found 352.1078. This compound was shown to inhibit autophagy in referenced patent [22].

#### (6-Methylquinazolin-4-yl){2-[4-(trifluormethoxy)phenyl]ethyl}amine, 17a

Following general procedure A, using 4-chloro-6-methylquinazoline (**17**, 100 mg, mmol), 2-[4-(trifluoromethoxy)phenyl]ethylamine (115 mg, 0.56 mmol), and potassium carbonate (77 mg, 0.56 mmol) gave **17a** as an off-white solid (134 mg, 69%). mp 134.3–135.0 °C; ^1^H (500 MHz, CDCl_3_) δ ppm 8.67 (s, 1H), 7.77 (d, *J* = 8.5 Hz, 1H), 7.58 (dd, *J* = 8.5, 1.6 Hz, 1H), 7.36 (s, 1H), 7.31–7.27 (m, 2H), 5.76 (br.s, 1H), 3.94 (dt, *J =* 6.9, 5.9 Hz, 2H), 3.08 (t, *J =* 7.0 Hz, 2H), 2.50 (s, 3H). ^13^C (125 MHz, CDCl_3_) δ ppm 158.9, 154.6, 148.0 (q, *J* = 1.8 Hz), 147.8, 137.8, 136.1, 134.5, 130.2, 128.5, 121.2, 120.5 (q, *J* = 256.9 Hz), 119.4, 114.8, 42.3, 34.7, 21.7. ^19^F (470 MHz, CDCl_3_) δ ppm −57.9 (s, 3F). HRMS (EI), M + 1 calculated for C_18_H_16_F_3_N_3_O, 348.1318, found 348.1336.

#### (6-Methoxyquinazolin-4-yl){2-[4-(trifluormethoxy)phenyl]ethyl}amine, 18a

Following general procedure A, using 4-chloro-6-methoxyquinazoline (**18**, 100 mg, 0.51 mmol), 2-[4-(trifluoromethoxy)phenyl]ethylamine (105 mg, 0.51 mmol), and potassium carbonate (71 mg, 0.51 mmol) gave **18a** as a white solid (93 mg, 50%). mp 144.7–145.2 °C; ^1^H (500 MHz, CDCl_3_) δ ppm 8.64 (s, 1H), 7.81 (d, *J =* 9.1 Hz, 1H), 7.41 (dd, *J =* 9.1, 2.5 Hz, 1 Hz), 7.32–7.26 (m, 2H), 7.20 (d, *J =* 8.1 Hz, 2H), 6.82 (d, *J =* 2.5 Hz, 1H), 5.65 (br.s, 1H), 3.95 (dt, *J =* 6.8, 6.2 Hz, 2H), 3.87 (s, 3H), 3.08 (t, *J =* 7.0 Hz, 2H). ^13^C (125 MHz, CDCl_3_) δ ppm 158.6, 157.6, 153.4, 148.0 (q, *J* = 1.8 Hz), 144.9, 137.9, 130.3, 130.2, 123.6, 121.2, 120.5 (q, *J* = 258.4 Hz), 115.3, 99.8, 55.6, 42.3, 34.7. ^19^F (470 MHz, CDCl_3_) δ ppm −57.9 (s, 3F). HRMS (EI), M + 1 calculated for C_18_H_16_F_3_N_3_O_2_, 364.1267, found 364.1276.

#### (7-Fluoroquinazolin-4-yl){2-[4-(trifluormethoxy)phenyl]ethyl}amine, 19a

Following general procedure A, using 4-chloro-7-fluoroquinazoline (**19**, 100 mg, 0.55 mmol), 2-[4-(trifluoromethoxy)phenyl]ethylamine (112 mg, 0.55 mmol), and potassium carbonate (76 mg, 0.55 mol) gave **19a** as off-white crystals (107 mg, 56%). mp 128.9–129.7 °C; ^1^H (500 MHz, CDCl_3_) δ ppm 8.69 (s, 1H), 7.60 (dd, *J =* 9.1, 5.6 Hz, 1H), 7.49 (dd, *J* = 9.8, 2.5 Hz, 1H), 7.32–7.26 (m, 2H), 7.24–7.17 (m, 3H), 5.74 (br.s, 1H), 3.96 (dt, *J =* 6.9, 5.9 Hz, 2H), 3.07 (t, *J =* 7.0 Hz, 2H). ^13^C (125 MHz, CDCl_3_) δ ppm 165.0 (d, *J* = 253.3 Hz), 159.1, 156.4, 151.6 (d, *J* = 13.1 Hz), 148.1 (q, *J* = 2.0 Hz), 137.6, 130.1, 122.8 (d, *J* = 10.4 Hz), 121.3, 120.5 (q, *J* = 256.9 Hz), 115.8 (d, *J* = 24.9 Hz), 112.9 (d, *J* = 20.4 Hz), 111.8, 42.3, 34.6. ^19^F (470 MHz, CDCl_3_) δ ppm −57.9 (s, 3F), −105.3 (ddd, *J* = 5.3, 9.2, 9.2 Hz, 1F). HRMS (EI), M + 1 calculated for C_17_H_13_F_4_N_3_O, 352.1068, found 352.1072.

#### (7-Chloroquinazolin-4-yl){2-[4-(trifluormethoxy)phenyl]ethyl}amine, 20a

Following general procedure A, using 4,7-dichloroquinazoline (**20**, 100 mg, 0.51 mmol), 2-[4-(trifluoromethoxy)phenyl]ethylamine (105 mg, 0.51 mmol), and potassium carbonate (78 mg, 0.56 mol) gave **20a** as off-white flaky crystals (72 mg, 50%). mp 153.0–153.4 °C; ^1^H (500 MHz, MeOD) δ ppm 8.47 (s, 1H), 8.03 (d, *J* = 8.6 Hz, 1H), 7.69 (d, *J* = 2.1 Hz, 1H), 7.49 (dd, *J* = 8.6, 2.1 Hz, 1H), 7.36 (dt, *J* = 8.6, 2.7 Hz, 2H), 7.21–7.16 (m, 2H), 3.86 (t, *J* = 7.3 Hz, 2H), 3.06 (t, *J* = 7.3 Hz, 2H). ^13^C (125 MHz, MeOD) δ ppm 159.8, 155.8, 149.3, 147.7 (q, *J* = 1.7 Hz), 138.6, 130.1, 126.4, 125.4, 123.9, 120.7, 120.5 (q, *J* = 255.1 Hz), 113.6, 42.2, 33.9. ^19^F (470 MHz, MeOD) δ ppm −59.6 (s, 3F). HRMS (EI), M + 1 calculated for C_17_H_13_ClF_3_N_3_O, 368.0772, found 368.0767.

#### (7-Bromoquinazolin-4-yl){2-[4-(trifluormethoxy)phenyl]ethyl}amine, 21a

Following general procedure A, using 4-chloro-7-bromoquinazoline (**21**, 75 mg, 0.30 mmol), 2-[4-(trifluoromethoxy)phenyl]ethylamine (62 mg, 0.30 mmol), and potassium carbonate (41 mg, 0.30 mmol) gave **21a** as off-white crystals (52 mg, 42%). mp 151.8–152.8 °C; ^1^H (500 MHz, CDCl_3_) δ ppm 8.68 (s, 1H), 8.06 (s, 1H), 7.56 (s, 2H), 7.32–7.26 (m, 2H), 7.20 (d, *J* = 8.0 Hz, 2H), 6.18 (br.s, 1H), 3.97 (dt, *J* = 6.9, 6.1 Hz, 2H), 3.09 (t, *J =* 7.0 Hz, 2H). ^13^C (125 MHz, CDCl_3_) δ ppm 159.4, 155.7, 149.4, 148.1 (q, *J* = 1.6 Hz), 137.4, 130.4, 130.1, 129.7, 127.4, 122.2, 121.3, 120.5 (q, *J* = 257.0 Hz), 113.4, 42.5, 34.5. ^19^F (470 MHz, CDCl_3_) δ ppm *−*57.9 (s, 3F). HRMS (EI), M + 1 calculated for C_17_H_13_BrF_3_N_3_O, 412.0267, found 412.0246.

#### {2-[4-(Trifluoromethoxy)phenyl]ethyl}[7-(trifluoromethyl)quinazolin-4-yl]amine, 22a

Following general procedure A, using 7-(trifluoromethyl)-4-chloroquinazoline (**22**, 100 mg, 0.45 mmol), 2-[4-(trifluoromethoxy)phenyl]ethylamine (93 mg, 0.45 mmol), and potassium carbonate (69 mg, 0.50 mol) gave **22a** as off-white crystals (82 mg, 55%). mp 149.7–152.3 °C; ^1^H (500 MHz, MeOD) δ ppm 8.44 (d, *J* = 8.4 Hz, 1H), 8.41–8.37 (m, 1H), 8.01 (s, 1H), 7.86 (dd, *J* = 8.4, 1.5 Hz, 1H), 7.42 (dt, *J* = 8.6, 2.8 Hz, 2H), 7.31–7.25 (m, 2H), 3.22 (t, *J* = 7.7 Hz, 2H), 3.02 (t, *J* = 7.7 Hz, 2H). ^13^C (125 MHz, MeOD) δ ppm 160.4, 148.3 (q, *J* = 1.8 Hz), 147.3, 135.8, 135.8 (q, *J* = 32.9 Hz), 130.2, 127.7, 125.0, 123.4 (q, *J* = 272.4 Hz), 123.1 (q, *J* = 3.6 Hz), 122.8 (m), 121.2, 120.5 (q, *J* = 255.5 Hz), 119.5, 40.3, 32.4. ^19^F (470 MHz, MeOD) δ ppm −59.6 (s, 3F), −64.8 (s, 3F). HRMS (EI), M + 1 calculated for C_18_H_13_F_6_N_3_O, 402.1036, found 402.1025.

#### (7-Methoxyquinazolin-4-yl){2-[4-(trifluormethoxy)phenyl]ethyl}amine, 23a

Following general procedure A, using 7-(methoxy)-4-chloroquinazoline (**23**, 100 mg, 0.54 mmol), 2-[4-(trifluoromethoxy)phenyl]ethylamine (110 mg, 0.54 mmol), and potassium carbonate (82 mg, 0.59 mol) gave **23a** as colorless crystals (68 mg, 51%). mp 158.0–159.0 °C; ^1^H (500 MHz, MeOD) δ ppm 8.40 (s, 1H), 7.94 (d, *J* = 9.1 Hz, 1H), 7.36 (dt, *J* = 8.7, 2.8 Hz, 2H), 7.21–7.16 (m, 2H), 7.11 (dd, *J* = 9.1, 2.6 Hz, 1H), 7.08 (d, *J* = 2.5 Hz, 1H), 3.93 (s, 3H), 3.83 (t, *J* = 7.4 Hz, 2H), 3.05 (t, *J* = 7.4 Hz, 2H). ^13^C (125 MHz, MeOD) δ ppm 163.5, 159.6, 155.0, 150.6, 147.7 (q, *J* = 1.7 Hz), 138.7, 130.1, 123.5, 120.7, 120.6 (q, *J* = 255.1 Hz), 117.3, 109.1, 105.5, 54.7, 42.1, 34.1. ^19^F (470 MHz, MeOD) δ ppm −59.5 (3F). HRMS (EI), M + 1 calculated for C_18_H_16_F_3_N_3_O_2_, 364.1267, found 364.1259.

#### (2-Methylquinazolin-4-yl){2-[4-(trifluormethoxy)phenyl]ethyl}amine, 24a

Following general procedure A, using 4-chloro-2-methylquinazoline (**24**, 100 mg, 0.56 mmol), 2-[4-(trifluoromethoxy)phenyl]ethylamine (115 mg, 0.56 mmol), and potassium carbonate (77 mg, 0.56 mmol) gave **24a** as a pale yellow solid (153 mg, 79%). mp 124.4–127.2 °C; ^1^H (500 MHz, CDCl_3_) δ ppm 7.81 (d, *J =* 8.3 Hz, 1H), 7.74–7.68 (m, 1H), 7.60 (d, *J =* 8.1 Hz, H), 7.42–7.37 (m, 2H), 7.20 (d, *J =* 8.0 Hz, 2H), 5.91 (br.s, 1H), 3.96 (dt, *J =* 6.9, 6.0 Hz, 2H), 3.07 (t, *J =* 7.0 Hz, 2H), 2.69 (s, 3H). ^13^C (125 MHz, CDCl_3_) δ ppm 164.3, 159.3, 149.4, 148.0 (q, *J* = 1.7 Hz), 137.8, 132.7, 130.2, 127.5, 125.3, 121.2, 120.5 (q, *J* = 256.9 Hz), 120.3, 112.8, 42.2, 34.7, 26.5. ^19^F (470 MHz, CDCl_3_) δ ppm −57.9 (s, 3F). HRMS (EI), M + 1 calculated for C_18_H_16_F_3_N_3_O, 348.1318, found 348.1335.

#### (2-Cyclopropylquinazolin-4-yl){2-[4-(trifluoromethoxy)phenyl]ethyl}amine, 25a

Following general procedure A, using 4-chloro-2-cyclopropylquinazolne (**25**, 100 mg, 0.49 mmol), 2-[4-(trifluoromethoxy)phenyl]ethylamine (100 mg, 0.49 mmol) and potassium carbonate (67 mg, 0.48 mmol) gave **25a** as a tan solid (119 mg, 65%). mp 205.1–205.5 °C; ^1^H (500 MHz, MeOD) δ ppm 8.24 (d, *J =* 8.5 Hz, 1H), 8.02–7.97 (m, 1H), 7.74–7.68 (m, 2H), 7.36 (dt, *J =* 8.6, 2.7 Hz, 2H), 7.20 (d, *J =* 7.8 Hz, 2H), 3.99 (t, *J =* 7.1 Hz, 2H), 3.08 (t, *J =* 7.1 Hz, 2H), 2.23–2.16 (m, 1H), 1.44–1.32 (m, 4H). ^13^C (125 MHz, MeOD) δ ppm 166.5, 160.8, 147.9, 138.3, 137.9, 135.7, 130.2, 127.7, 123.3, 120.9, 120.5 (q, *J* = 255.2 Hz), 118.0, 111.9, 42.9, 33.8, 14.5, 11.2. ^19^F (470 MHz, MeOD) δ ppm −59.6 (s, 3F). HRMS (EI), M + 1 calculated for C_20_H_18_F_3_N_3_O, 374.1475, found 374.1493.

#### (7-Fluoro-2-methylquinazolin-4-yl){2-[4-(trifluormethoxy)phenyl]ethyl}amine, 26a

Following general procedure A, using 4-chloro-7-fluoro-2-methylquinazoline (**26**, 75 mg, 0.37 mmol), 2-[4-(trifluoromethoxy)phenyl]ethylamine (77 mg, 0.37 mmol), and potassium carbonate (52 mg, 0.37 mmol) gave **26a** as a white solid (58 mg, 43%). mp 121.7–124.7 °C; ^1^H (500 MHz, CDCl_3_) δ ppm 14.78 (s, 1H), 10.62 (s, 1H), 9.10 (dd, *J* = 9.1, 5.2 Hz, 1H), 7.84 (dd, *J* = 8.6, 2.4 Hz, 1H), 7.30–7.22 (m, 1H), 7.19–7.13 (m, 2H), 7.06–7.02 (m, 2H), 4.14 (q, *J* = 6.9 Hz, 2H), 3.22 (t, *J* = 7.4 Hz, 2H), 2.77 (s, 3H). ^13^C (125 MHz, CDCl_3_) δ ppm 166.1 (d, *J* = 259.4 Hz), 162.0, 160.0, 149.3 (q, *J* = 2.0 Hz), 140.7, 140.5 (d, *J* = 12.9 Hz), 129.8, 128.7 (d, *J* = 10.3 Hz), 127.4, 121.4, 120.4 (q, *J* = 257.1 Hz), 119.1, 116.9 (d, *J* = 23.9 Hz), 108.6, 104.7 (d, *J* = 25.6 Hz), 43.1, 34.6, 22.4. ^19^F (470 MHz, CDCl_3_) δ ppm −57.8 (s, 3F), −98.3 (ddd, *J* = 4.8, 8.1, 8.1 Hz, 1F). HRMS (EI), M + 1 calculated for C_18_H_15_F_4_N_3_O, 366.1224, found 366.1199.

### 2.2. Biological Assessments

*Mycobacterium bovis* BCG and *Mycobacterium tuberculosis* strains were routinely cultured in Middlebrook 7H9 medium (Becton Dickson and Company Limited, Franklin Lakes, NJ, USA) supplemented with 0.05% tween 80, 0.5% glycerol, bovine serum albumin fraction V, D-glucose, and NaCl. *M. tuberculosis* clinical isolate N0145 was a gift from Sebastien Gagneux (Swiss Tropical and Public Health Institute at the University of Basel). Prior to use, bacteria cells were pelleted and resuspended in medium without glycerol and adjusted to a cell density of circa 10^7^ CFU/mL. The test compounds were tested in the presence or absence of Q203. Prior to the assay, Q203 or DMSO (solvent control) was added to the bacteria cultures. Q203 was used in excess (at 100 nM) to completely inhibit the function of the cytochrome *bcc:aa*_*3*_, thereby revealing the activity of cyt-*bd* in the assay. The Q203 ATP IC_50_ for *M. bovis* BCG, *M. tuberculosis* H37Rv, and *M. tuberculosis* N0145 are 2.6, 1.0, and 2.5 nM, respectively (Supplementary Materials Figure S61). The test compounds were tested in eight concentrations, starting at 25 µM, in two-fold dilutions (0.2–25.0 µM) in the presence or absence of 100 nM Q203. 1 µL of test compounds of varying concentrations was added to each well of 96-well white plates, and 100 µL of bacterial culture was subsequently added. The assay plates were incubated at 37 °C for 15 h, after which the BacTiter-Glo™ (Promega, Madison, WI, USA) reagent was added. Following a 12-min incubation, the luminescence of each plate was measured using a BioTek Cytation 3 Cell Imaging Multiple mode reader. IC_50_ values were determined using GraphPad Prism 9.

## 3. Results and Discussion

Our initial efforts were to probe the effect of alteration and modification of the phenethylaniline moiety around a fixed quinazoline core. This was done by syntheses of 10 compounds and screening against *M. bovis* BCG and Mtb strains (H37Rv and N0145) to assess activity against mycobacterial cyt-*bd* (Table 1). A representative compound from this set (**7a**) served as the foundation toward the design of a second set of 11 compounds that focused on modification of the quinazoline core and were subsequently screened for cyt-*bd* activity (Table 2).

### 3.1. Chemical Syntheses of the First Set of N-Phenethylquinazolin-4-Amines (**6a**–**15a**) and the Second Set of N-Phenethylquinazolin-4-Amines (**16a**–**26a**)

**Scheme 1.**
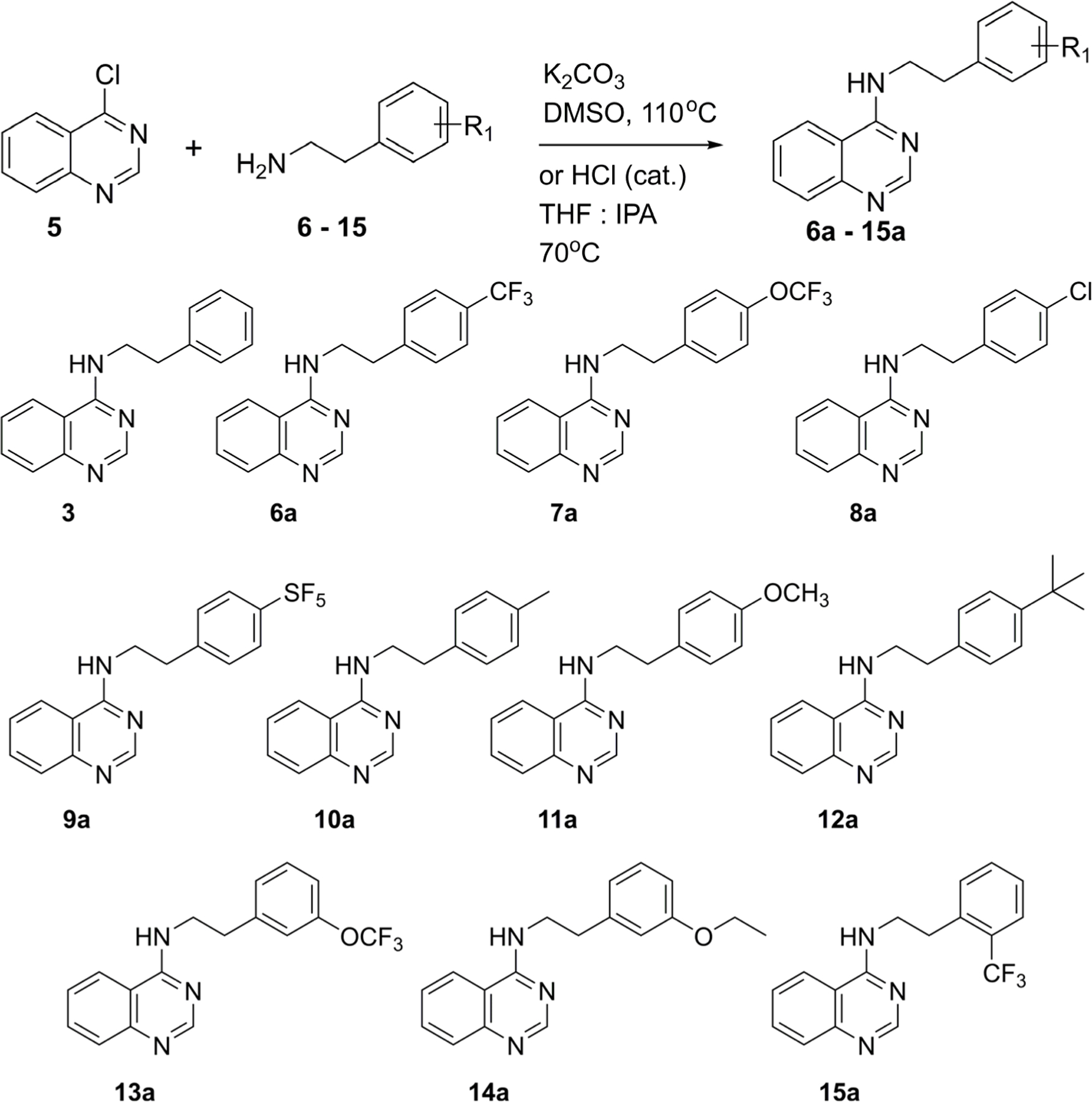
S_N_Ar conditions to prepare the first set of *N*-phenethylquinazolin-4-amines (**6a**–**15a**) for screening against cyt-*bd* of *M. bovis* BCG and *M. tuberculosis* H37Rv.

The first set of compounds (**6a**–**15a**) were prepared by reaction of 4-chlorquinazoline (**5**) with ten commercially available amines (**6**–**15**) using S_N_Ar conditions (basic or acidic, Scheme 1). These compounds were evaluated for their activity against cyt-*bd* in *M. bovis* BCG using an ATP readout (Table 1).

**Scheme 2.**
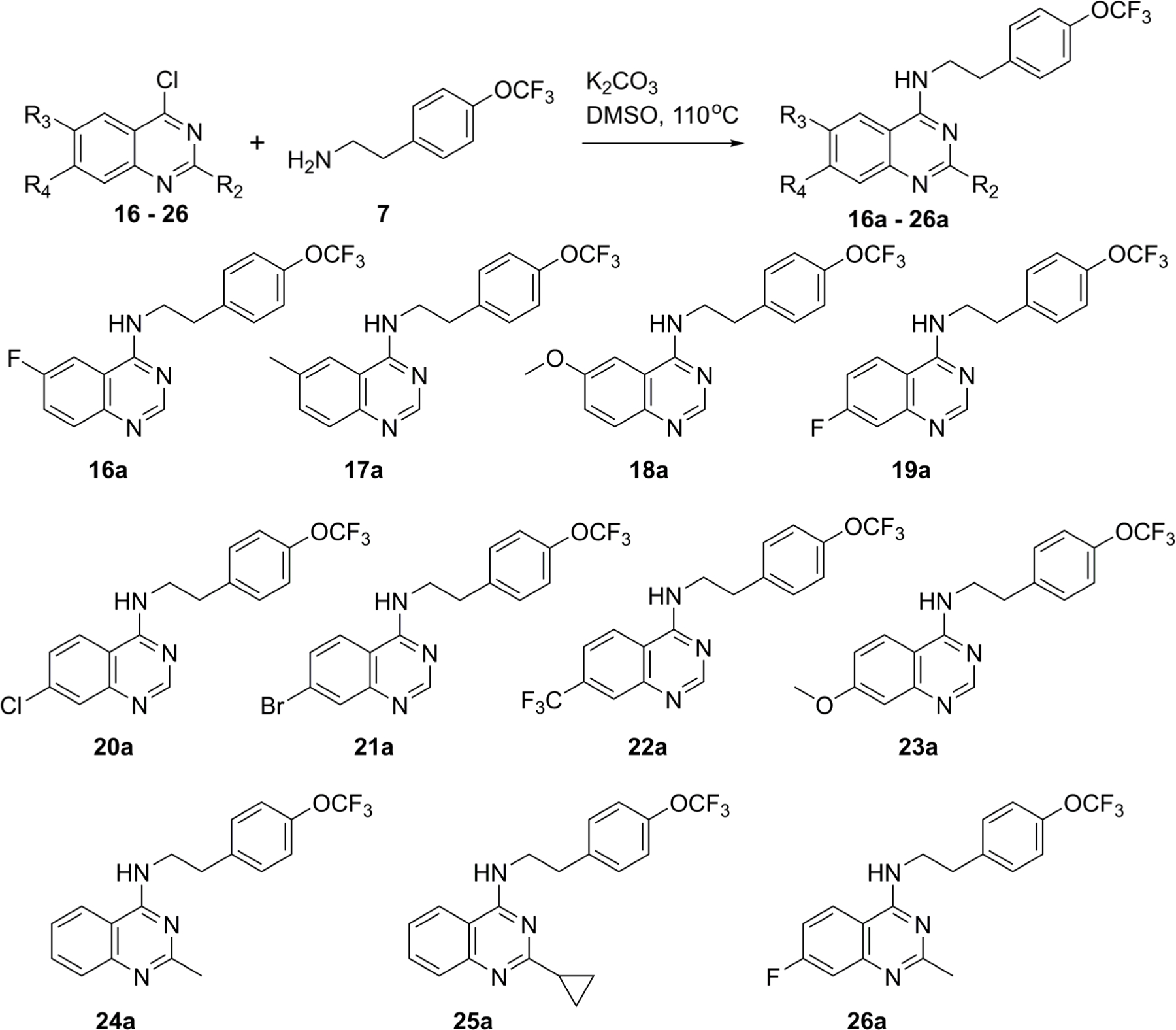
S_N_Ar conditions to prepare the second set of *N*-phenethylquinazolin-4-amines (**16a**–**26a**) for screening against cyt-*bd* of *M. bovis* BCG and *M. tuberculosis* H37Rv.

The second set of compounds (**16a**–**26a**) was prepared by reacting eight functionalized 4-chloroquinazolines (**16**–**26**) with 4-(trifluoromethoxy)benzylamine (**7**) using standard S_N_Ar conditions (Scheme 2). As with the first set, the activity was determined against cyt-*bd* in *M. bovis* BCG and *M. tuberculosis* H37Rv by ATP readout (Table 2).

### 3.2 Structure–Activity Relationship Studies

The structure–activity-relationship (SAR) studies revealed that the cyt-*bd* of *M. bovis* BCG was more sensitive to inhibition than *M. tuberculosis* H37Rv (Table 1). This mirrors the trend observed previously and is indicative that there is a greater expression of cyt-*bd* in the lab-adapted *M. tuberculosis* H37Rv strain relative to the *M. bovis* BCG or “fresh” Mtb clinical isolate N0145 [13]. This is further supported by the activity of aurachin D [7], a known synergistic cyt-*bd* inhibitor of Mtb, which had greater activity against *M. bovis* BCG in our ATP assay relative to *M. tuberculosis* H37Rv (2.9 µM vs. 5.5 µM, respectively; Table 1). In addition, based on our assay, compounds **6a**–**15a** are all specific inhibitors of cyt-*bd* as they show no activity against wild-type (WT) *M. bovis* BCG or *M. tuberculosis* H37Rv (Mtb-H37Rv), whereas the positive control bedaquiline (BDQ), a selective ATP synthase inhibitor, remains very potent against both strains (Table 1). Interestingly, aurachin D showed the expected synergy with Q203 against mycobacterial strains but was unexpectedly active against wild-type Mtb-H37Rv (Table 1).

SAR trends revealed that the pendant aryl group can accommodate a variety of steric and electronic changes and retain good activity (Table 1). Evaluation of the compounds with electron withdrawing substituents **6a** (*p*-CF_3_), **7a**, (*p*-OCF_3_), **8a** (*p*-Cl), **9a** (*p*-SF_5_), indicated that **7a** is the most active compound with IC_50_ values of 2.1 µM against *M. bovis* BCG and 11 µM against Mtb H37Rv. We explored electron withdrawing groups at the *meta*- and *ortho*-positions of the pendant aryl group by comparing compound sets **7a** (*para*-OCF_3_) and **13a** (*meta*-OCF_3_) and **6a** (*para*-CF_3_) and **15a** (*ortho*-CF_3_). We found that the *meta*-OCF_3_ compound (**13a**) position was less active compared to the *para*-OCF_3_ (**17a**). Whereas incorporation of substituents in the *ortho*-position gave slightly improved potency against *M. bovis* but equal activity (within error) in Mtb-H37Rv as demonstrated by comparing **6a** and **15a**. When evaluating the compounds with electron donating substituents, **10a** (*p*-CH_3_), **11a** (*p*-OCH_3_), and **12a** (*p*-*tert*-butyl), **12a** was found to be significantly more active with IC_50_ values of 0.8 µM against *M. bovis* BCG and 5.8 µM against Mtb H37Rv. That is an improvement over thieno[3,2-*d*]pyrimidine (**1**) and activity on par with the optimized quinazoline ND-11992 (**2**). This is particularly interesting as it follows the Topliss aromatic substitution decision tree pattern [23] where one would first evaluate the phenyl (**3**), then *para*-chloro (**8a**) and if equally potent (as it is in our assays) then prepare the *tert*-butyl (**12a**) to derive the most potent compound. While the Topliss approach can often lead to compounds with improved activity it also directs the syntheses of compounds which often have metabolic liabilities (CH_3_, OCH_3_, *t*-butyl) or toxic pharmacophores (NH_2_, NO_2_, I). For this reason, we chose **7a** which bears a *para*-trifluoromethoxy group (which is prevalent in various anti-TB drugs such as delamanid, pretomanid, and telacebec) as the basis for our second set of compounds (Table 2) because it possesses good activity (IC_50_*′*s of 2.1 and 11 µM against BCG and Mtb-H37Rv, respectively).

The SAR trends for alteration of the quinazoline core suggest an interplay between both steric and electronic effects (Table 2). For instance, the 6-position was probed with three substituents-fluoro (**16a**), methyl (**17a**) and methoxy (**18a**)—which resulted in a wide spectrum of activity. The 6-methyl analog (**17a**) was the most active against *M. bovis* BCG (IC_50_ of 7.5 µM) but weakly active against Mtb H37Rv (IC_50_ of 22 µM). Whereas the fluoro (**16a**) and methoxy (**18a**) analogs had diminished activity against *M. bovis* BCG (IC_50_ of 13 µM and 27 µM, respectively) and both were >25 µM against Mtb H37Rv. The 7-position was explored with five different substituents-fluoro (**19a**), chloro (**20a**), bromo (**21a**), trifluoromethoxy (**22a**) and methoxy (**23a**)—resulting in only two active compounds (**19a** and **21a**). The 7-fluoro analog (**19a**) displayed good activity against both mycobacterial strains (IC_50_ of 0.8 µM and 7.6 µM; respectively) and the 7-bromo (**21a**) showed much better activity against *M. bovis* BCG than Mtb H37Rv (IC_50_ of 7 µM and 20 µM; respectively). The most interesting SAR was observed with substitution of the quinazoline 2-position with methyl (**24a**) and cyclopropyl (**25a**) groups. Both compounds displayed good activity range (3–12 µM) with or without cyt-*bcc:aa*_*3*_ inhibition, suggesting possible dual modes of action. Lastly, one compound (**26a**) bore both 7-fluoro and 2-methyl quinazoline substituents which biased activity back towards cyt-*bd* (IC_50_ of 7 and 17 µM with Q203, and 28 µM and 24 µM without Q203; respectively)

Our previous work had shown that the laboratory adapted Mtb H37Rv strain over expresses cyt-*bd* resulting in higher IC_50_ values than those observed using a clinical Mtb isolate [24]. These higher IC_50_ values render compound ranking more challenging. To gain greater insight on the activity of these compounds, they were re-screened against the clinical Mtb isolate N0145 strain. As observed previously with the thieno[3,2-*d*]pyrimidin-4-amine class of cyt-*bd* oxidase inhibitors [13], these compounds displayed greatly improved activity against the Mtb clinical isolate likely due to lower expression of cyt-*bd* within clinical isolates as compared to lab-adapted strains like H37Rv [12]. Two compounds, **7a** and **12a**, from the first set were more active than or comparable in activity to aurachin D (0.1 and 1.1 µM vs. 1.5 µM, respectively). While six additional compounds (**6a**, **9a**, **10a**, **13a**–**15a**) had IC_50_ values below 5 µM against N0145-Mtb. In the second set of compounds, only **19a** had activity superior to aurachin D (0.2 µM vs. 1.5 µM, respectively) and four additional compounds (**17a**, **21a**, **24a**–**26a**) had activity below 5 µM. The two most potent compounds, **12a** and **19a**, had slightly improved activity relative to ND-11992 which was active in the murine infection model of tuberculosis. However, preliminary metabolic stability assessment of these compounds against human and rat microsomes indicate much more rapid metabolism than that of ND-11992 (data not shown).

## 4. Conclusions

The described *N*-phenethylquinazolin-4-amine class of compounds are mycobacterial cyt-*bd* inhibitors. Through focused SAR, the activity of the hit compound **3** (IC_50_ = 6.9 µM against Mtb N0145) was improved with 14 of the 22 analogs (64%) and two, **12a** and **19a**, had sub-micromolar activity (0.1 and 0.2 µM against Mtb N0145, respectively). Future work includes identification of more efficacious compounds through additional SAR around the quinazoline core. These results will be reported in due course.

## 5. Patents

US Provisional Application No. 62/783,984.

## Supplementary Material

SI Appl. Sci. 2021, 11(19), 9092; https://doi.org/10

## Figures and Tables

**Table 1.** In vitro activity of phenethyl-quinazolin-4-yl-amines (**3**, **6a**–**15a**) and control compounds bedaquiline (BDQ), ND-11992 (**2**), and aurachin D against three mycobacterial strains (*M. bovis* BCG, *M. tuberculosis* H37Rv, and *M. tuberculosis* N0145).

Compound	Clog *p*	Mol. Wt.	BCG		Replicating ATP IC_50_ (µM) H37Rv		N0145	
			−Q203	+Q203	−Q203	+Q203	–Q203	+Q203
**3**	3.98	249.31	>25	11.2 *±* 1.574	>25	27.2 *±* 4.613	>25	6.9 *±* 0.752
**6a**	4.86	317.31	>25	5.1 *±* 0.501	>25	16.9 *±* 3.758	>25	2.6 *±* 0.581
**7a**	5.01	333.31	>25	2.1 *±* 0.205	>25	11.0 *±* 2.081	>25	1.1 *±* 0.719
**8a**	4.69	283.76	>25	12.5 *±* 1.952	>25	29.7 *±* 5.336	>25	8.2 *±* 0.808
**9a**	5.21	375.36	>25	3.9 *±* 0.490	>25	14.5 *±* 2.691	>25	2.3 *±* 0.853
**10a**	4.48	263.34	>25	6.7 *±* 1.116	>25	20.7 *±* 3.624	>25	3.8 *±* 0.813
**11a**	3.9	279.34	>25	13.7 *±* 2.107	>25	>25	>25	7.1 *±* 0.653
**12a**	5.81	305.42	>25	0.8 *±* 0.144	>25	5.8 *±* 0.616	>25	0.1 *±* 0.017
**13a**	5.01	333.31	>25	7.1 *±* 0.849	>25	22.5 *±* 3.912	>25	3.8 *±* 0.733
**14a**	4.43	293.36	>25	5.8 *±* 0.867	>25	19.3 *±* 5.703	>25	3.2 *±* 0.204
**15a**	4.86	317.31	>25	3.9 *±* 0.311	>25	17.0 *±* 2.945	>25	3.2 *±* 0.795
**1**	5.85	313.44	>25	5.8 *±* 1.06	>25	18.9 *±* 9.03	>25	8.5 *±* 2.38
**2**	6.69	381.36	>25	0.7 *±* 0.103	>25	6.3 *±* 0.349	>25	0.3 *±* 0.049
**BDQ**	7.25	555.52	0.17 *±* 0.019	0.11 *±* 0.026	0.06 *±* 0.019	0.04 *±* 0.024	0.05 *±* 0.003	0.03 *±* 0.001
**Aurachin D**	6.79	363.55	>25	2.9 *±* 0.317	5.5 *±* 1.107	7.3 *±* 1.973	>25	1.5 *±* 0.065

IC_50_ values were determined by ATP depletion in the presence and absence of Q203 (see Section 2.2 for details). IC_50_ values were determined using GraphPad Prism 9. The values reflected in the table represent the average and standard deviation, which were calculated from the IC_50_ values of replicates from two experimental repeats. cLog*P* was calculated by Perkin Elmer ChemDraw Professional 16.0. Bedaquiline (BDQ), ND-11992 (**2**) and aurachin D were used as positive control compounds.

**Table 2.** Screening of the second set of *N*-phenethylquinazolin-4-amines **16a**–**26a** against cyt-*bd* of *M. bovis* BCG and *M. tuberculosis* H37Rv by ATP readout along with known inhibitors ND-11992 (**2**), aurachin D and bedaquiline (BDQ) as positive controls.

Compound	Clog *p*	Mol. Wt.	BCG		Replicating ATP IC_50_ (µM) H37Rv		N0145	
			−Q203	+Q203	−Q203	+Q203	–Q203	+Q203
**16a**	5.19	351.3	>25	13.1 *±* 0.651	>25	28.8 *±* 5.054	>25	7.1 *±* 0.207
**17a**	5.51	347.33	>25	7.5 *±* 0.580	>25	22.3 *±* 5.796	>25	3.4 *±* 0.559
**18a**	5.41	363.33	>25	26.9 *±* 1.231	>25	>25	>25	17.1 *±* 0.220
**19a**	5.19	351.3	>25	0.8 *±* 0.077	>25	7.6 *±* 1.079	>25	0.2 *±* 0.057
**20a**	5.76	367.75	>25	>25	>25	>25	>25	>25
**21a**	5.91	412.21	>25	6.7 *±* 0.591	>25	19.5 *±* 2.356	>25	4.9 *±* 0.226
**22a**	5.95	401.31	>25	>25	>25	>25	>25	>25
**23a**	5.41	363.33	>25	>25	>25	>25	>25	>25
**24a**	5.51	347.33	12.2 *±* 0.737	3.1 *±* 0.369	7.5 *±* 1.025	11.7 *±* 1.252	8.2 *±* 0.654	2.9 *±* 0.140
**25a**	5.95	373.37	8.5 *±* 1.529	4.0 *±* 0.604	7.6 *±* 1.026	11.7 *±* 1.036	7.3 *±* 1.052	2.1 *±* 0.347
**26a**	5.69	365.32	27.6 *±* 9.513	6.5 *±* 0.751	23.6 *±* 7.807	16.9 *±* 3.822	32.7 *±* 2.700	4.6 *±* 0.219
**2**	6.69	381.36	>25	0.7 *±* 0.103	>25	6.3 *±* 0.349	>25	0.3 *±* 0.049
**BDQ**	7.25	555.52	0.17 *±* 0.019	0.11 *±* 0.026	0.06 *±* 0.019	0.04 *±* 0.024	0.05 *±* 0.003	0.03 *±* 0.001
**Aurachin D**	6.79	363.55	>25	2.9 *±* 0.317	5.5 *±* 1.107	7.3 *±* 1.973	>25	1.5 *±* 0.065

IC_50_ values were determined by ATP depletion in the presence and absence of Q203 (see 2.2 for details). IC_50_ values were determined using GraphPad Prism 9. The values given in the table represent the average and standard deviation, which were calculated from the IC_50_ values of replicates from two experimental repeats. cLog*P* was calculated by Perkin Elmer ChemDraw Professional 16.0. Bedaquiline (BDQ), ND-11992 (**2**) and aurachin D were used as positive control compounds.
