# Supplementary material for: Syntheses and Structure–Activity Relationships of N-Phenethyl-Quinazolin-4-yl-Amines as Potent Inhibitors of Cytochrome bd Oxidase in Mycobacterium tuberculosis
Source: Appl Sci (Basel). Author manuscript; Available in PMC 2023 Jan 24. (PMC9873234; doi:10.3390/app11199092)
Supplement: SI Appl. Sci. 2021, 11(19), 9092; https://doi.org/10 [file NIHMS1814612-supplement-SI_Appl__Sci__2021__11_19___9092__https___doi_org_10.pdf]

applied sciences

MDPI

Supplementary Material

Syntheses and Structure Activity Relationships of N-phenethyl-quinazolin-4-yl-amines as potent inhibitors of cytochrome *bd* oxidase in *Mycobacterium tuberculosis*

Sarah M. Hopfner [1], Bei Shi Lee [2], Nitin P. Kalia [3], Marvin J. Miller [4], Kevin Pethe [2,5], and Garrett C. Moraski [1*]

[1] Department of Chemistry and Biochemistry, Montana State University, 103 Chemistry and Biochemistry Building, Bozeman, Montana 59717, USA; sarah.hopfner@student.montana.edu, garrett.moraski@montana.edu

[2] School of Biological Sciences, Nanyang Technological University, 637551, Singapore; kevin.pethe@ntu.edu.sg, beishi001@e.ntu.edu.sg

[3] Department of Biological Sciences National Institute of Pharmaceutical Education and Research, Hyderabad, Telangana 500037, India; kalianpk@gmail.com

[4] Department of Chemistry and Biochemistry, University of Notre Dame, 251 Nieuwland Science Hall, Notre Dame, Indiana 46556, USA; mmiller1@nd.edu

[5] Lee Kong Chian School of Medicine, Nanyang Technological University, Experimental Medicine Building, 59 Nanyang Drive, 636921, Singapore; kevin.pethe@ntu.edu.sg

* Correspondence: G.C.M. garrett.moraski@montana.edu

Table of Contents

1. *^{1}H NMR, ^{13}C NMR and ^{19}F NMR Spectrum of all compounds*

Figure S1 ^{1}H NMR Spectrum (MeOD, 500 MHz) of **3**……3
Figure S2 ^{13}C NMR Spectrum (MeOD, 125 MHz) of **3**……4
Figure S3 ^{1}H NMR Spectrum ($CDCl_3$, 500 MHz) of **6a**……5
Figure S4 ^{13}C NMR Spectrum ($CDCl_3$, 125 MHz) of **6a**……6
Figure S5 ^{19}F NMR Spectrum ($CDCl_3$, 470 MHz) of **6a**……7
Figure S6 ^{1}H NMR Spectrum ($CDCl_3$, 500 MHz) of **7a**……8
Figure S7 ^{13}C NMR Spectrum ($CDCl_3$, 125 MHz) of **7a**……9
Figure S8 ^{19}F NMR Spectrum ($CDCl_3$, 470 MHz) of **7a**……10
Figure S9 ^{1}H NMR Spectrum (MeOD, 500 MHz) of **8a**……11
Figure S10 ^{13}C NMR Spectrum (MeOD, 125 MHz) of **8a**……12
Figure S11 ^{1}H NMR Spectrum ($CDCl_3$, 500 MHz) of **9a**……13
Figure S12 ^{13}C NMR Spectrum ($CDCl_3$, 125 MHz) of **9a**……14
Figure S13 ^{19}F NMR Spectrum ($CDCl_3$, 470 MHz) of **9a**……15
Figure S14 ^{1}H NMR Spectrum ($CDCl_3$, 500 MHz) of **10a**……16
Figure S15 ^{13}C NMR Spectrum ($CDCl_3$, 125 MHz) of **10a**……17
Figure S16 ^{1}H NMR Spectrum ($CDCl_3$, 500 MHz) of **11a**……18
Figure S17 ^{13}C NMR Spectrum ($CDCl_3$, 125 MHz) of **11a**……19
Figure S18 ^{1}H NMR Spectrum ($CDCl_3$, 500 MHz) of **12a**……20
Figure S19 ^{13}C NMR Spectrum ($CDCl_3$, 125 MHz) of **12a**……21
Figure S20 ^{1}H NMR Spectrum ($CDCl_3$, 500 MHz) of **13a**……22
Figure S21 ^{13}C NMR Spectrum ($CDCl_3$, 125 MHz) of **13a**……23
Figure S22 ^{19}F NMR Spectrum ($CDCl_3$, 470 MHz) of **13a**……24

Figure S23 ^{1}H NMR Spectrum ($CDCl_3$, 500 MHz) of **14a**……25
Figure S24 ^{13}C NMR Spectrum ($CDCl_3$, 125 MHz) of **14a**……26
Figure S25 ^{1}H NMR Spectrum ($CDCl_3$, 500 MHz) of **15a**……27
Figure S26 ^{13}C NMR Spectrum ($CDCl_3$, 125 MHz) of **15a**……28
Figure S27 ^{19}F NMR Spectrum ($CDCl_3$, 470 MHz) of **15a**……29
Figure S28 ^{1}H NMR Spectrum ($CDCl_3$, 500 MHz) of **16a**……30
Figure S29 ^{13}C NMR Spectrum ($CDCl_3$, 125 MHz) of **16a**……31
Figure S30 ^{19}F NMR Spectrum ($CDCl_3$, 470 MHz) of **16a**……32
Figure S31 ^{1}H NMR Spectrum ($CDCl_3$, 500 MHz) of **17a**……33
Figure S32 ^{13}C NMR Spectrum ($CDCl_3$, 125 MHz) of **17a**……34
Figure S33 ^{19}F NMR Spectrum ($CDCl_3$, 470 MHz) of **17a**……35
Figure S34 ^{1}H NMR Spectrum ($CDCl_3$, 500 MHz) of **18a**……36
Figure S35 ^{13}C NMR Spectrum ($CDCl_3$, 125 MHz) of **18a**……37
Figure S36 ^{19}F NMR Spectrum ($CDCl_3$, 470 MHz) of **18a**……38
Figure S37 ^{1}H NMR Spectrum ($CDCl_3$, 500 MHz) of **19a**……39
Figure S38 ^{13}C NMR Spectrum ($CDCl_3$, 125 MHz) of **19a**……40
Figure S39 ^{19}F NMR Spectrum ($CDCl_3$, 470 MHz) of **19a**……41
Figure S40 ^{1}H NMR Spectrum (MeOD, 500 MHz) of **20a**……42
Figure S41 ^{13}C NMR Spectrum (MeOD, 125 MHz) of **20a**……43
Figure S42 ^{19}F NMR Spectrum (MeOD, 470 MHz) of **20a**……44
Figure S43 ^{1}H NMR Spectrum ($CDCl_3$, 500 MHz) of **21a**……45
Figure S44 ^{13}C NMR Spectrum ($CDCl_3$, 125 MHz) of **21a**……46
Figure S45 ^{19}F NMR Spectrum ($CDCl_3$, 470 MHz) of **21a**……47
Figure S46 ^{1}H NMR Spectrum (MeOD, 500 MHz) of **22a**……48
Figure S47 ^{13}C NMR Spectrum (MeOD, 125 MHz) of **22a**……49
Figure S48 ^{19}F NMR Spectrum (MeOD, 470 MHz) of **22a**……50
Figure S49 ^{1}H NMR Spectrum (MeOD, 500 MHz) of **23a**……51
Figure S50 ^{13}C NMR Spectrum (MeOD, 125 MHz) of **23a**……52
Figure S51 ^{19}F NMR Spectrum (MeOD, 470 MHz) of **23a**……53
Figure S52 ^{1}H NMR Spectrum ($CDCl_3$, 500 MHz) of **24a**……54
Figure S53 ^{13}C NMR Spectrum ($CDCl_3$, 125 MHz) of **24a**……55
Figure S54 ^{19}F NMR Spectrum ($CDCl_3$, 470 MHz) of **24a**……56
Figure S55 ^{1}H NMR Spectrum (MeOD, 500 MHz) of **25a**……57
Figure S56 ^{13}C NMR Spectrum (MeOD, 125 MHz) of **25a**……58
Figure S57 ^{19}F NMR Spectrum (MeOD, 470 MHz) of **25a**……59
Figure S58 ^{1}H NMR Spectrum ($CDCl_3$, 500 MHz) of **26a**……60
Figure S59 ^{13}C NMR Spectrum ($CDCl_3$, 125 MHz) of **26a**……61
Figure S60 ^{19}F NMR Spectrum ($CDCl_3$, 470 MHz) of **26a**……62

2. ATP Dose Response Curves of Q203

Figure S61 ATP dose response curves of Q203 in *M. bovis* BCG, *M. tuberculosis* H37Rv, and *M. tuberculosis* N0145……63

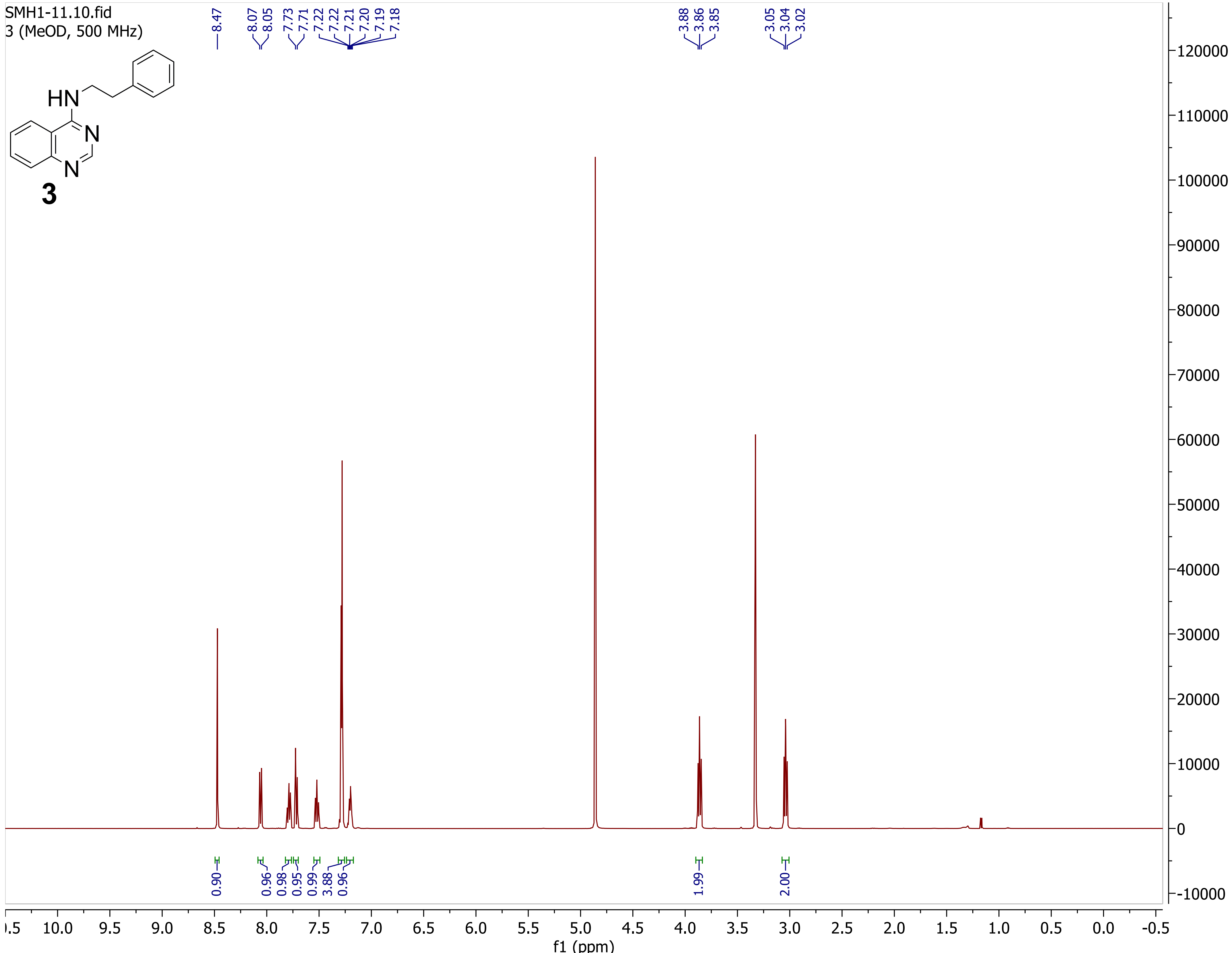

Figure S1 ^{1}H NMR Spectrum (MeOD, 500 MHz) of **3**

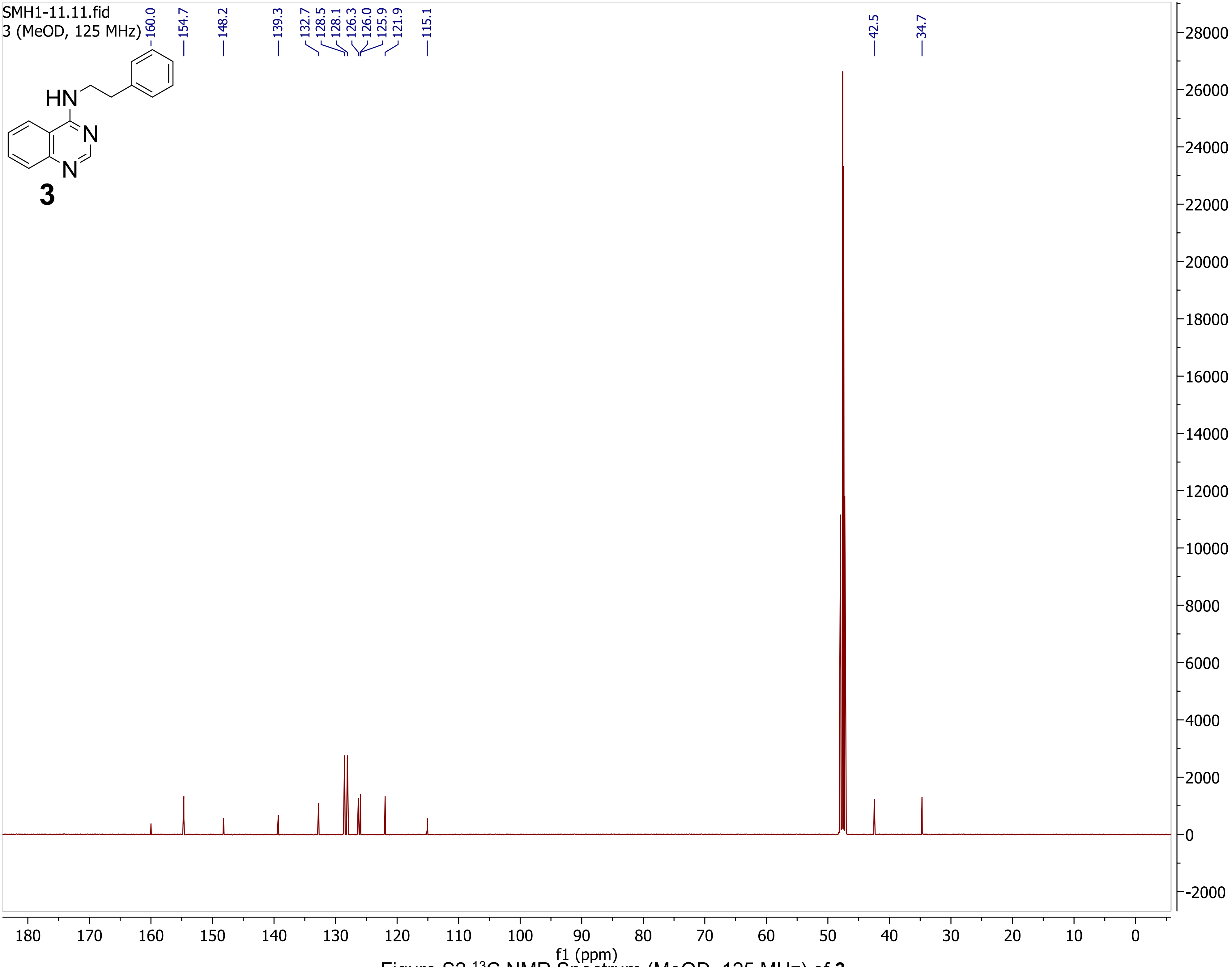

Figure S2 ^{13}C NMR Spectrum (MeOD, 125 MHz) of **3**

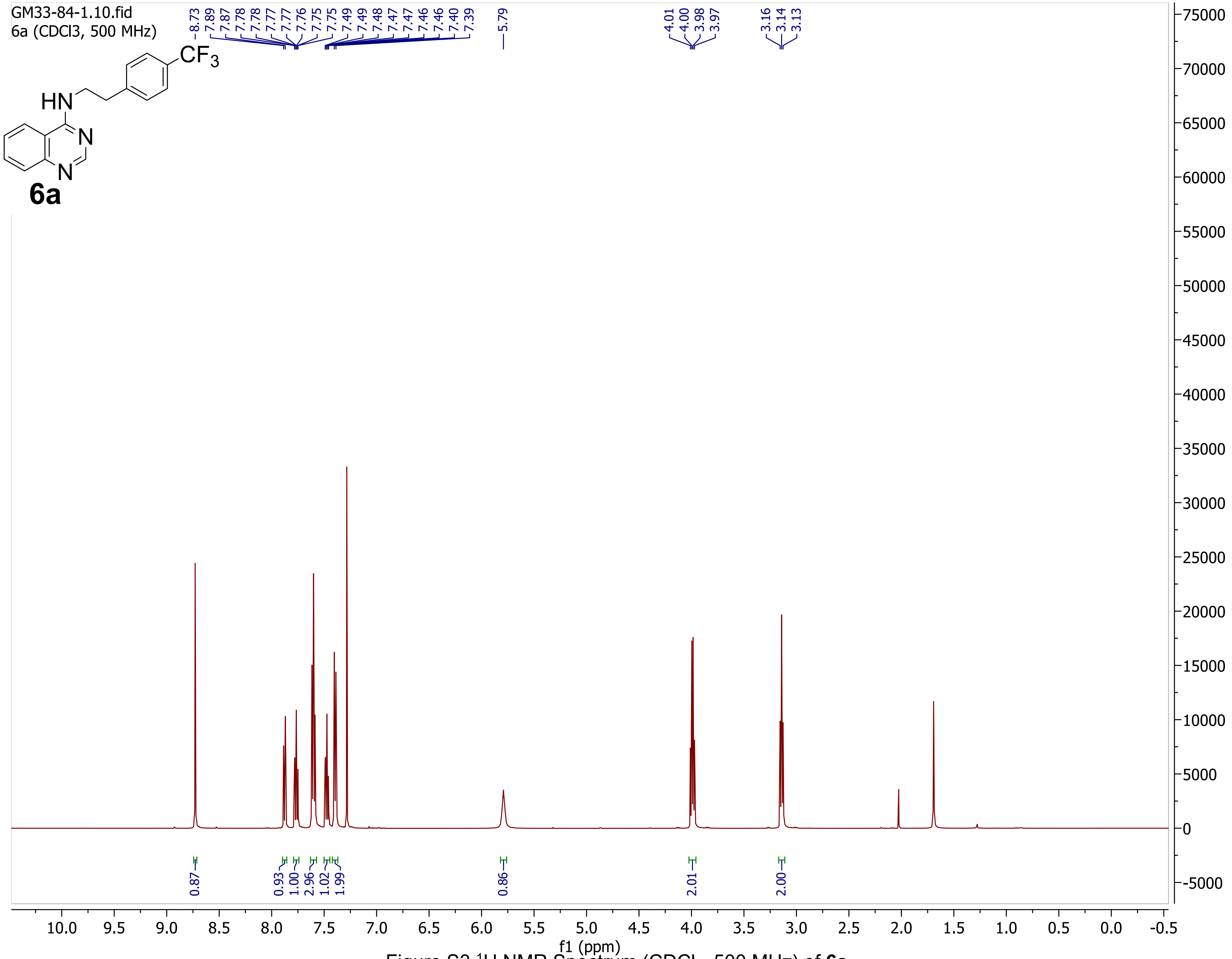

Figure S3 ^{1}H NMR Spectrum ($CDCl_3$, 500 MHz) of **6a**

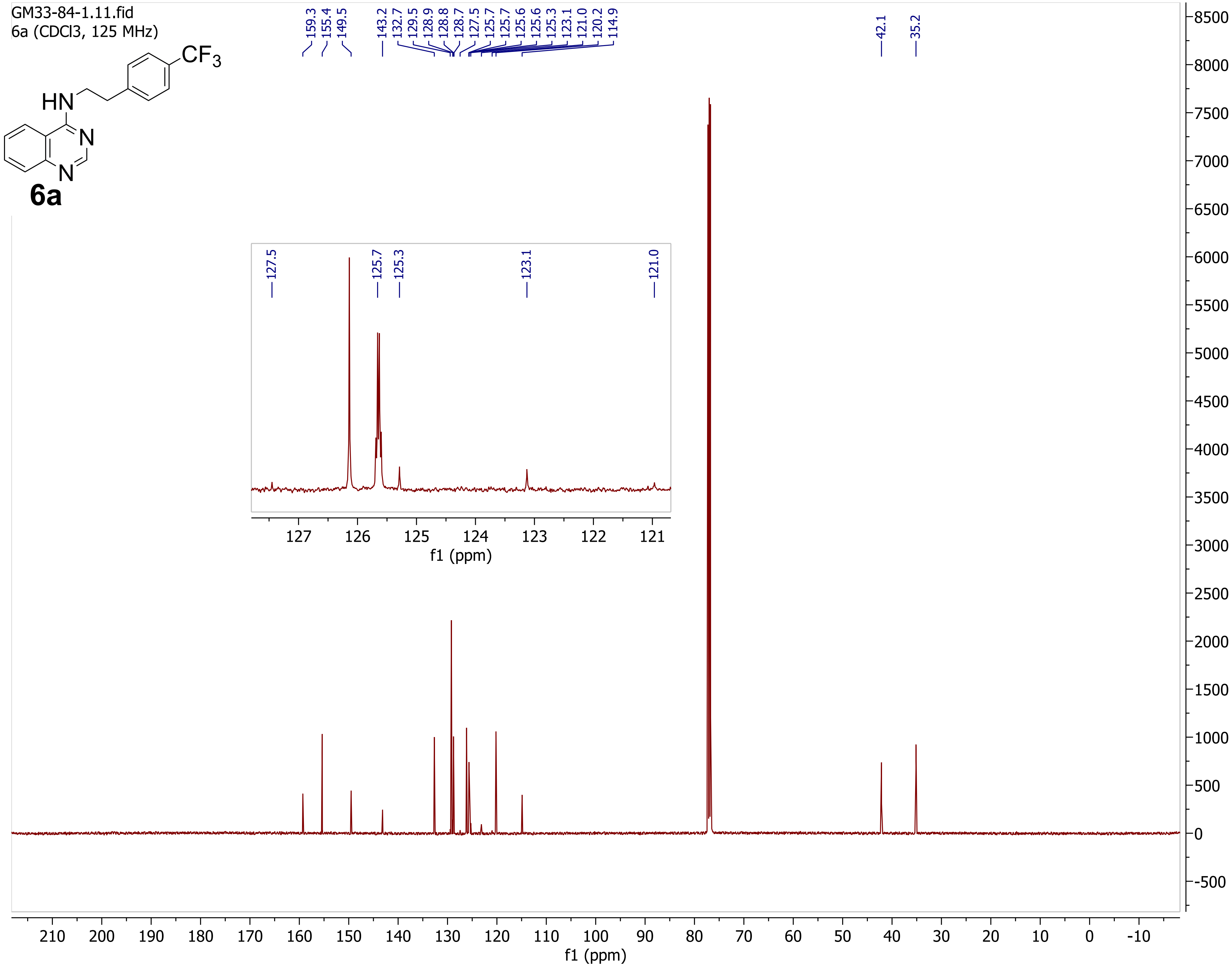

Figure S4 ^{13}C NMR Spectrum ($CDCl_3$, 125 MHz) of **6a**

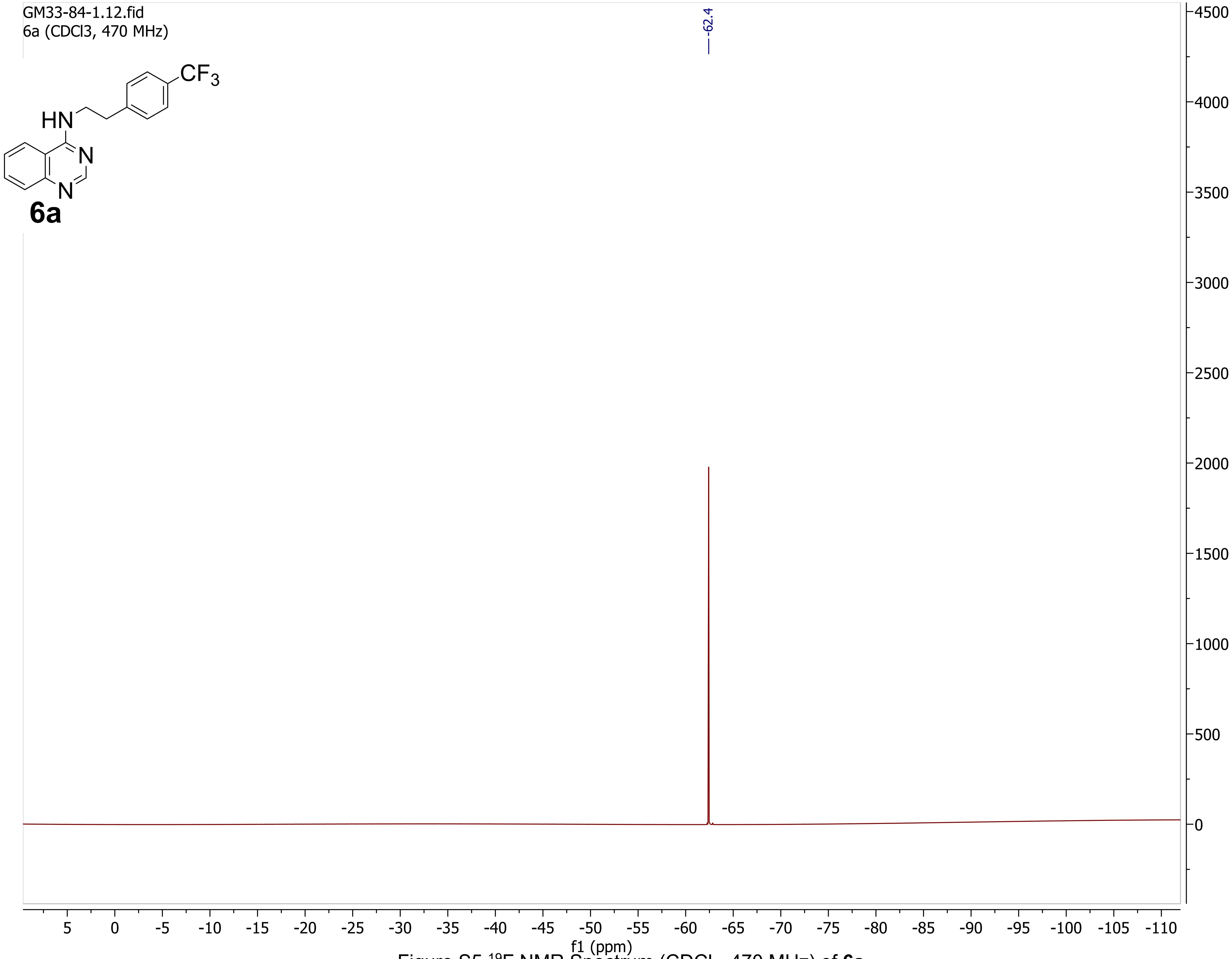

Figure S5 ^{19}F NMR Spectrum ($CDCl_3$, 470 MHz) of **6a**

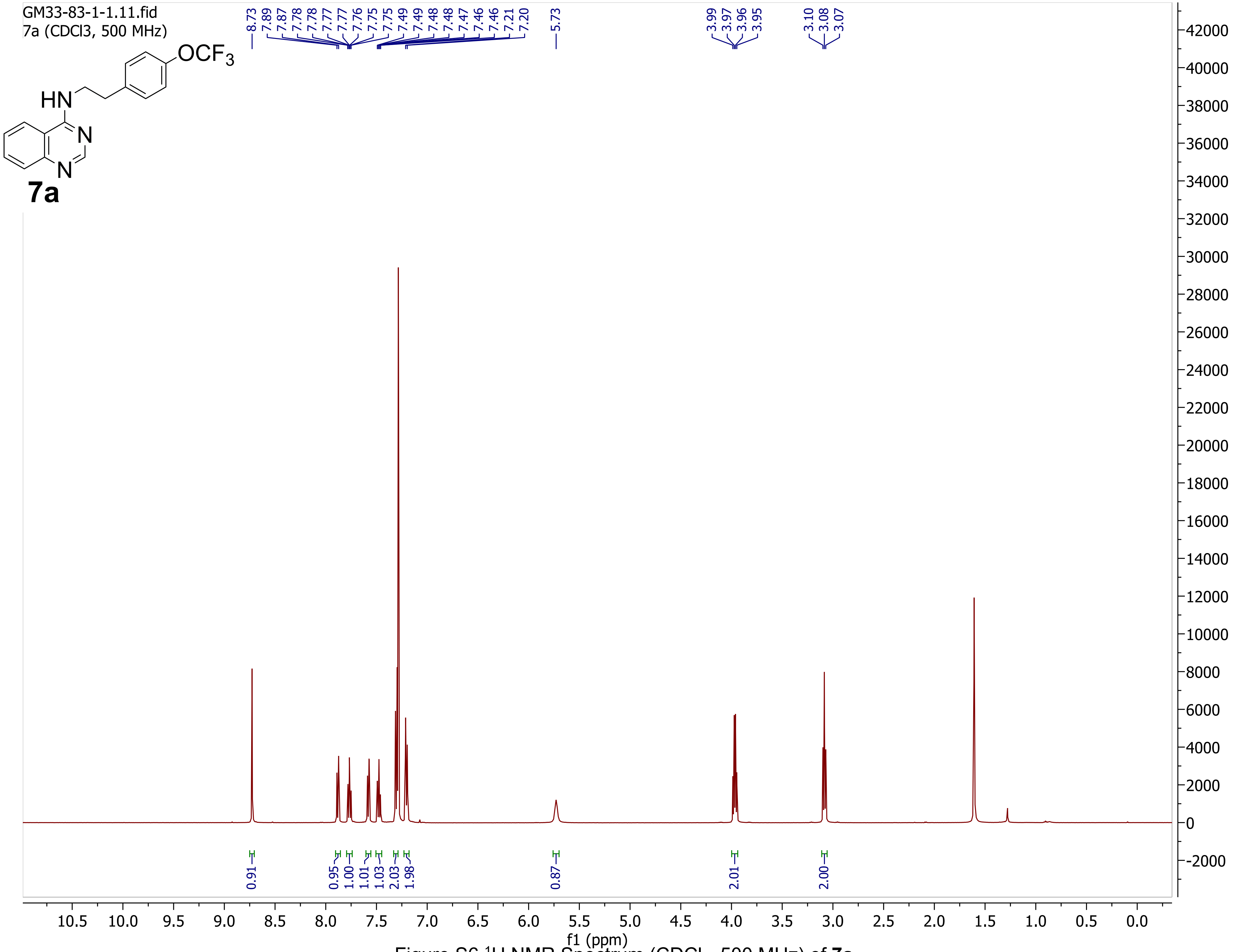

Figure S6 ^{1}H NMR Spectrum ($CDCl_3$, 500 MHz) of **7a**

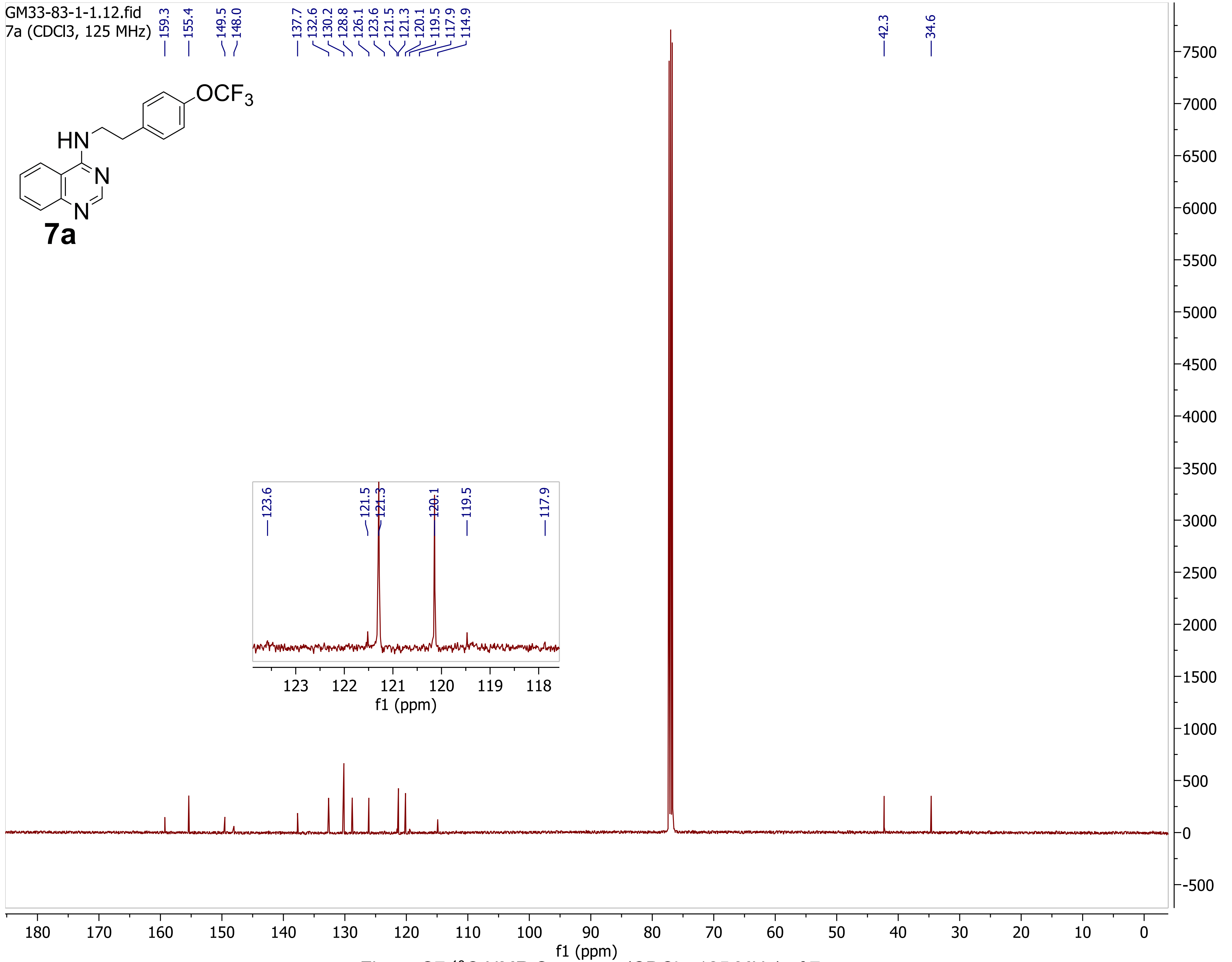

Figure S7 ^{13}C NMR Spectrum ($CDCl_3$, 125 MHz) of **7a**

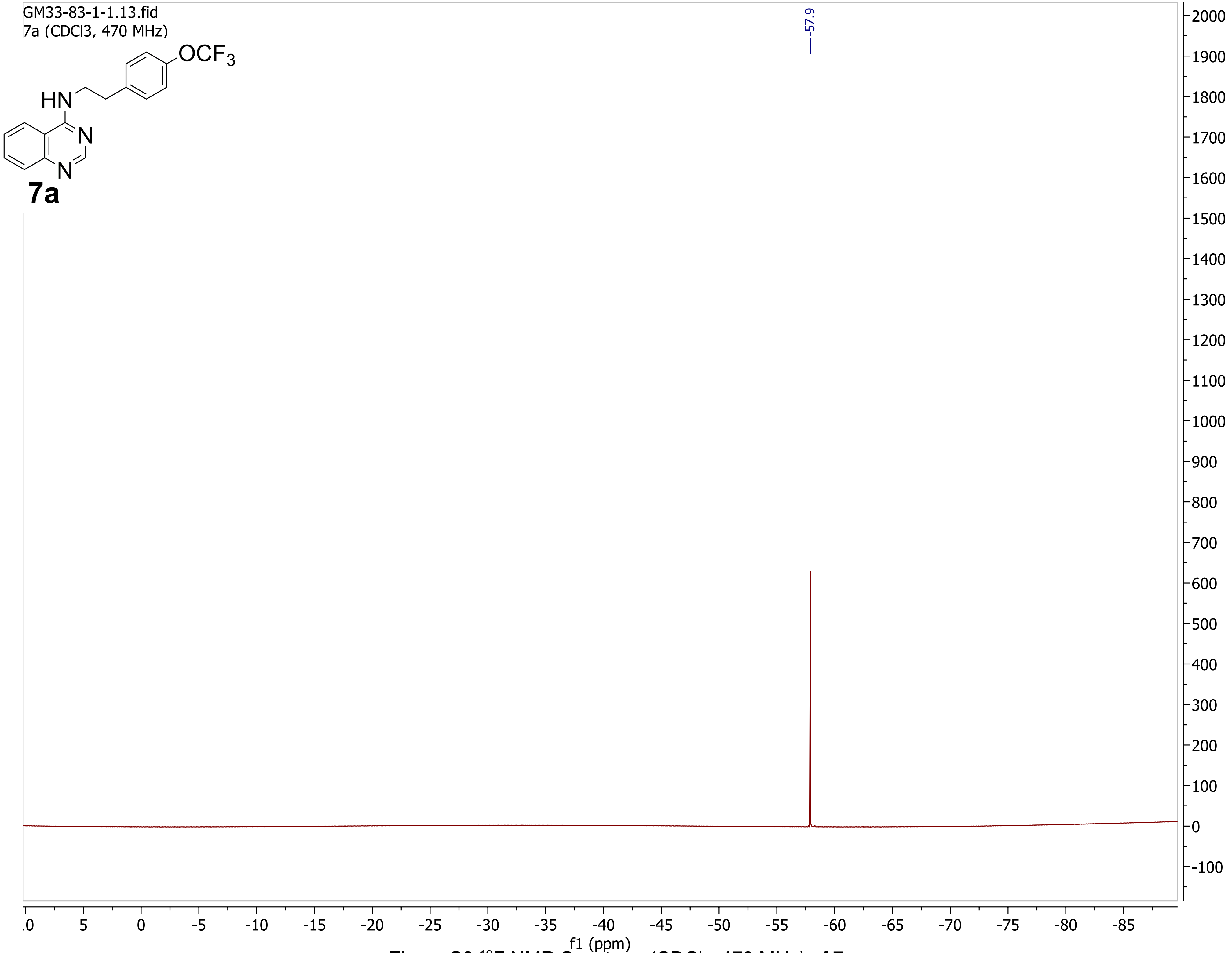

Figure S8 ^{19}F NMR Spectrum ($CDCl_3$, 470 MHz) of **7a**

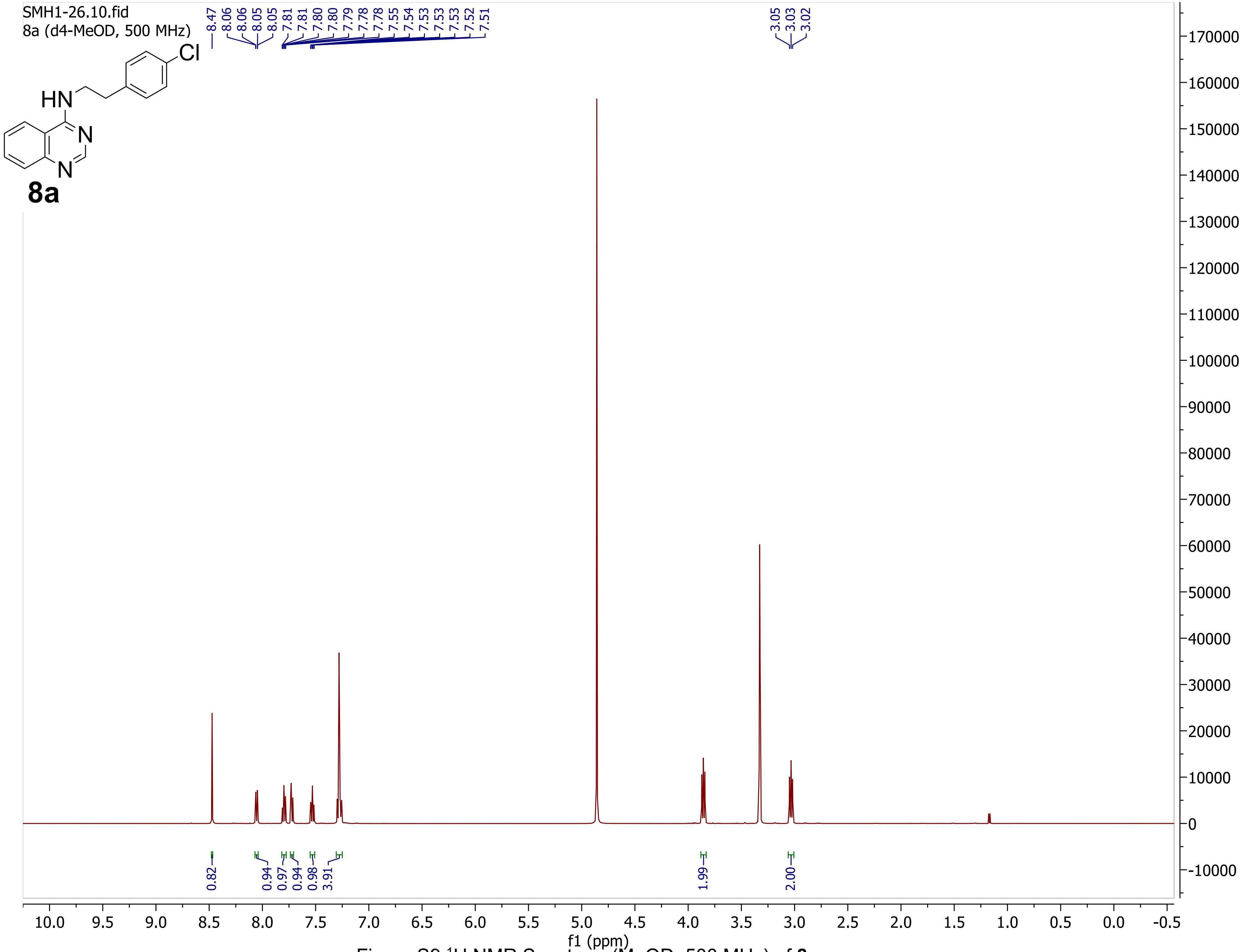

Figure S9 ^{1}H NMR Spectrum (MeOD, 500 MHz) of **8a**

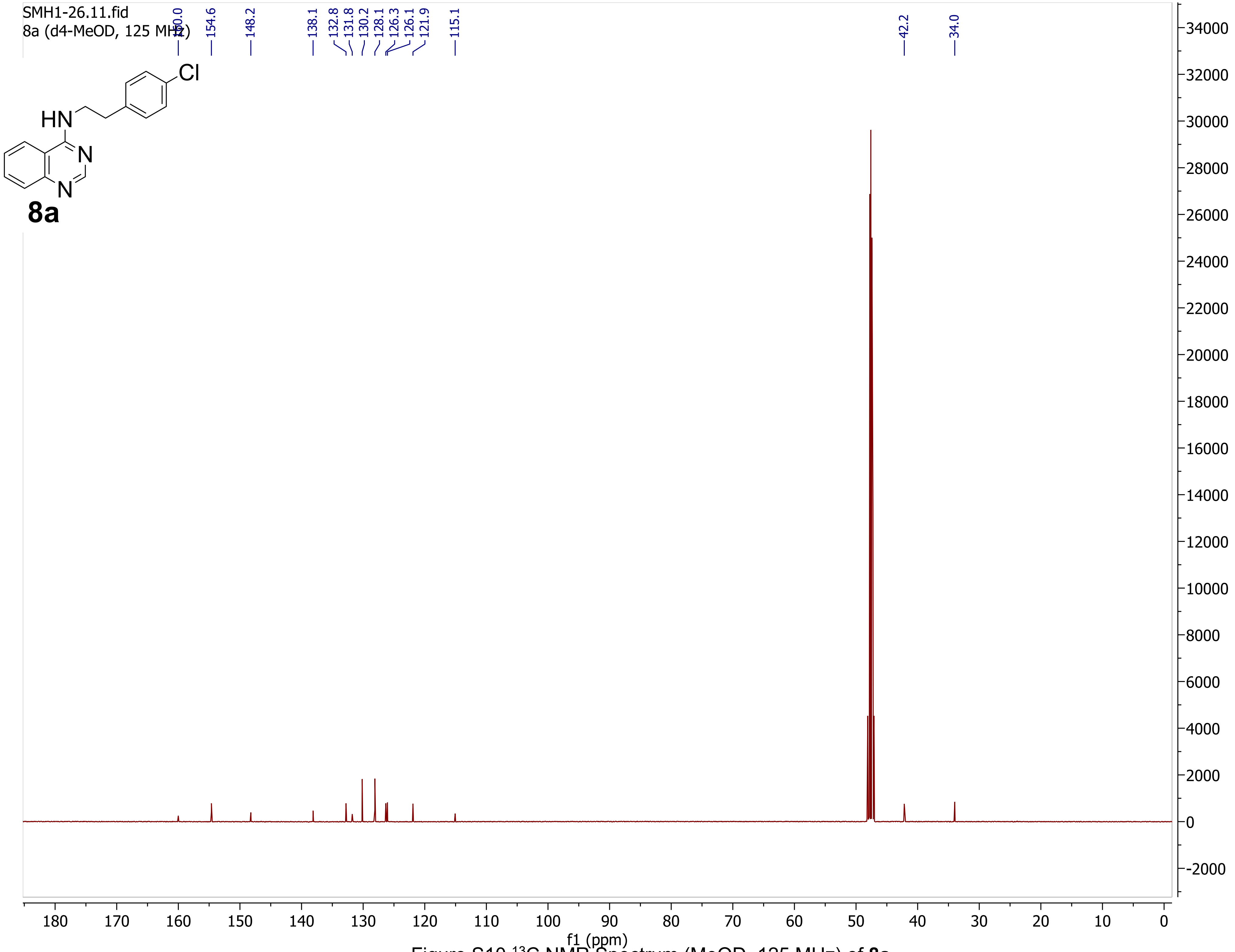

Figure S10 ^{13}C NMR Spectrum (MeOD, 125 MHz) of **8a**

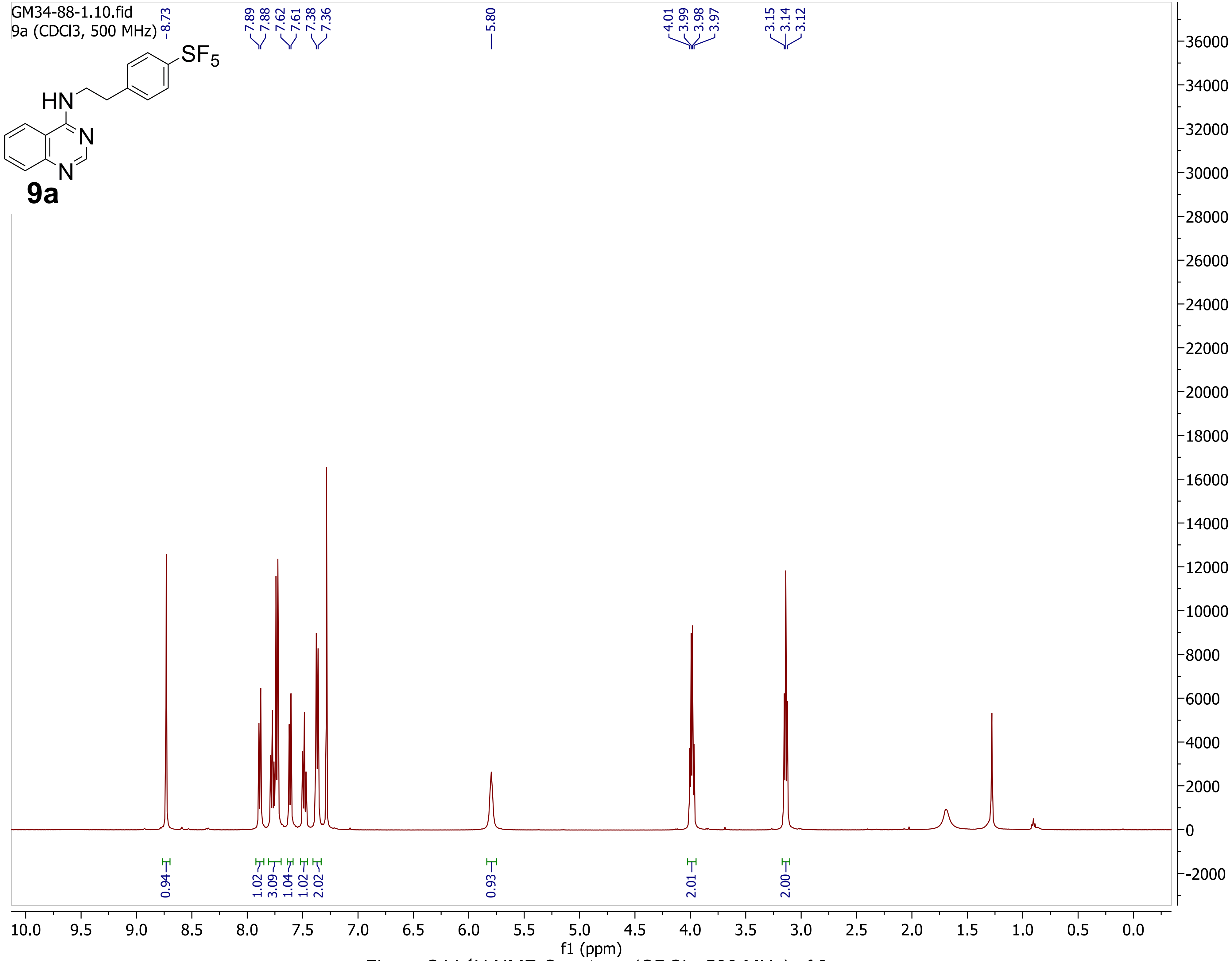

Figure S11 ^{1}H NMR Spectrum ($CDCl_3$, 500 MHz) of **9a**

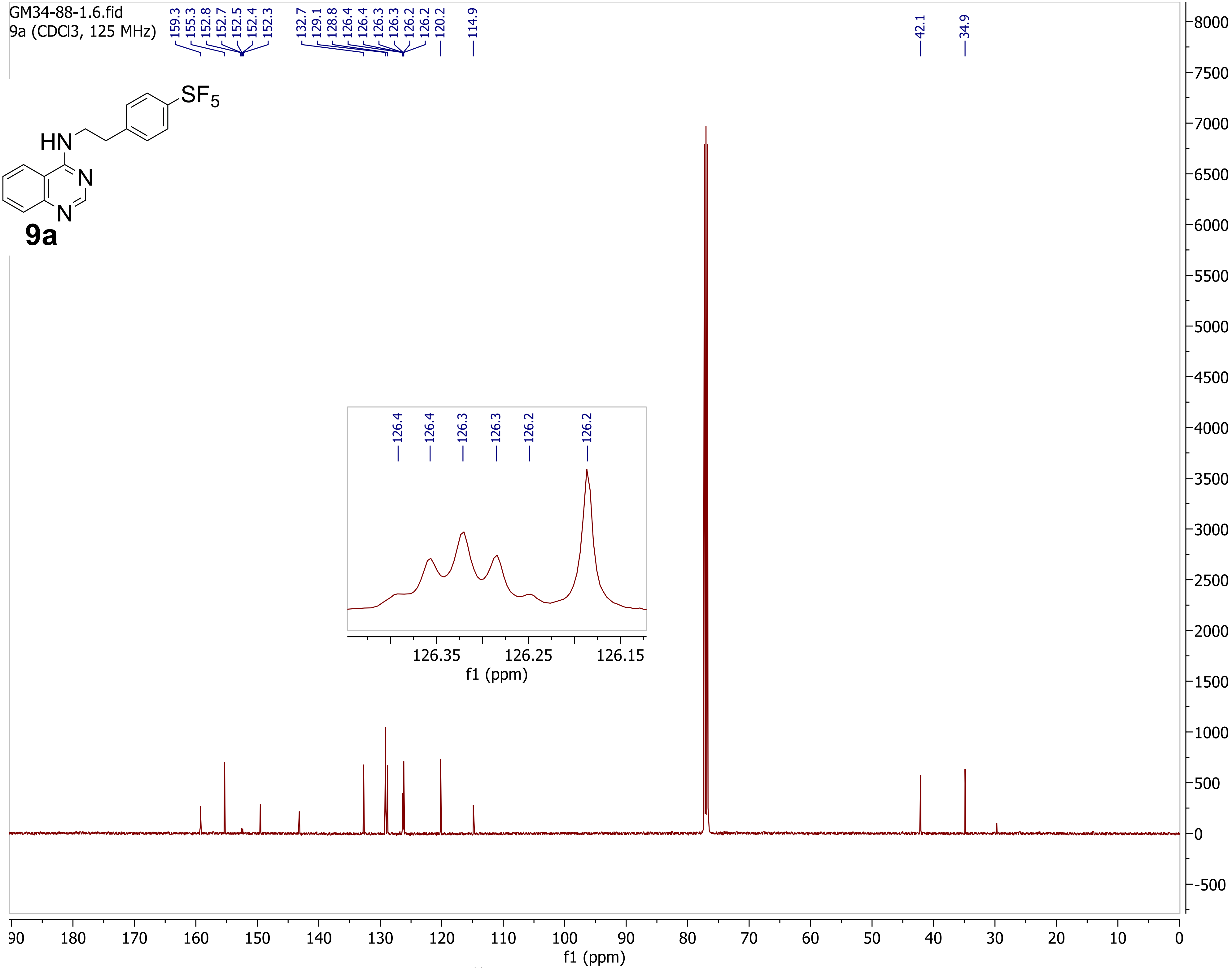

Figure S12 ^{13}C NMR Spectrum ($CDCl_3$, 125 MHz) of **9a**

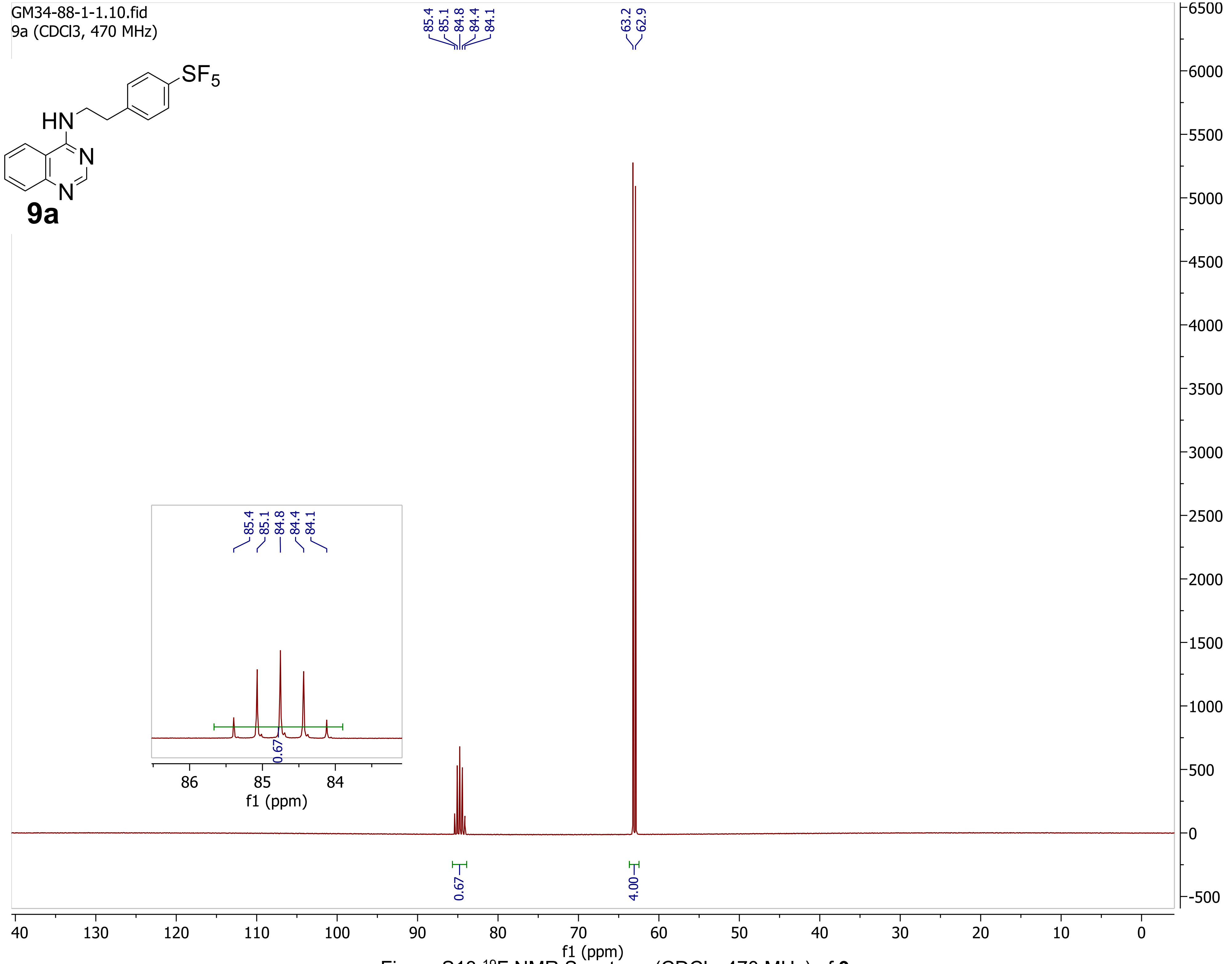

Figure S13 ^{19}F NMR Spectrum ($CDCl_3$, 470 MHz) of **9a**

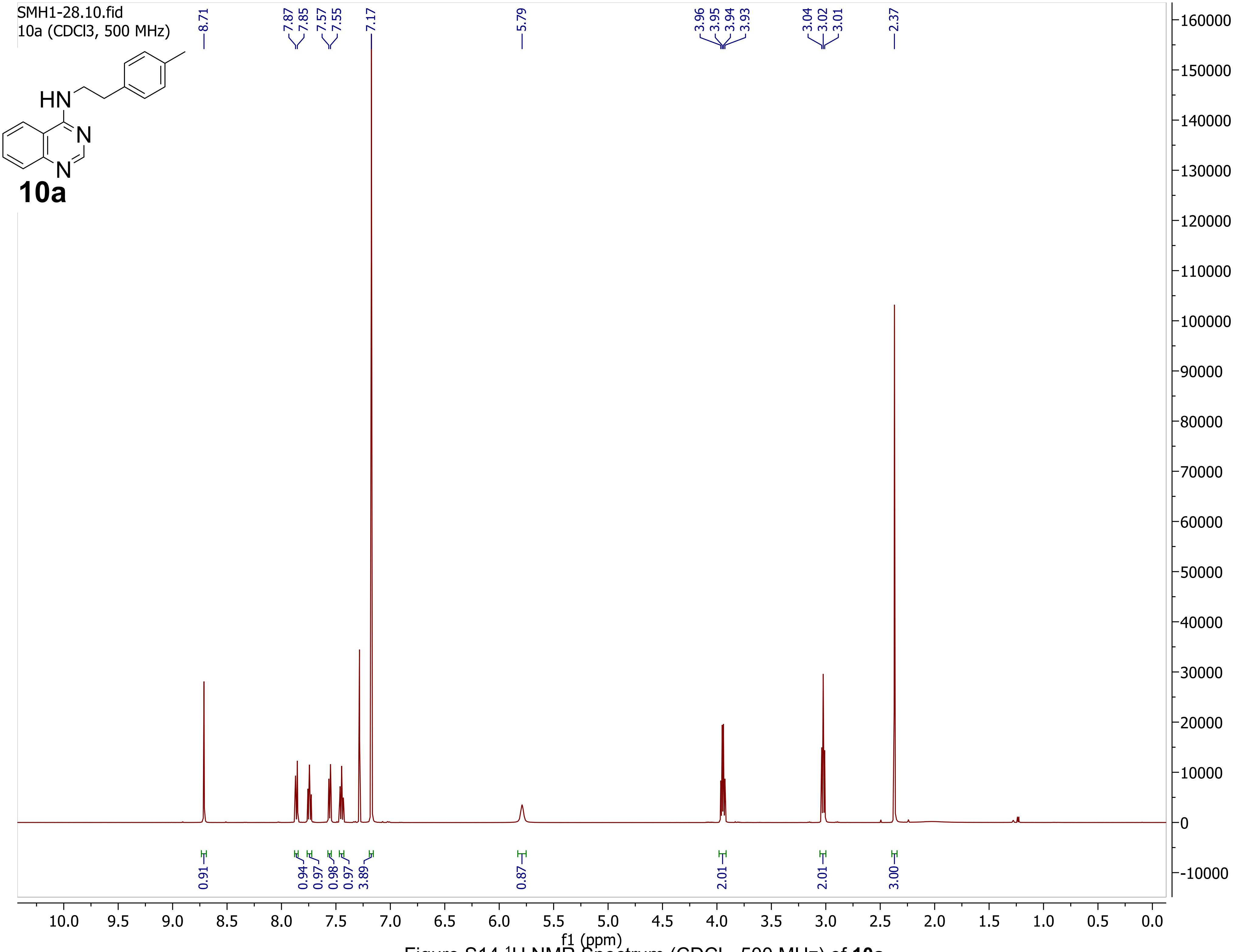

Figure S14 ^{1}H NMR Spectrum ($CDCl_3$, 500 MHz) of **10a**

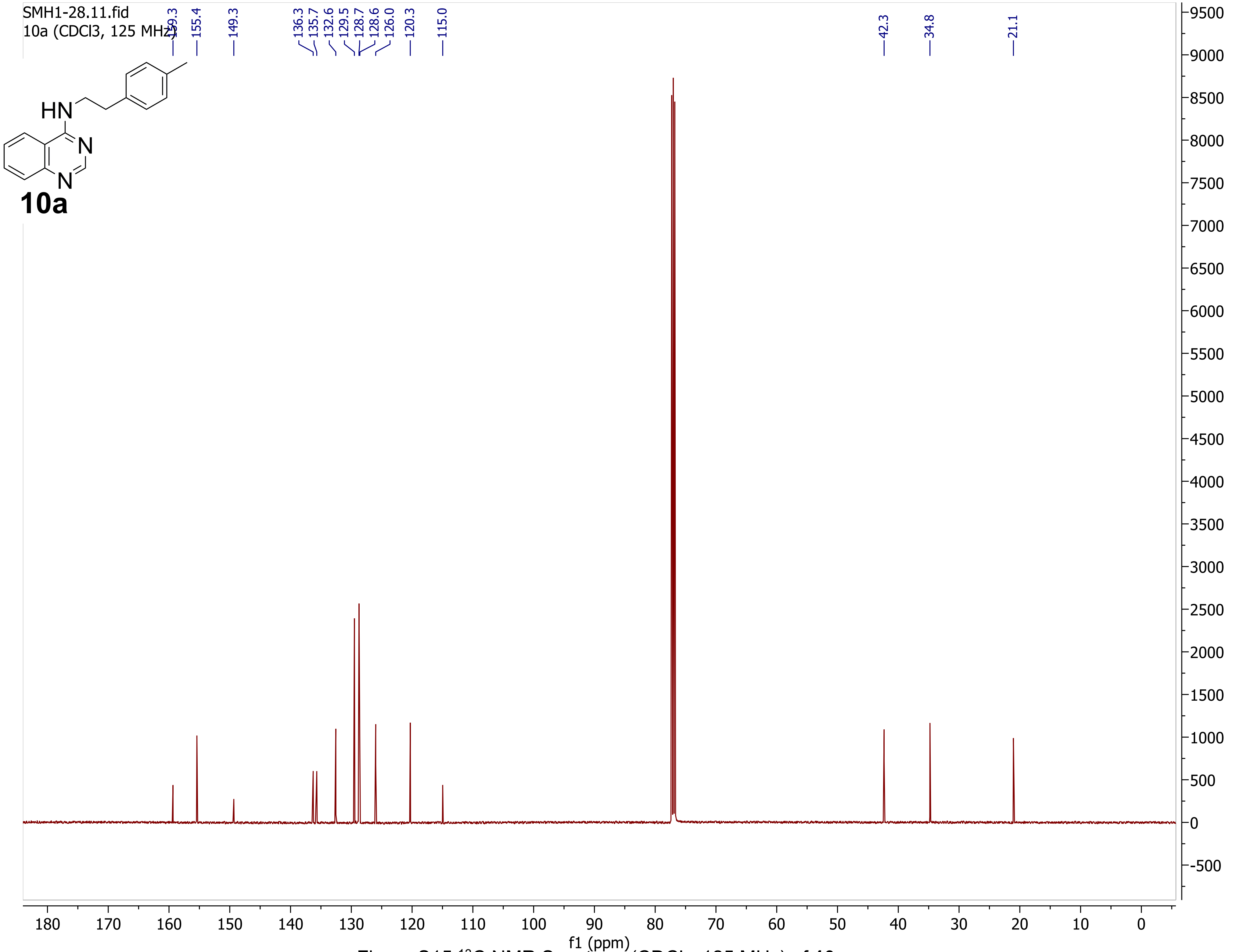

Figure S15 ^{13}C NMR Spectrum ($CDCl_3$, 125 MHz) of **10a**

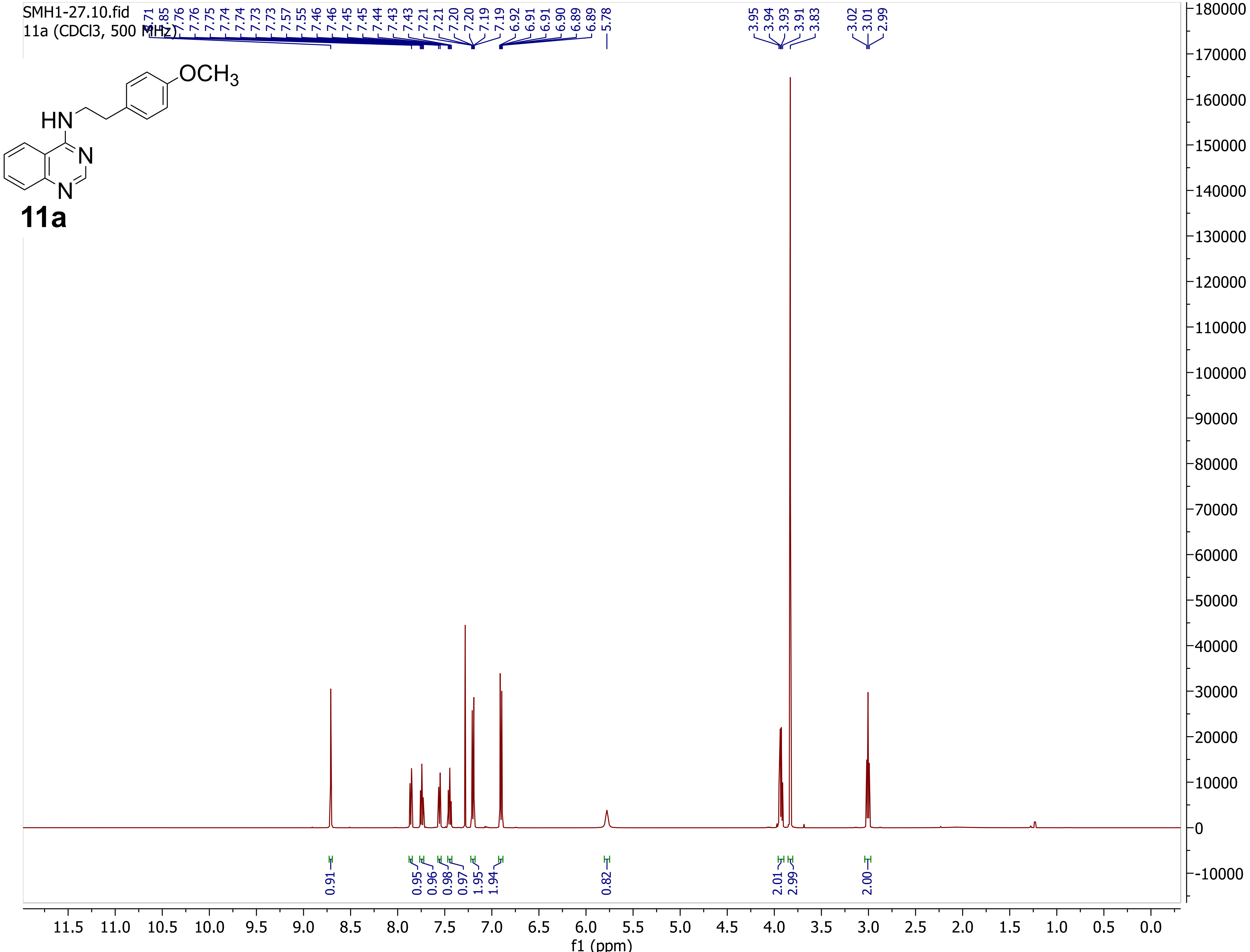

Figure S16 ^{1}H NMR Spectrum ($CDCl_3$, 500 MHz) of **11a**

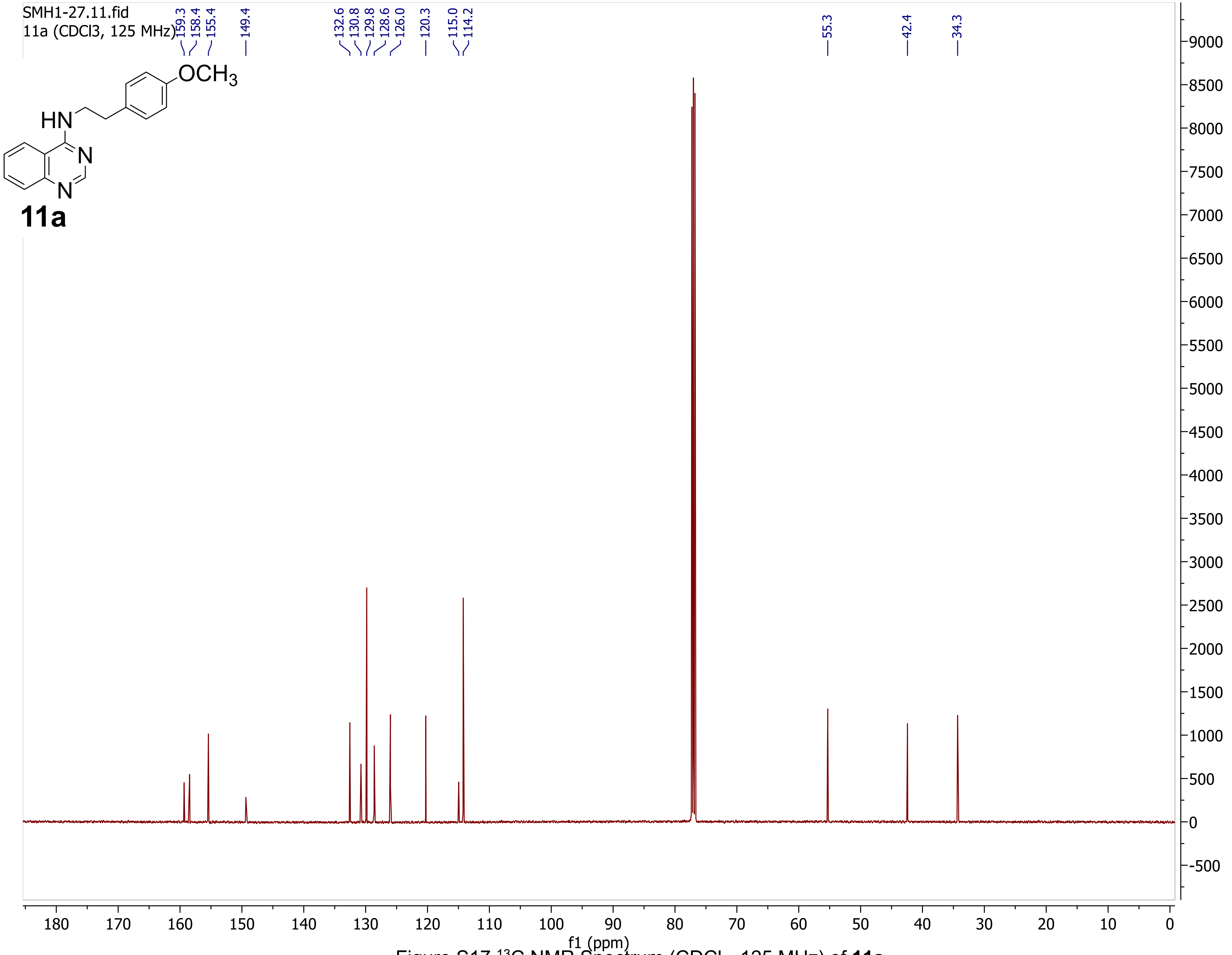

Figure S17 ^{13}C NMR Spectrum ($CDCl_3$, 125 MHz) of **11a**

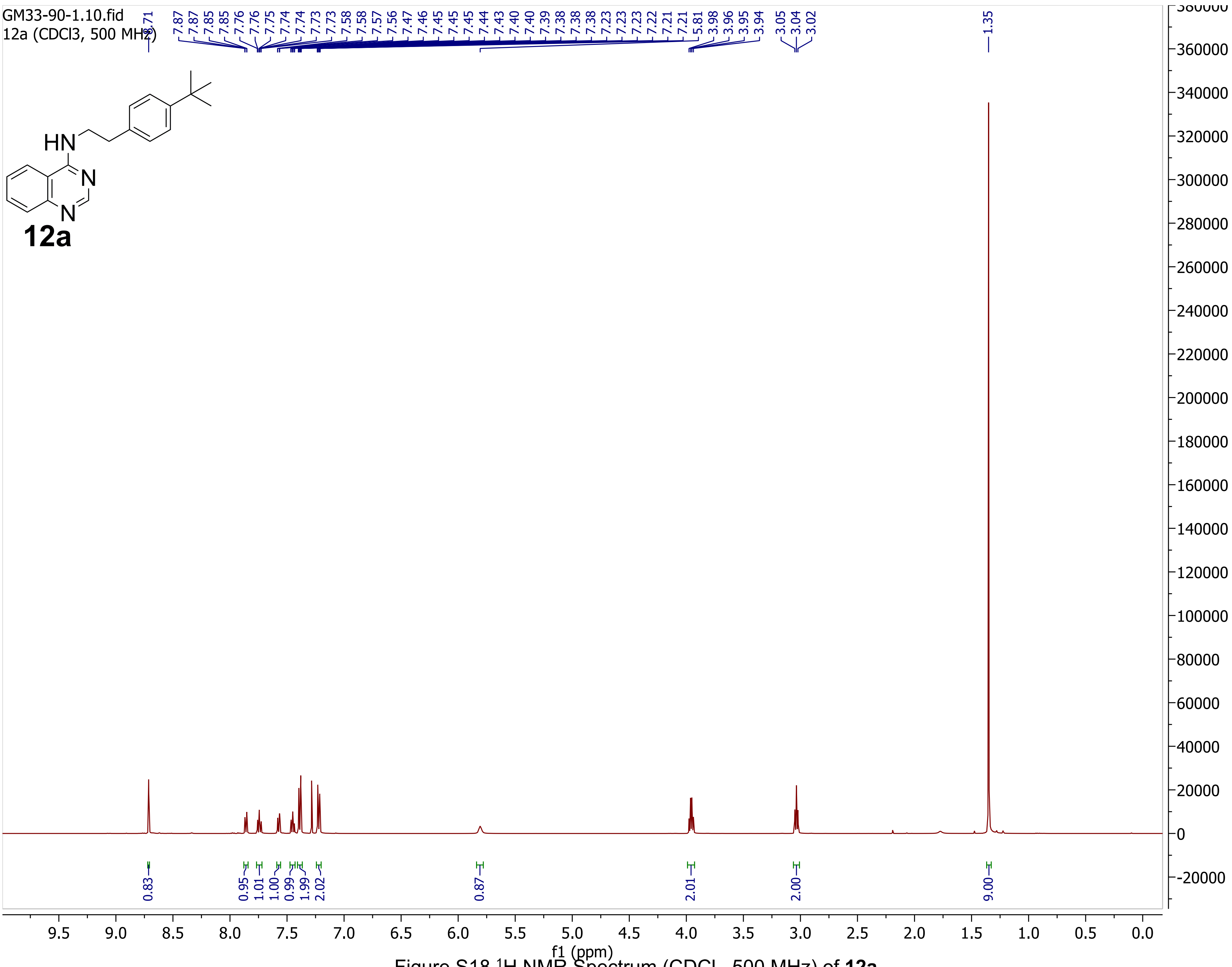

Figure S18 ^{1}H NMR Spectrum ($CDCl_3$, 500 MHz) of **12a**

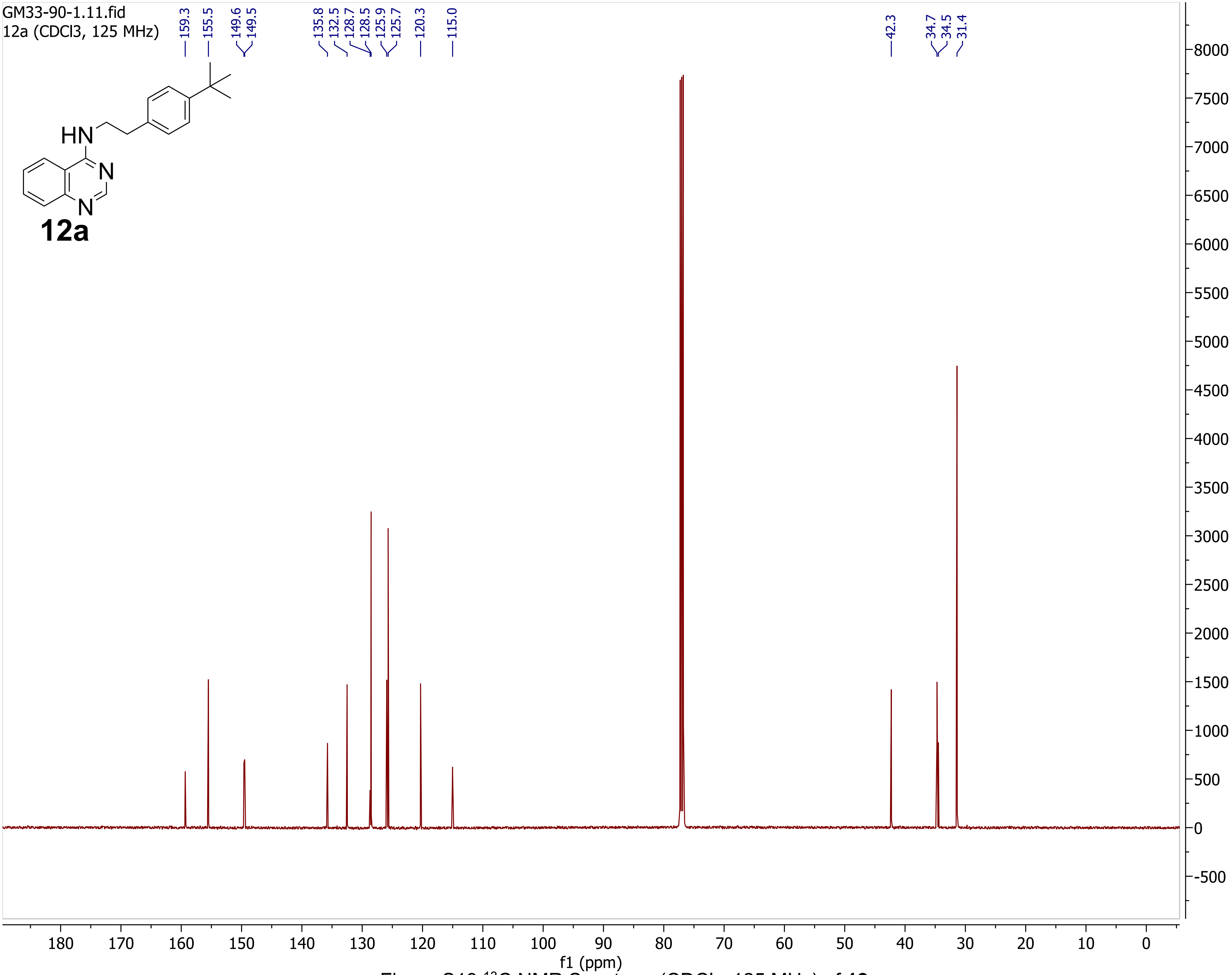

Figure S19 ^{13}C NMR Spectrum ($CDCl_3$, 125 MHz) of **12a**

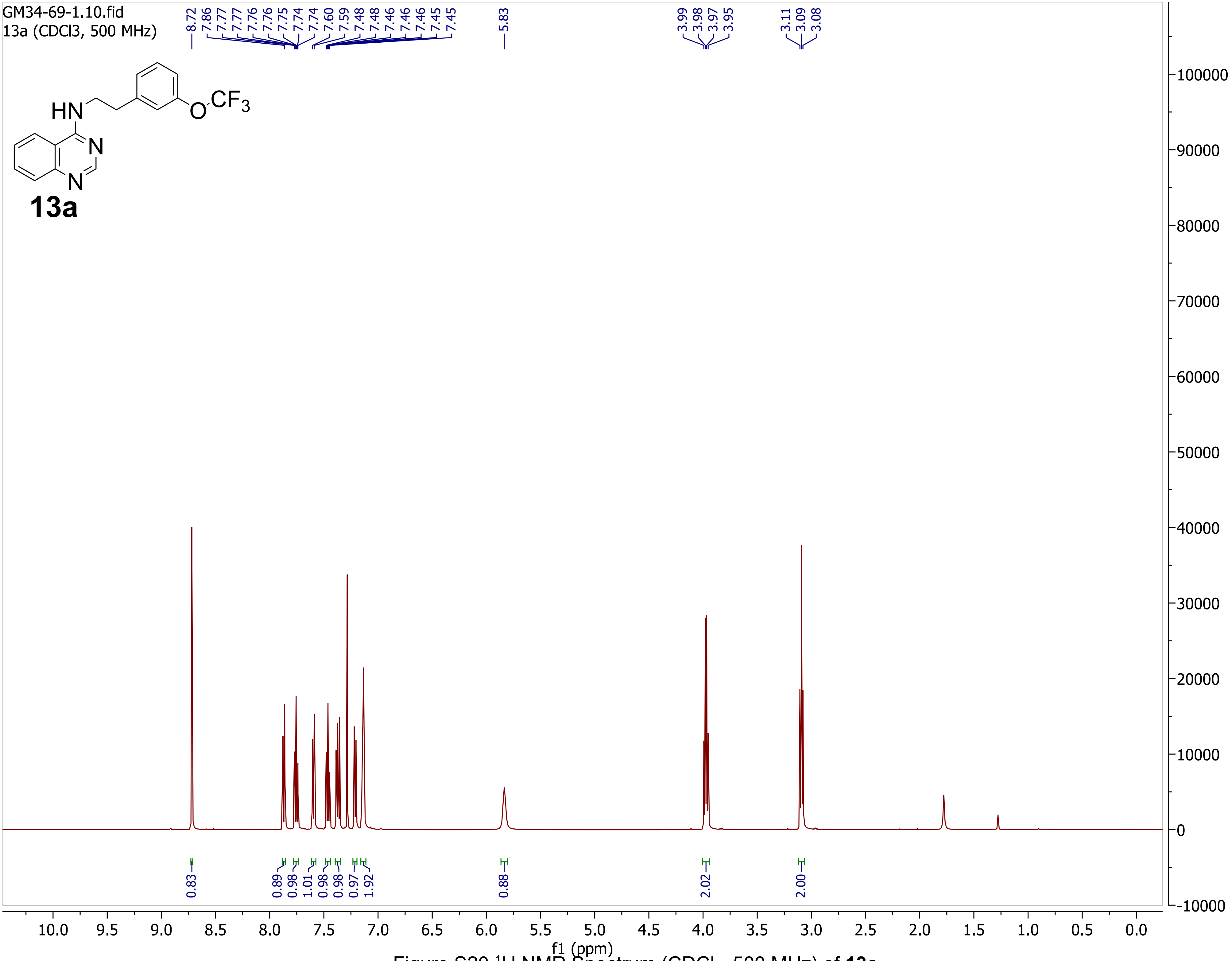

Figure S20 ^{1}H NMR Spectrum ($CDCl_3$, 500 MHz) of **13a**

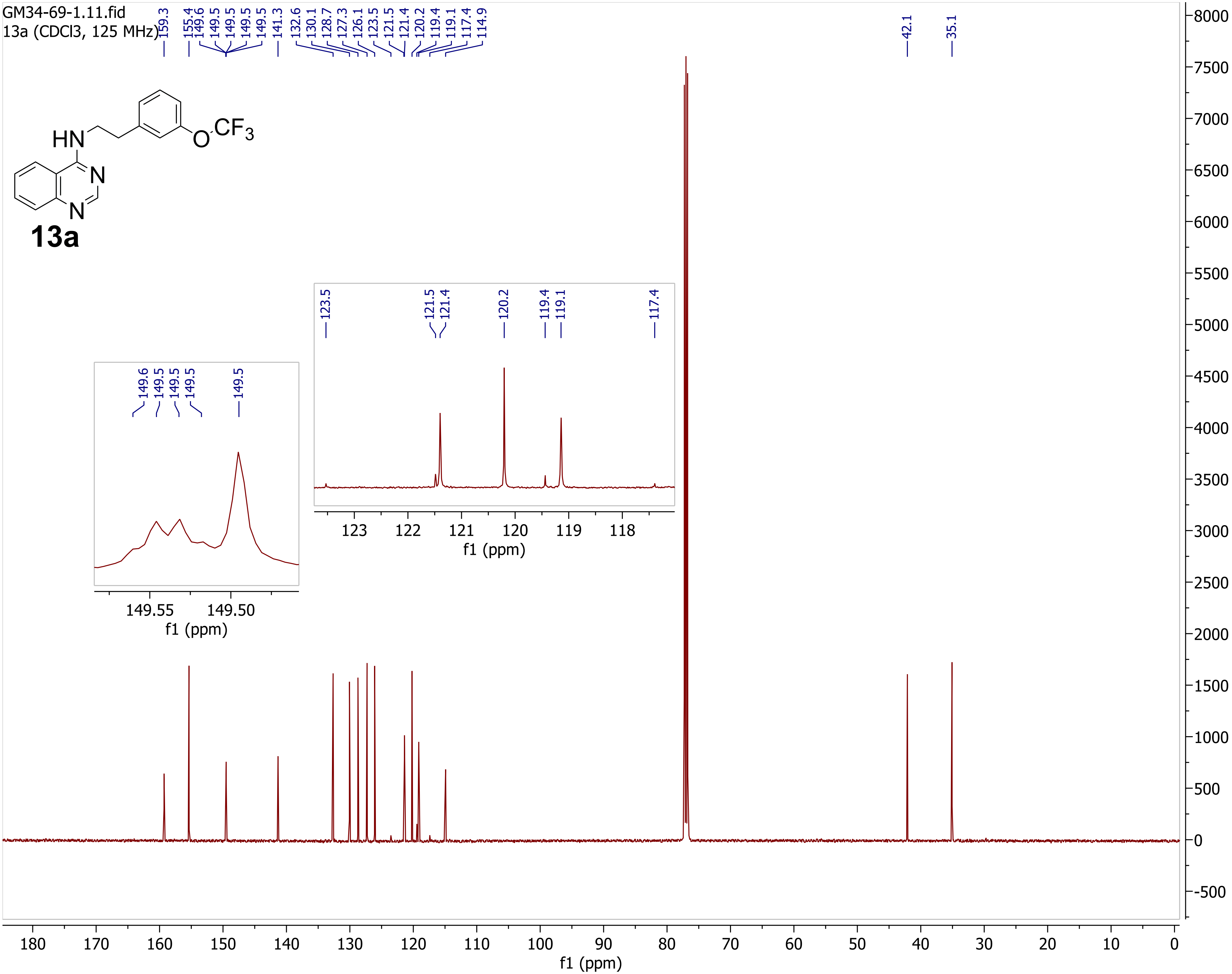

Figure S21 ^{13}C NMR Spectrum ($CDCl_3$, 125 MHz) of **13a**

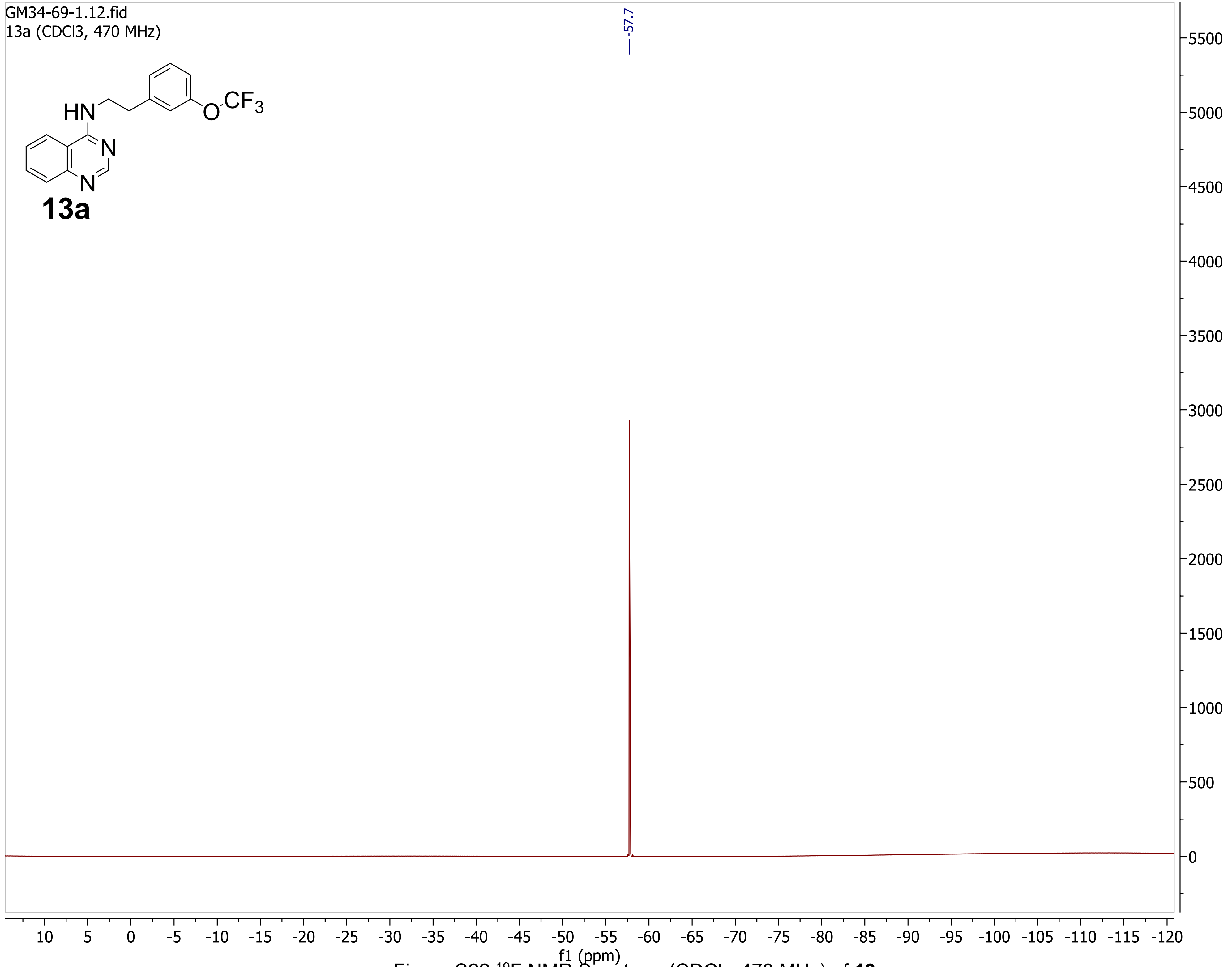

Figure S22 ^{19}F NMR Spectrum ($CDCl_3$, 470 MHz) of **13a**

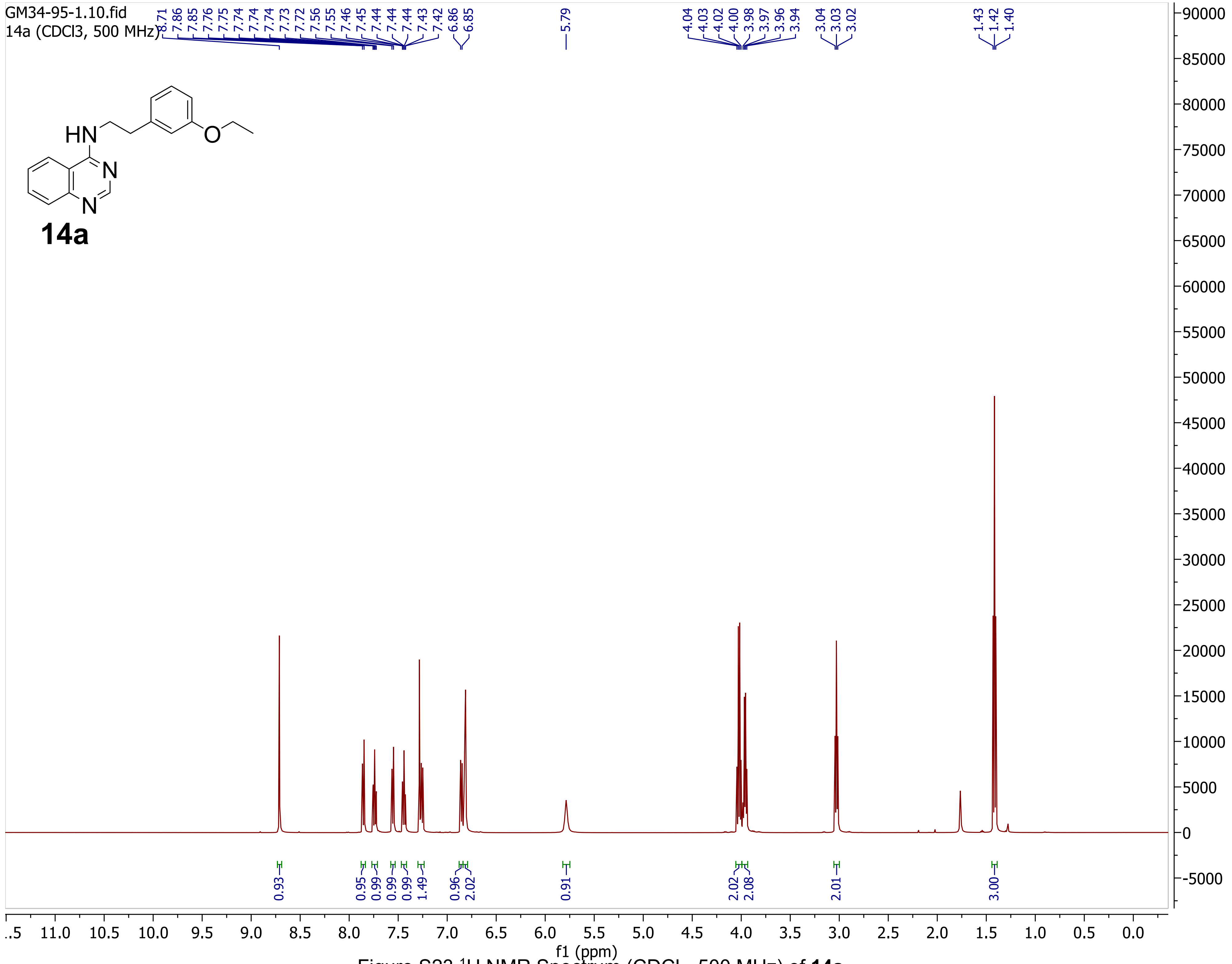

Figure S23 ^{1}H NMR Spectrum ($CDCl_3$, 500 MHz) of **14a**

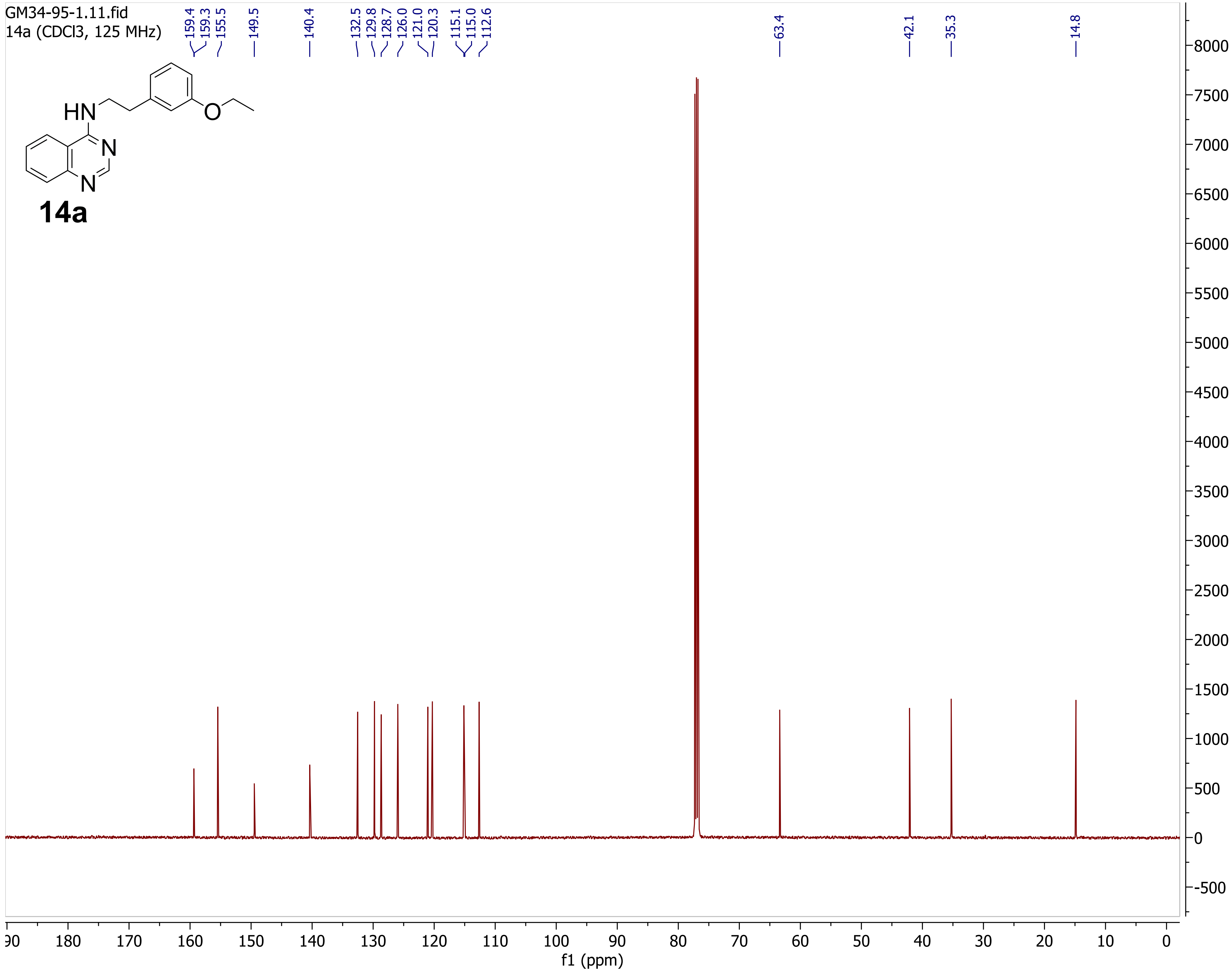

Figure S24 ^{13}C NMR Spectrum ($CDCl_3$, 125 MHz) of **14a**

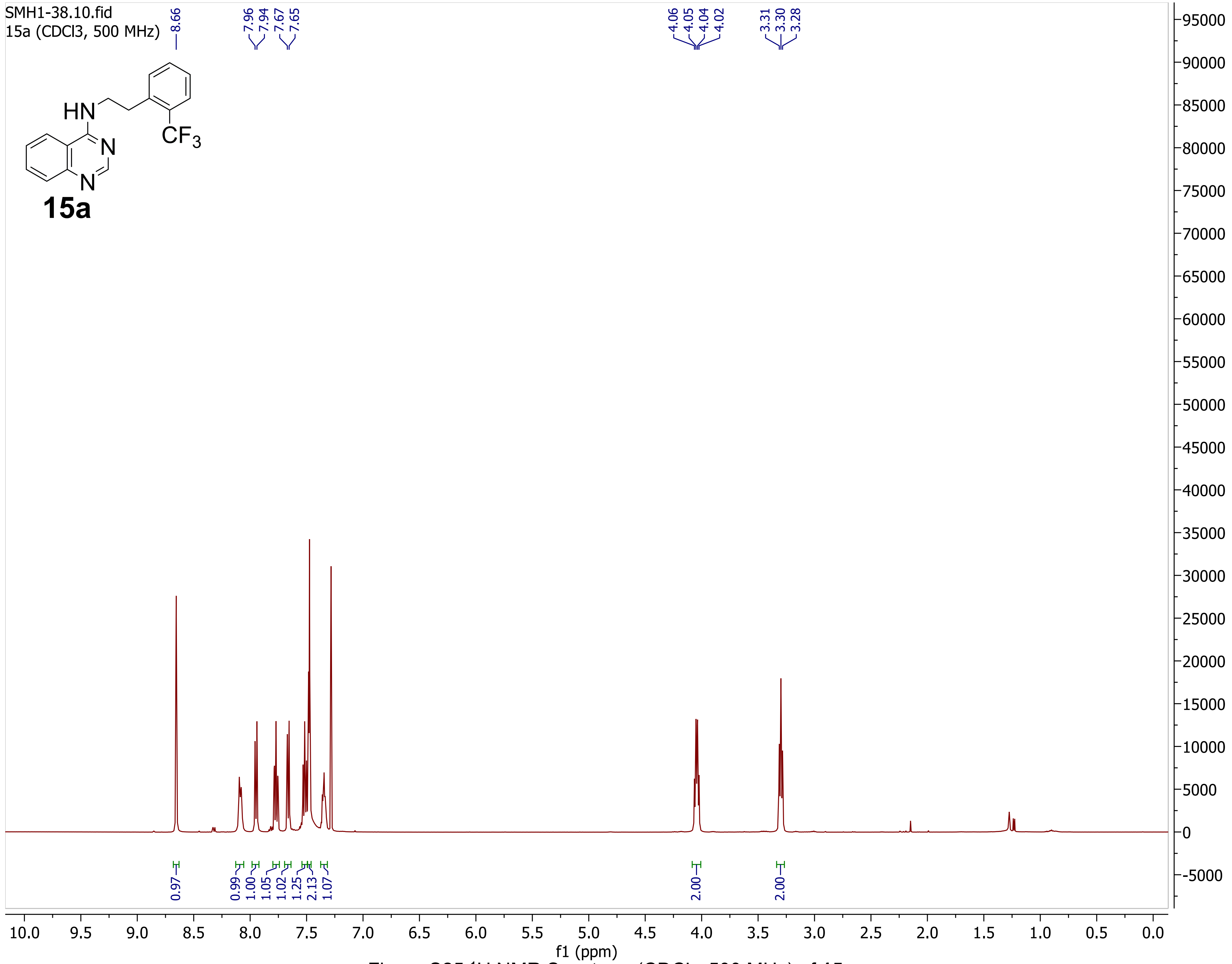

Figure S25 ^{1}H NMR Spectrum ($CDCl_3$, 500 MHz) of **15a**

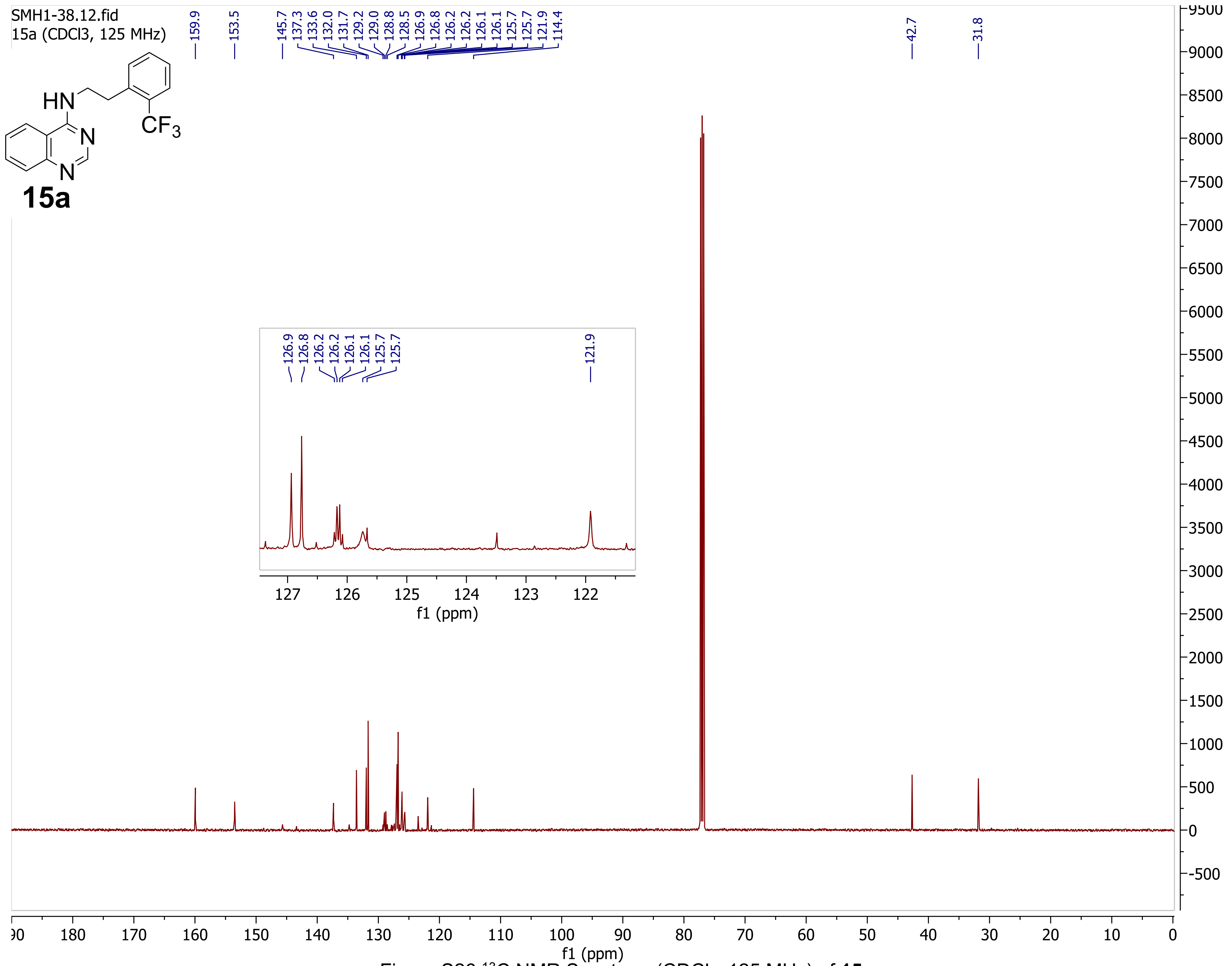

Figure S26 ^{13}C NMR Spectrum ($CDCl_3$, 125 MHz) of **15a**

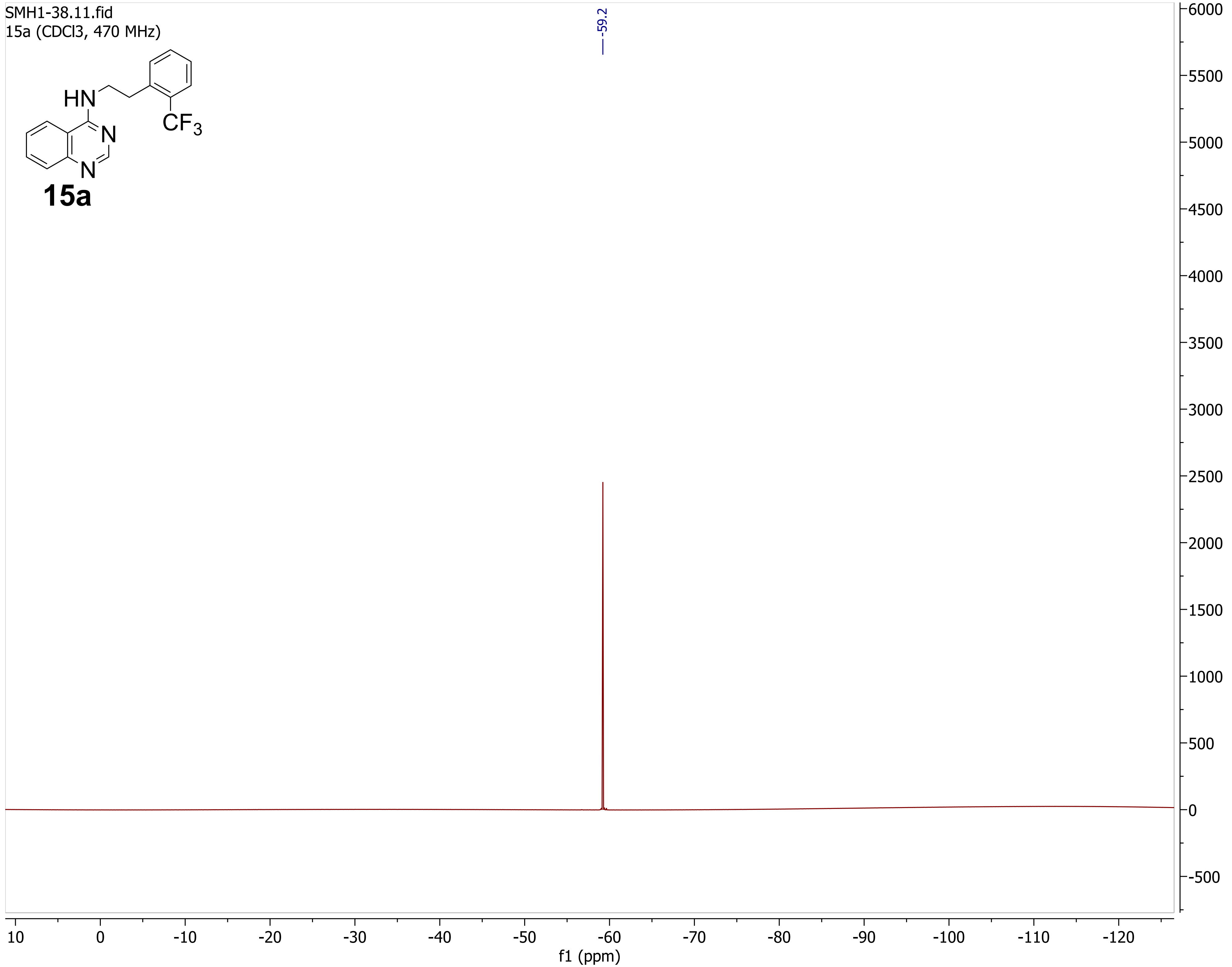

Figure S27 ^{19}F NMR Spectrum ($CDCl_3$, 470 MHz) of **15a**

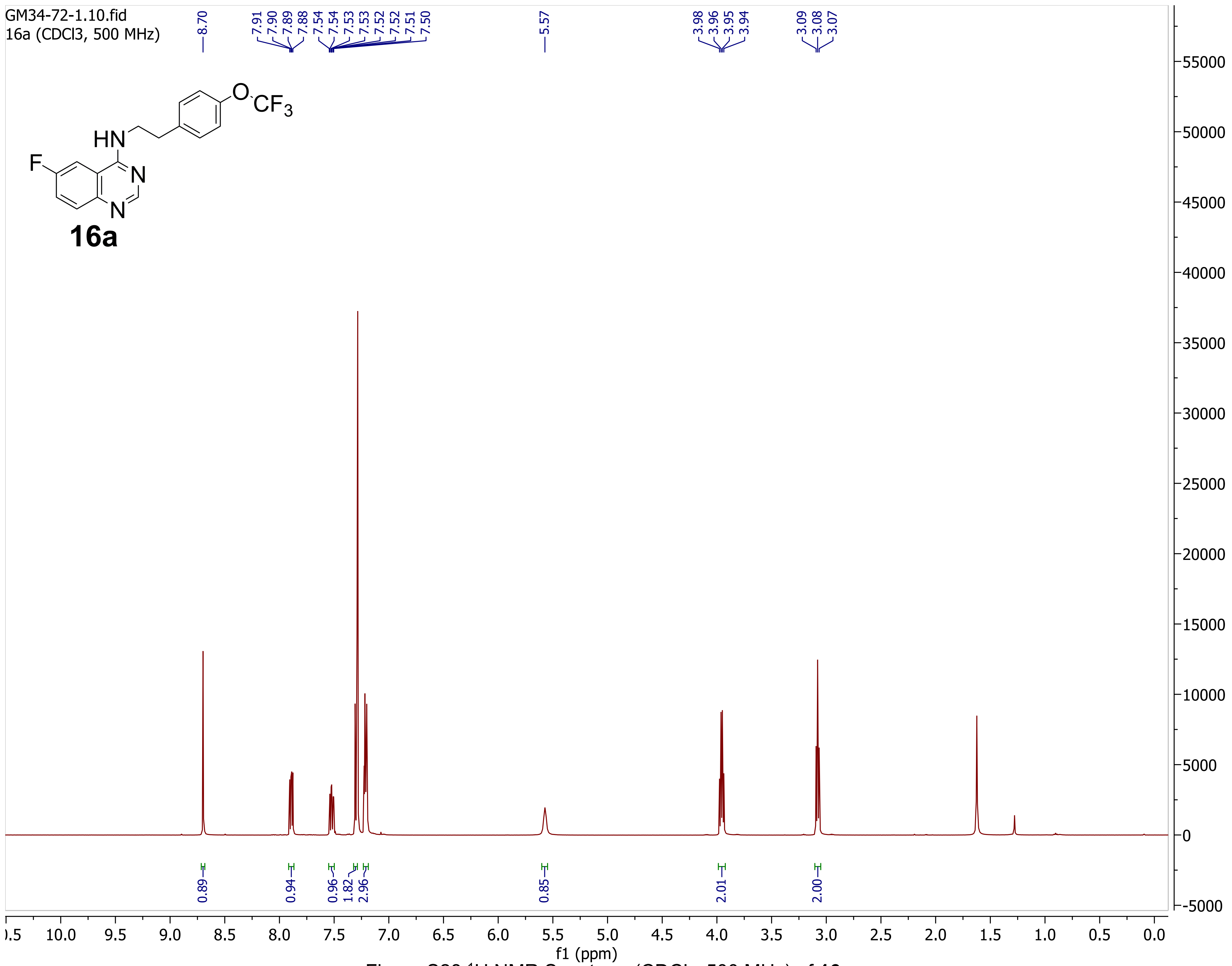

Figure S28 ^{1}H NMR Spectrum ($CDCl_3$, 500 MHz) of **16a**

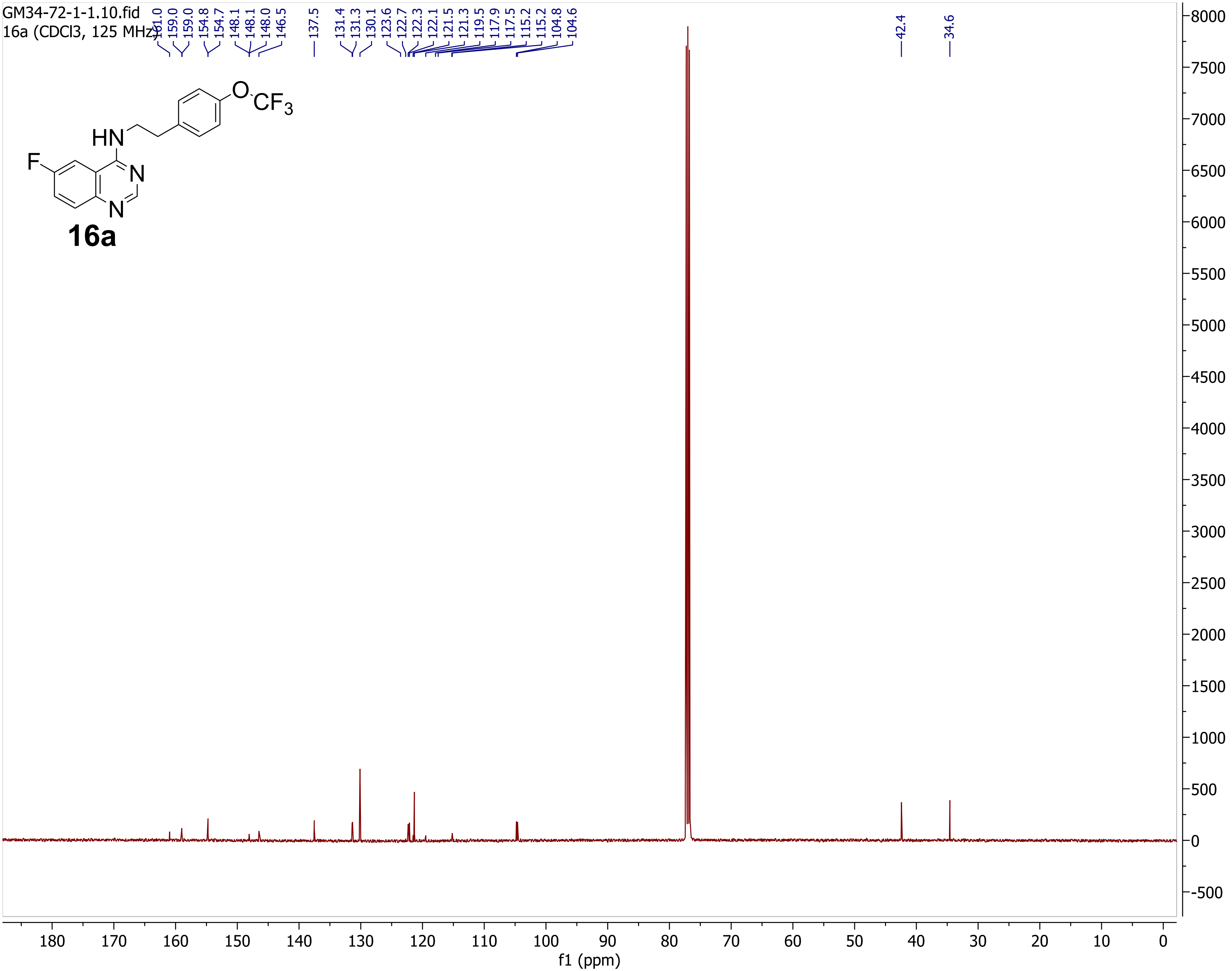

Figure S29 ^{13}C NMR Spectrum ($CDCl_3$, 125 MHz) of **16a**

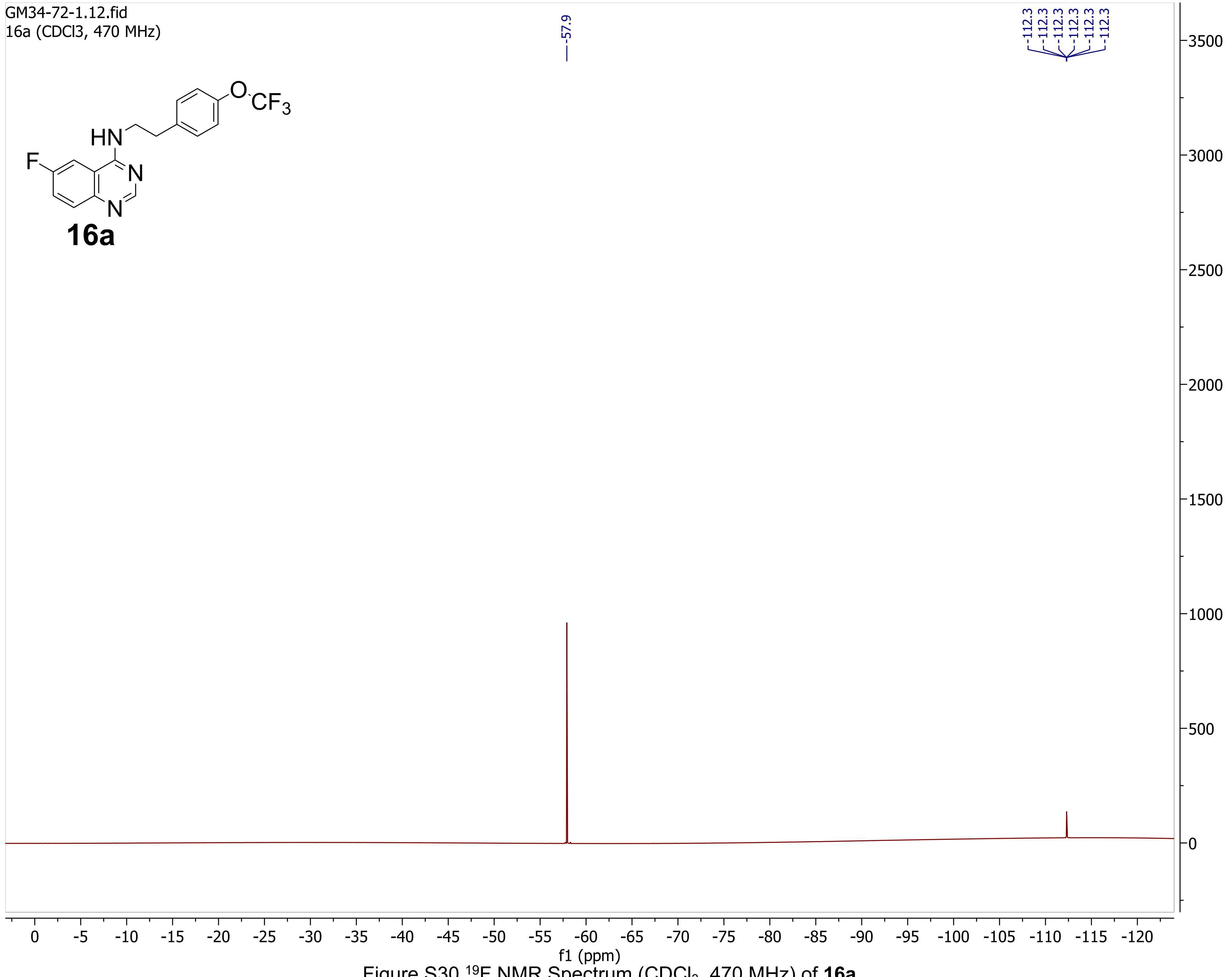

Figure S30 ^{19}F NMR Spectrum ($CDCl_3$, 470 MHz) of **16a**

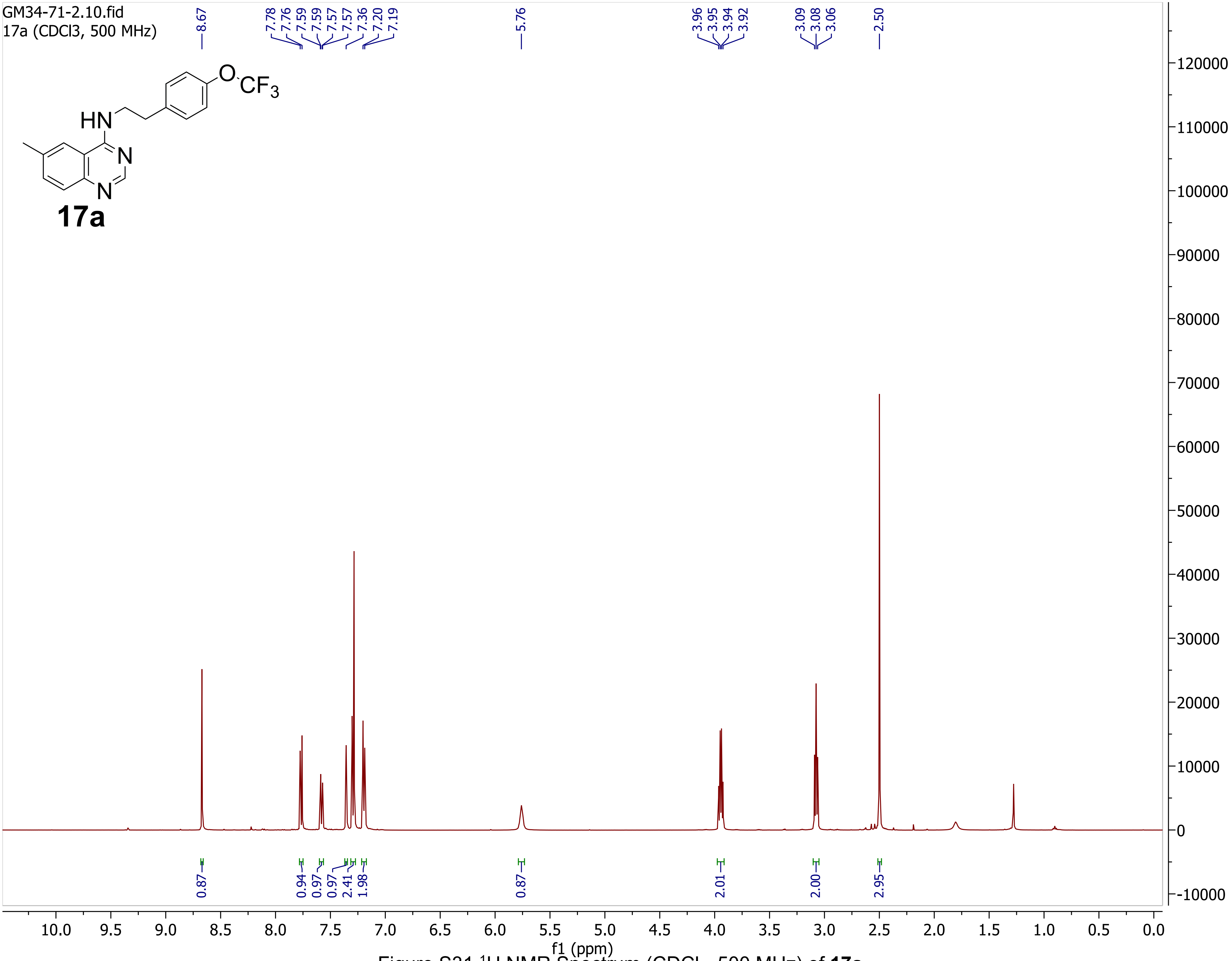

Figure S31 ^{1}H NMR Spectrum ($CDCl_3$, 500 MHz) of **17a**

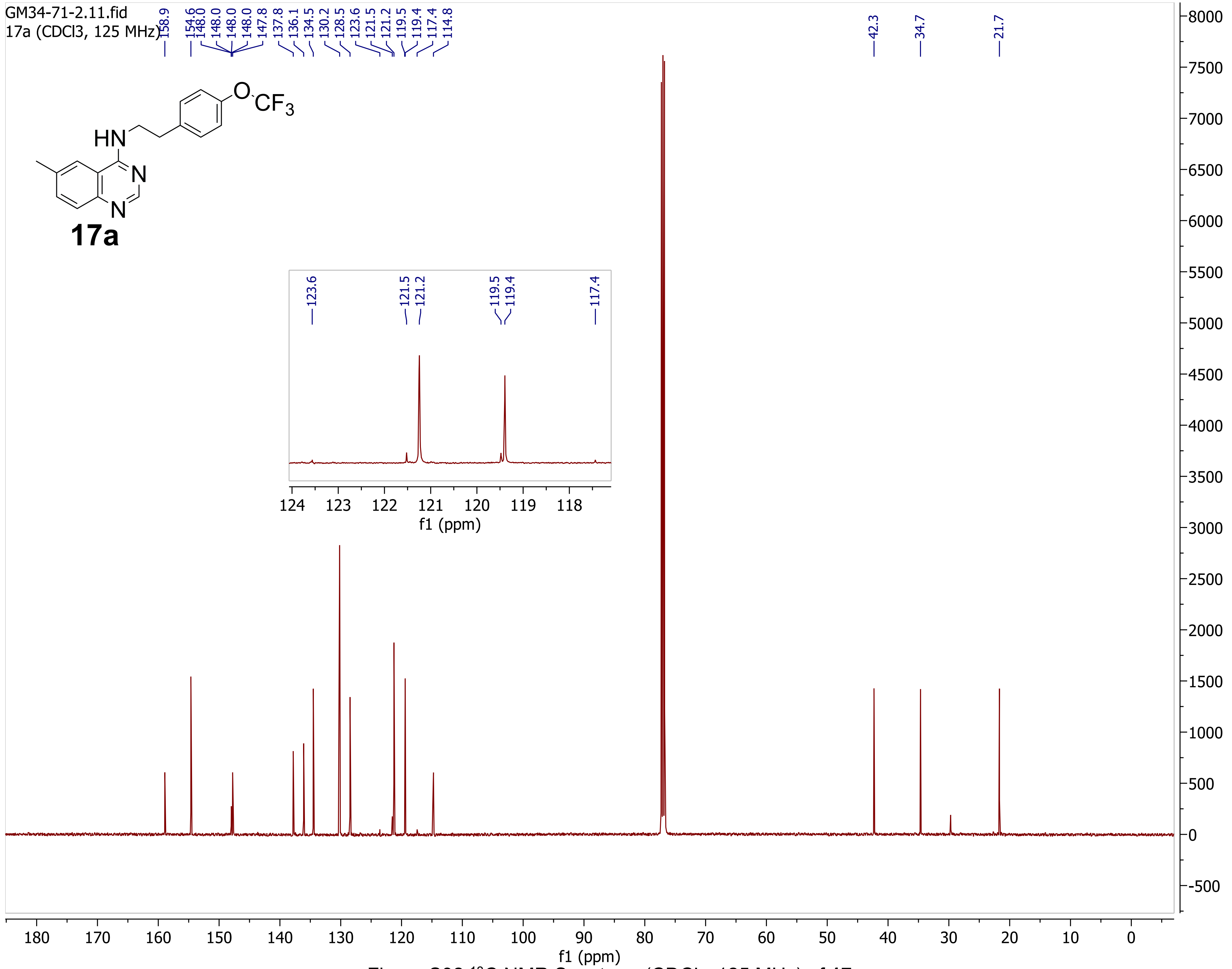

Figure S32 ^{13}C NMR Spectrum ($CDCl_3$, 125 MHz) of **17a**

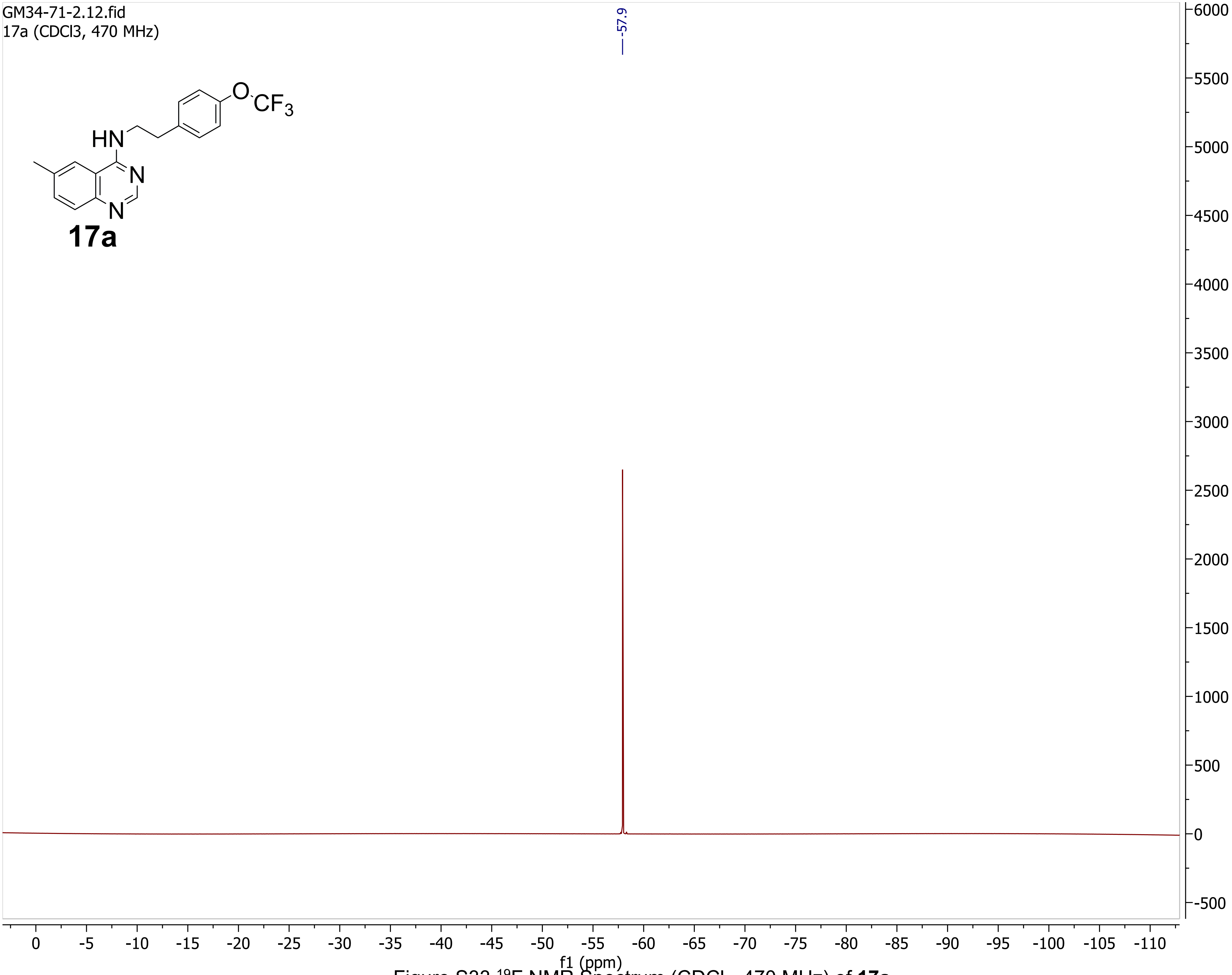

Figure S33 ^{19}F NMR Spectrum ($CDCl_3$, 470 MHz) of **17a**

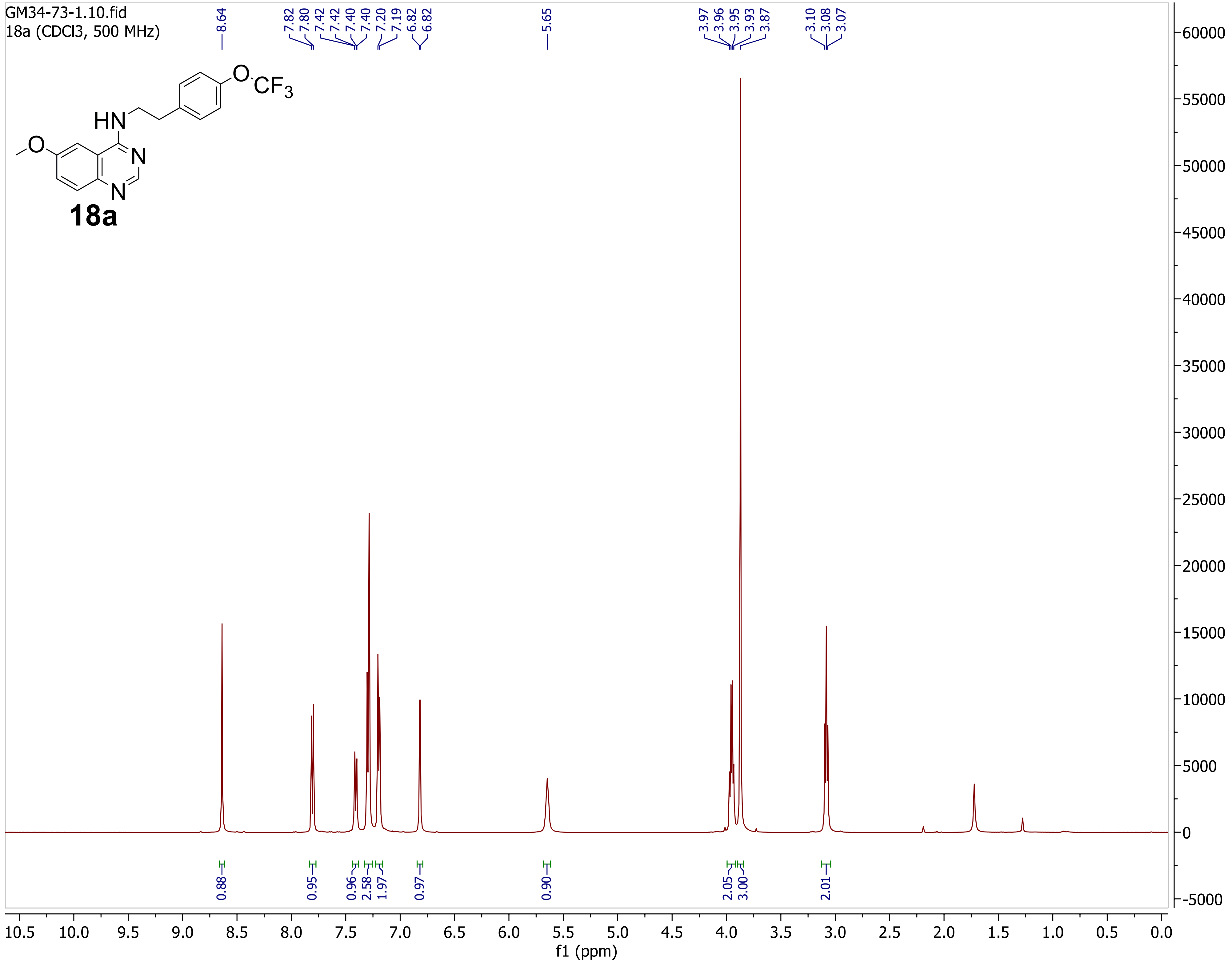

Figure S34 ^{1}H NMR Spectrum ($CDCl_3$, 500 MHz) of **18a**

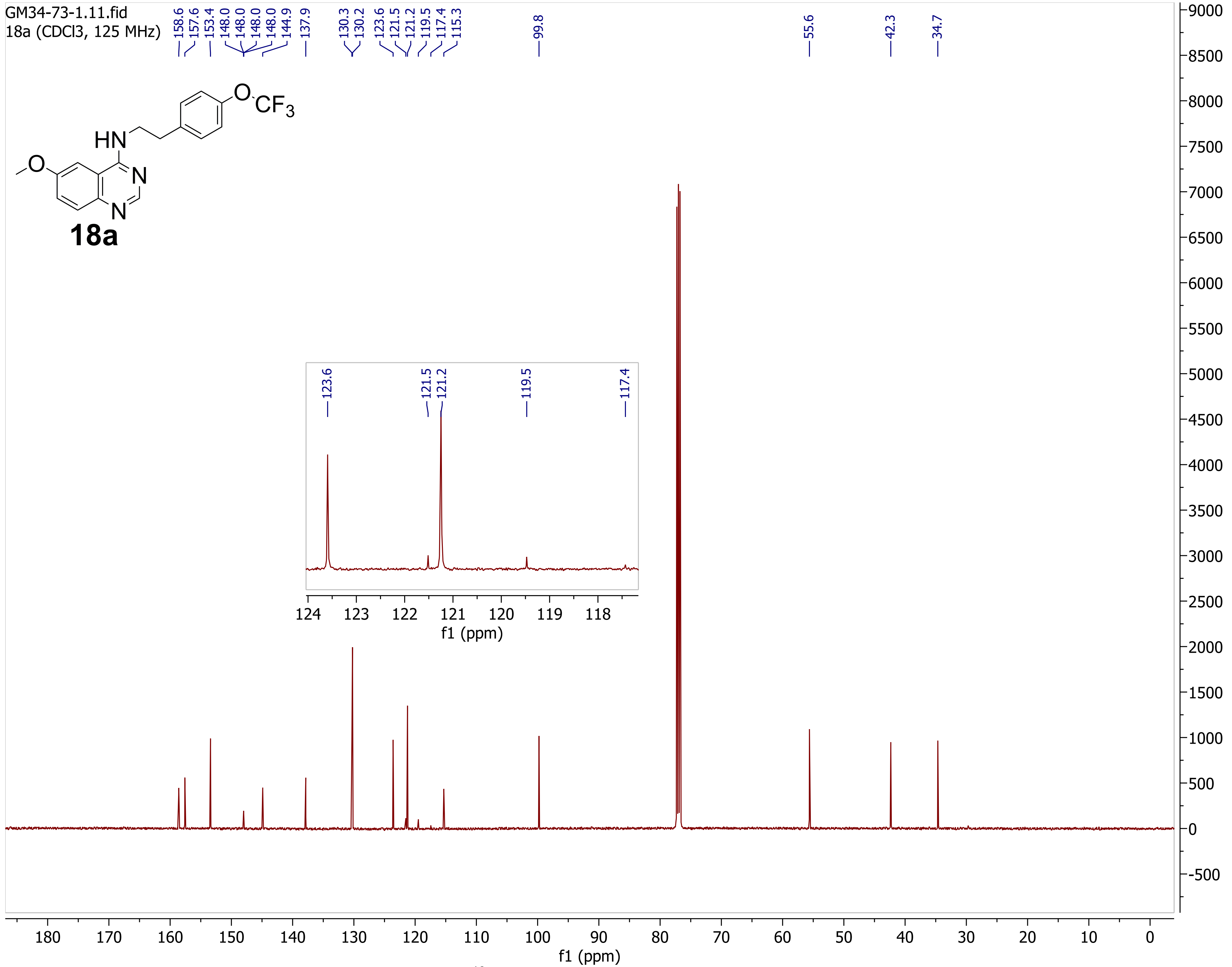

Figure S35 ^{13}C NMR Spectrum ($CDCl_3$, 125 MHz) of **18a**

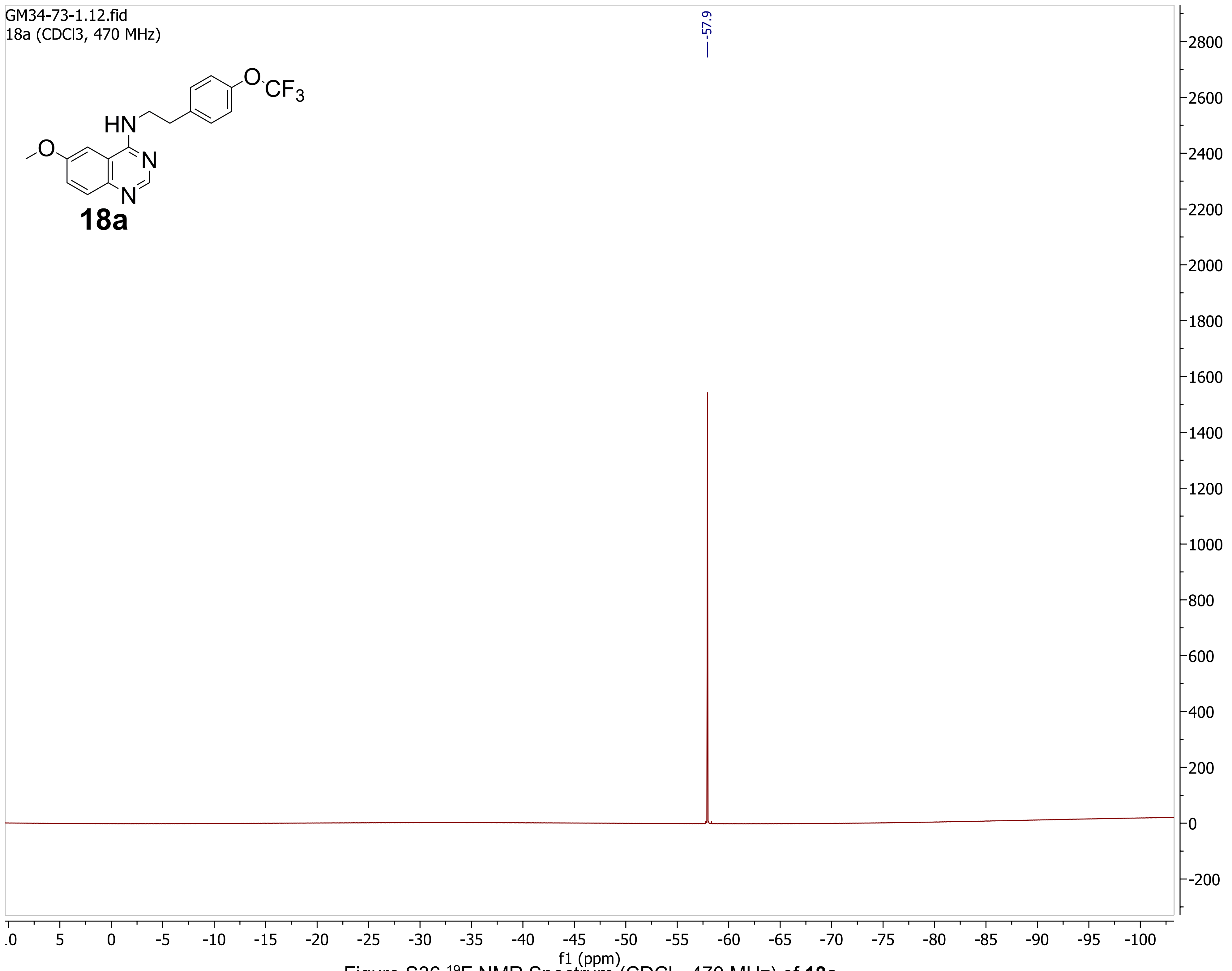

Figure S36 ^{19}F NMR Spectrum ($CDCl_3$, 470 MHz) of **18a**

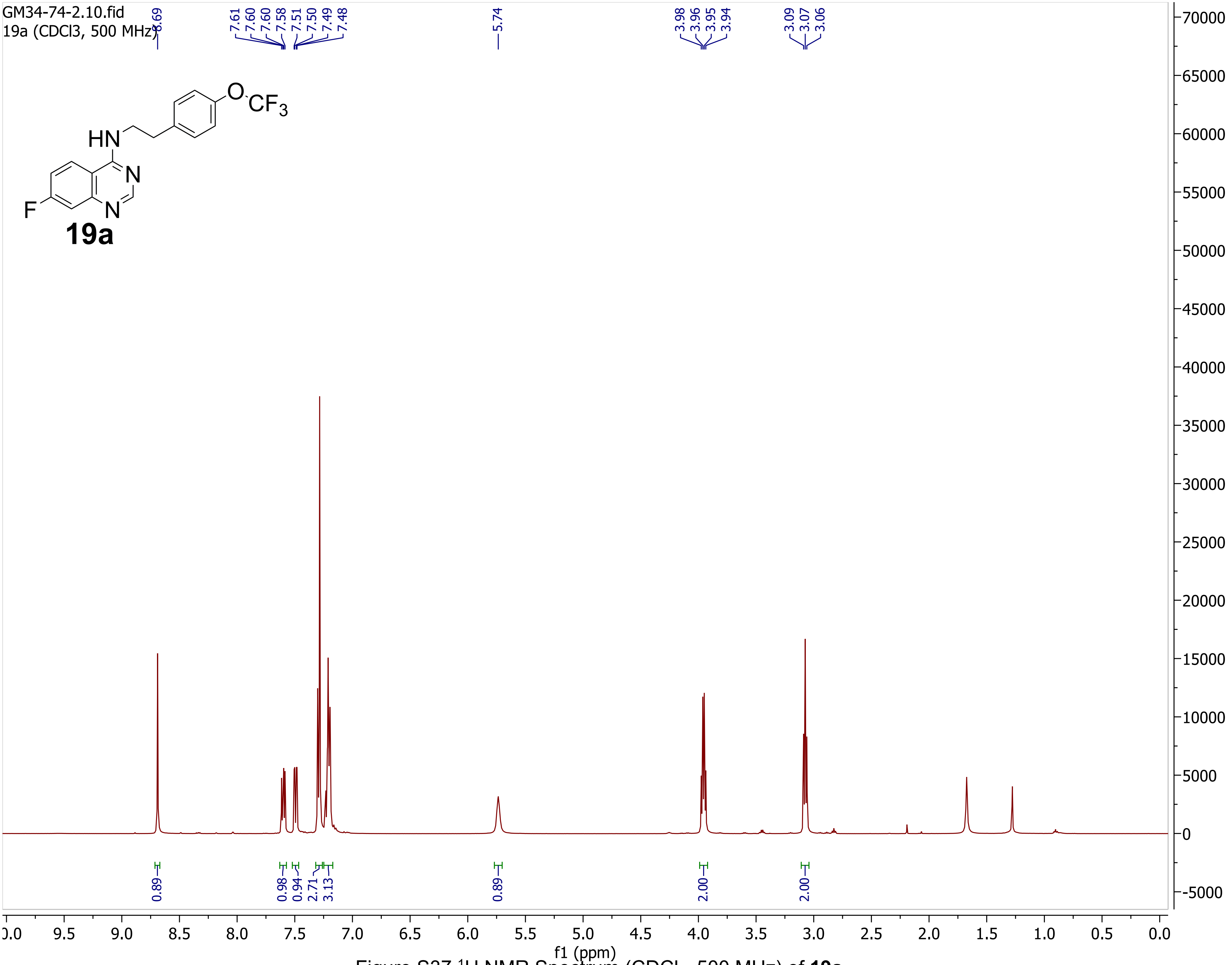

19a

Figure S37 ^{1}H NMR Spectrum ($CDCl_3$, 500 MHz) of **19a**

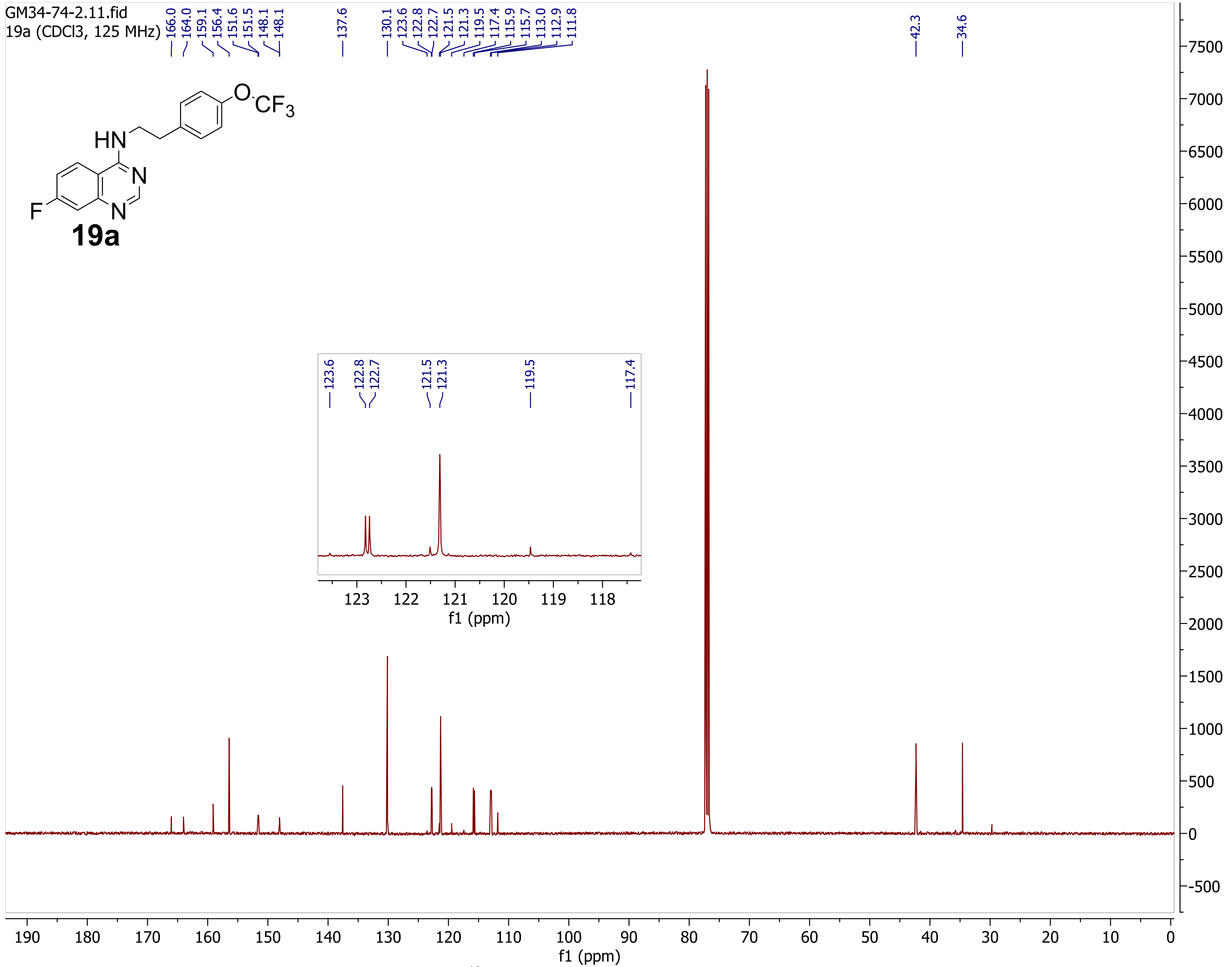

Figure S38 ^{13}C NMR Spectrum ($CDCl_3$, 125 MHz) of **19a**

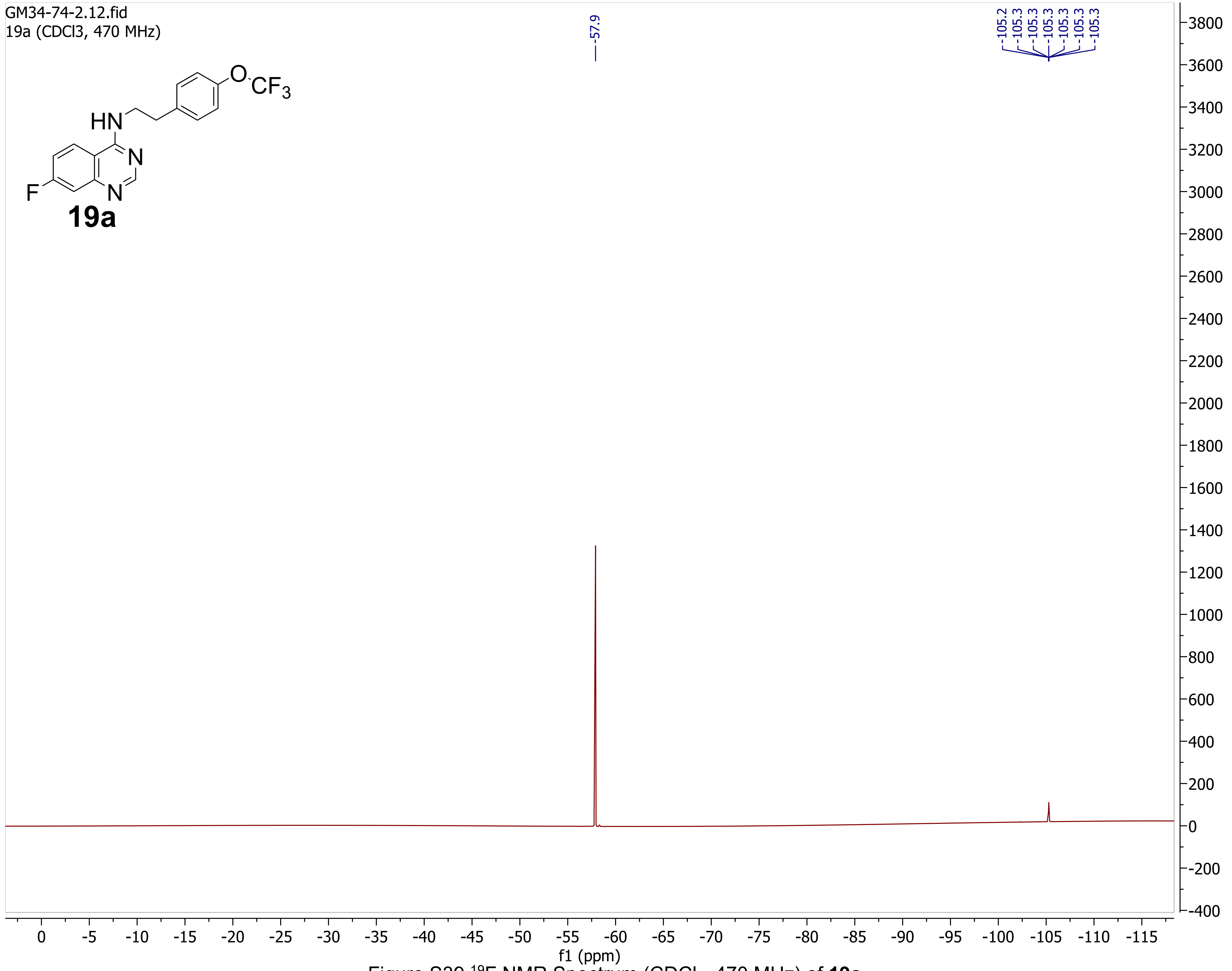

Figure S39 ^{19}F NMR Spectrum ($CDCl_3$, 470 MHz) of **19a**

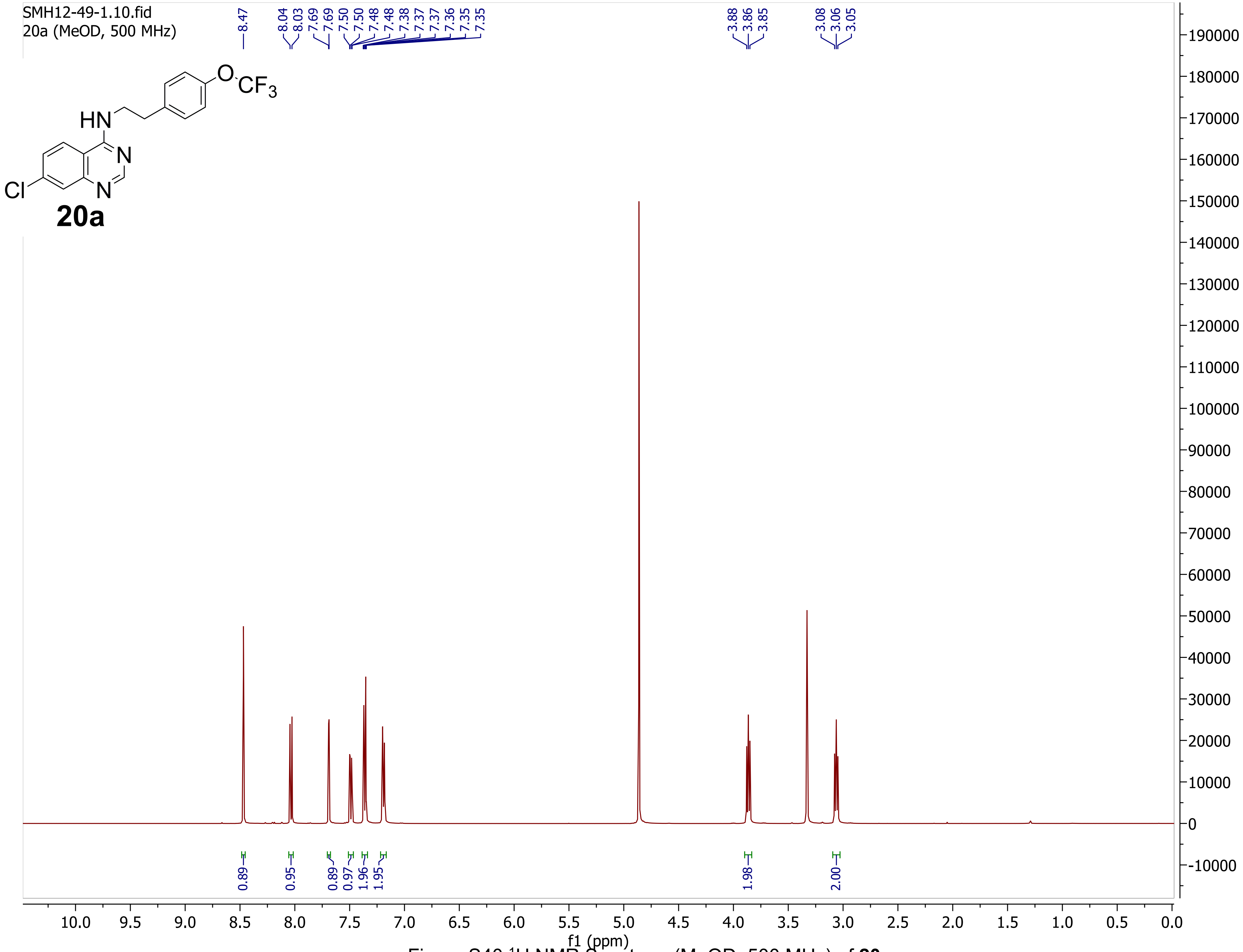

Figure S40 ¹H NMR Spectrum (MeOD, 500 MHz) of **20a**

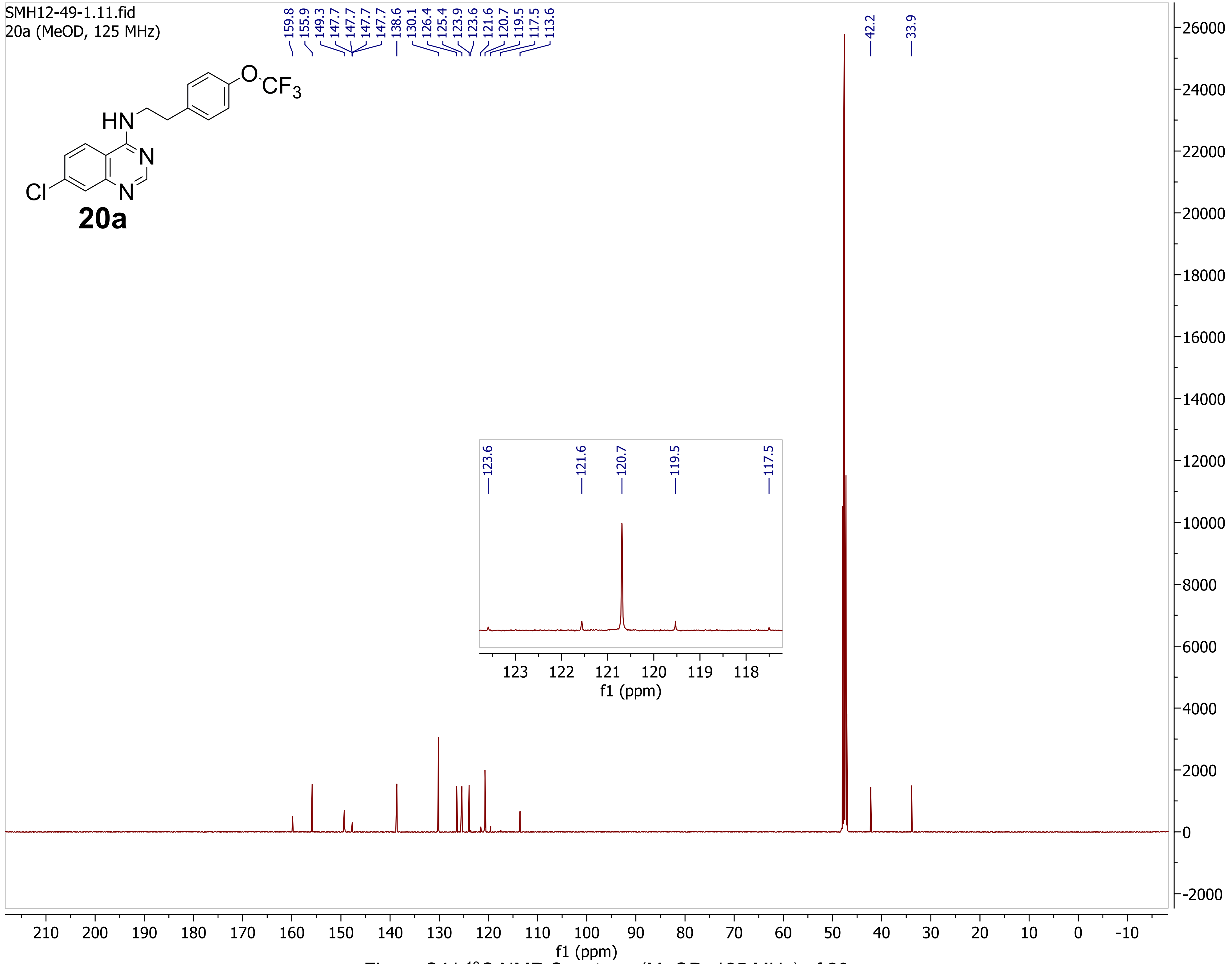

Figure S41 ^{13}C NMR Spectrum (MeOD, 125 MHz) of **20a**

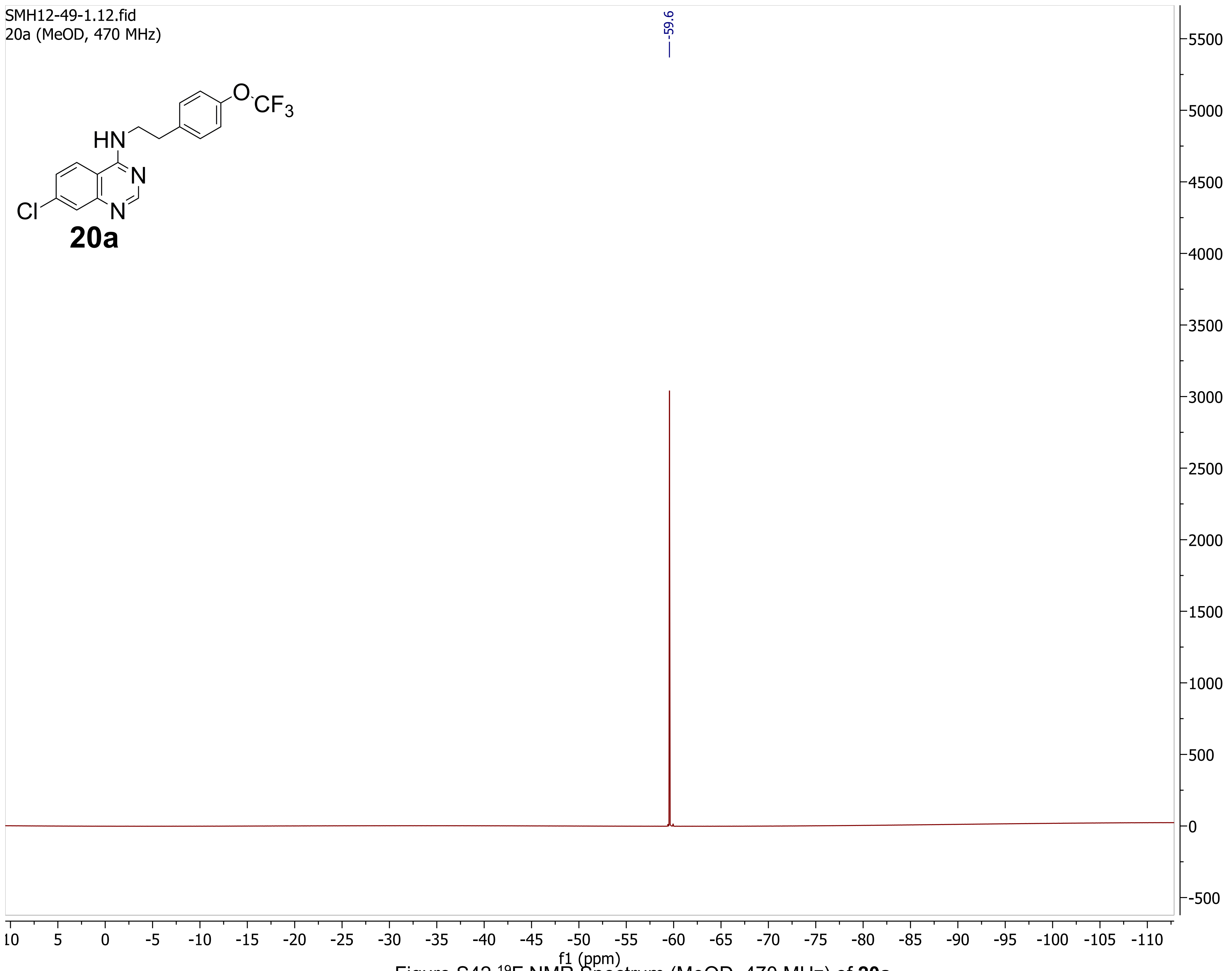

Figure S42 ^{19}F NMR Spectrum (MeOD, 470 MHz) of **20a**

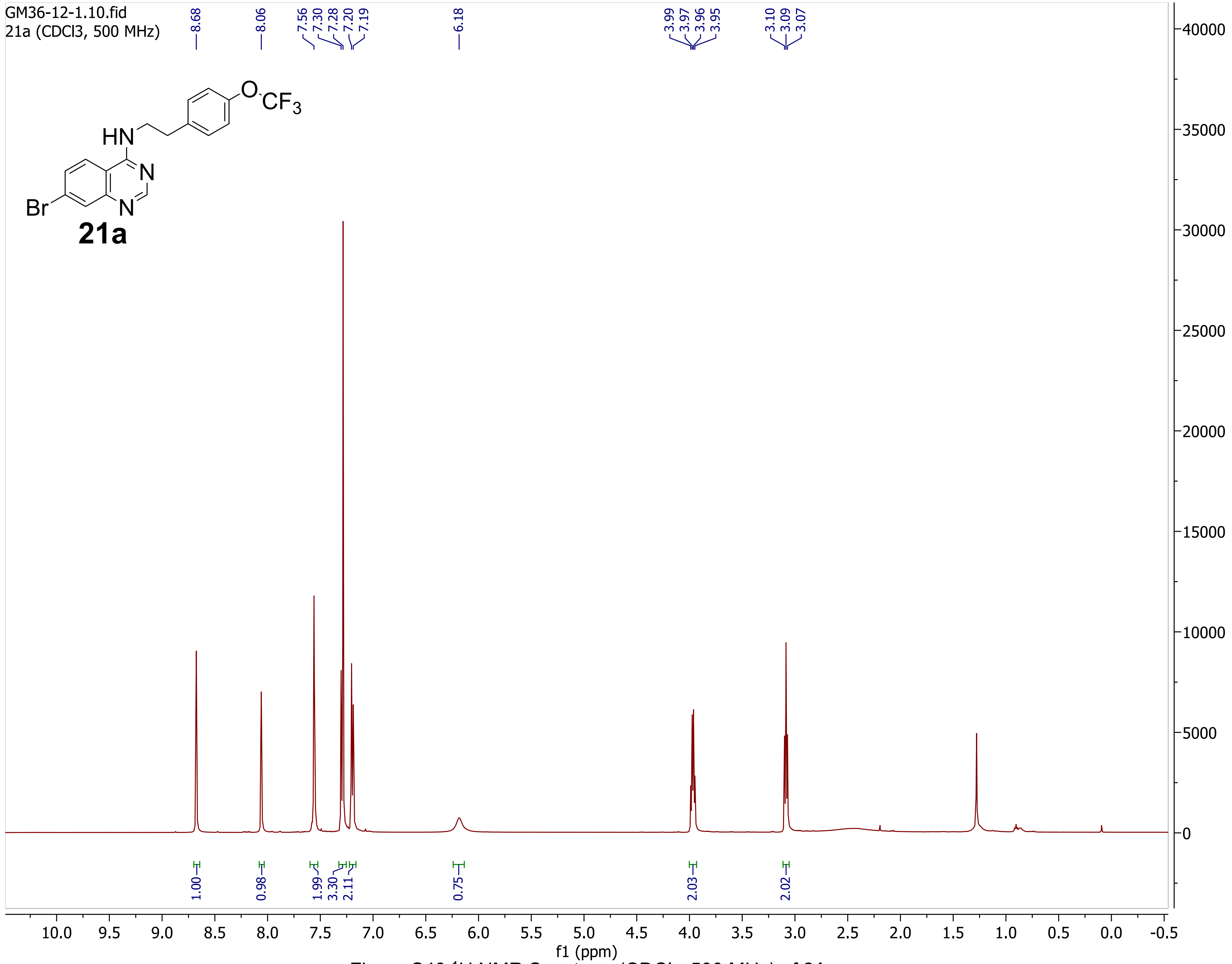

Figure S43 ^{1}H NMR Spectrum ($CDCl_3$, 500 MHz) of **21a**

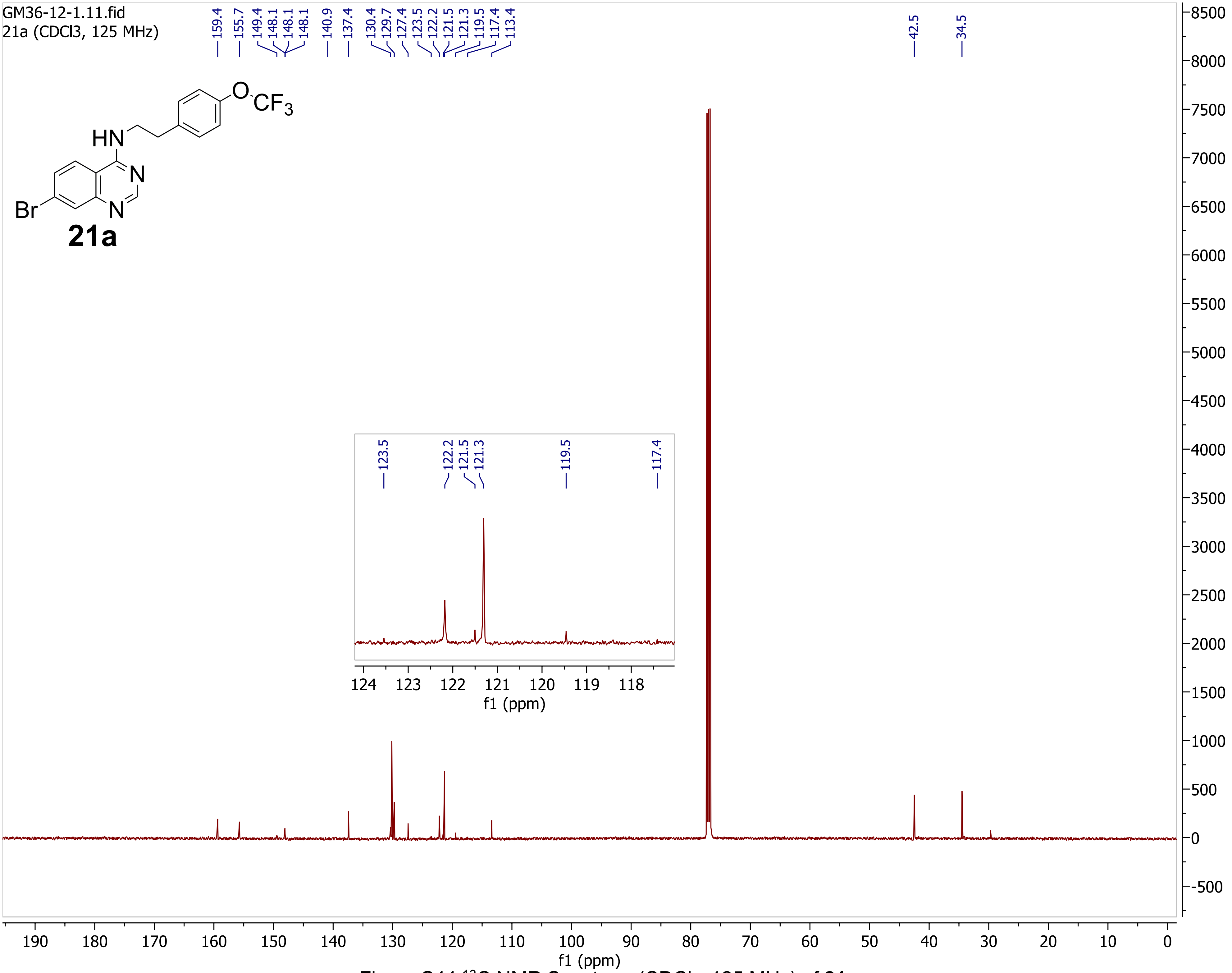

Figure S44 ^{13}C NMR Spectrum ($CDCl_3$, 125 MHz) of **21a**

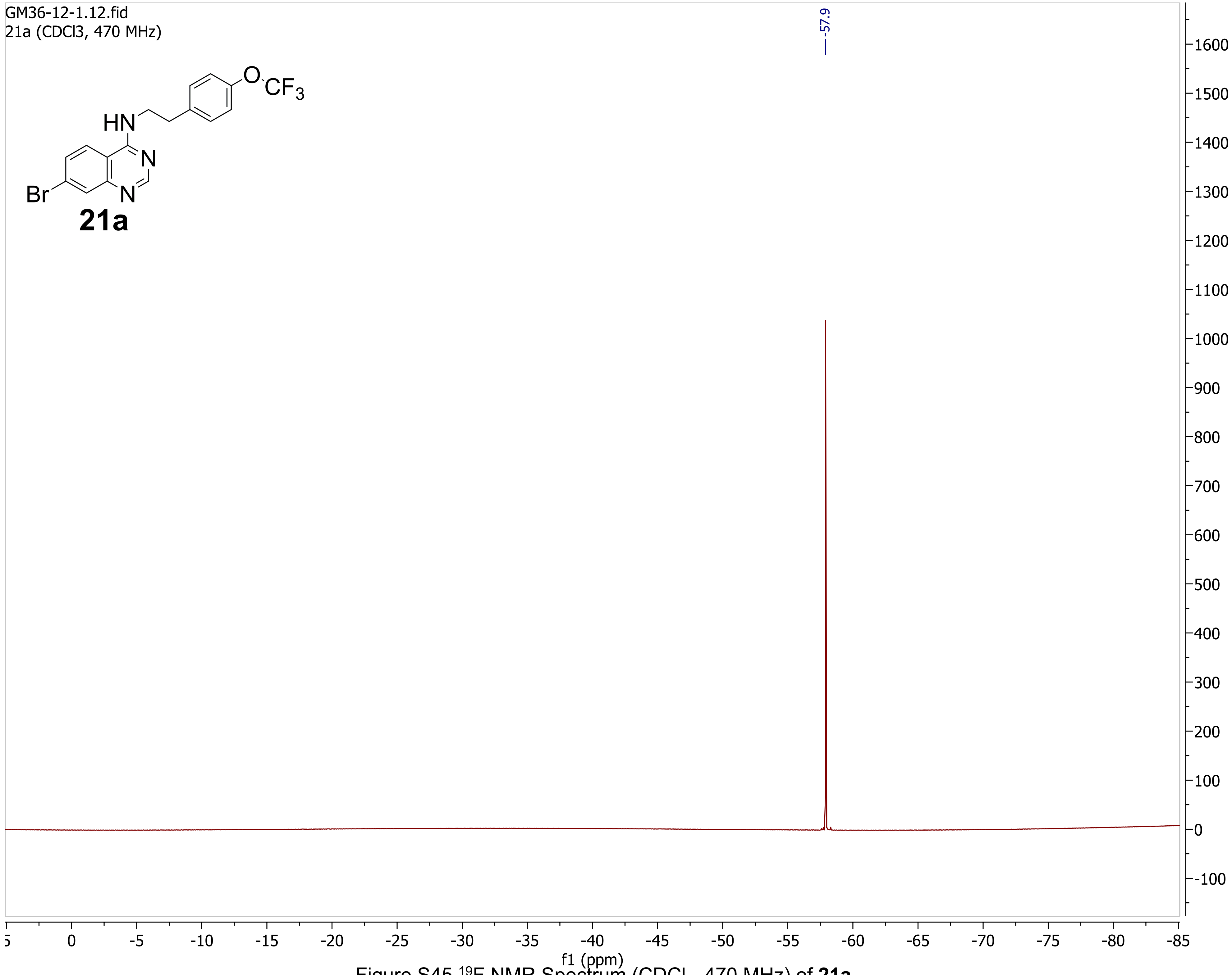

Figure S45 ^{19}F NMR Spectrum ($CDCl_3$, 470 MHz) of **21a**

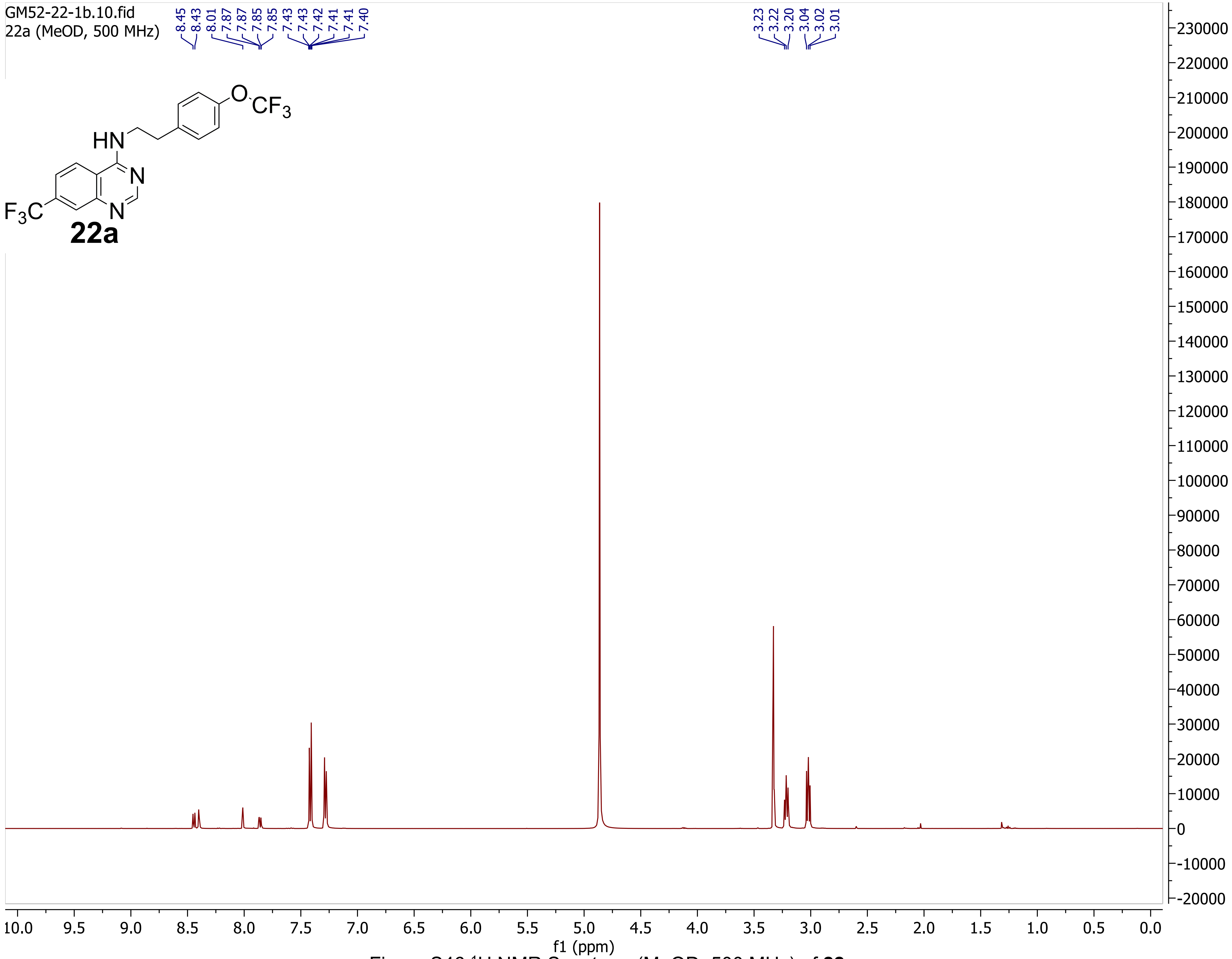

Figure S46 ^{1}H NMR Spectrum (MeOD, 500 MHz) of **22a**

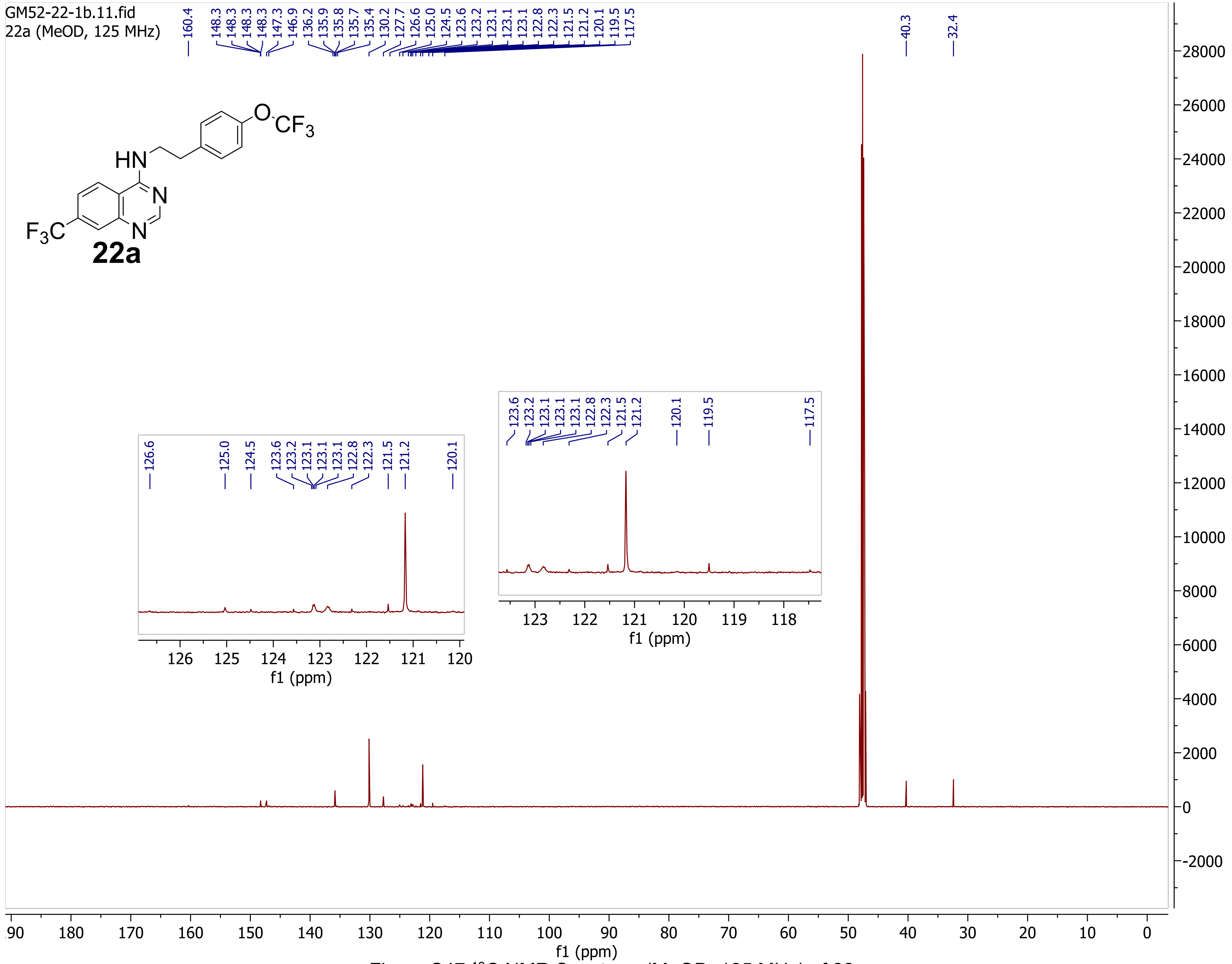

Figure S47 ^{13}C NMR Spectrum (MeOD, 125 MHz) of **22a**

GM52-22-1b.12.fid
22a (MeOD, 470 MHz)

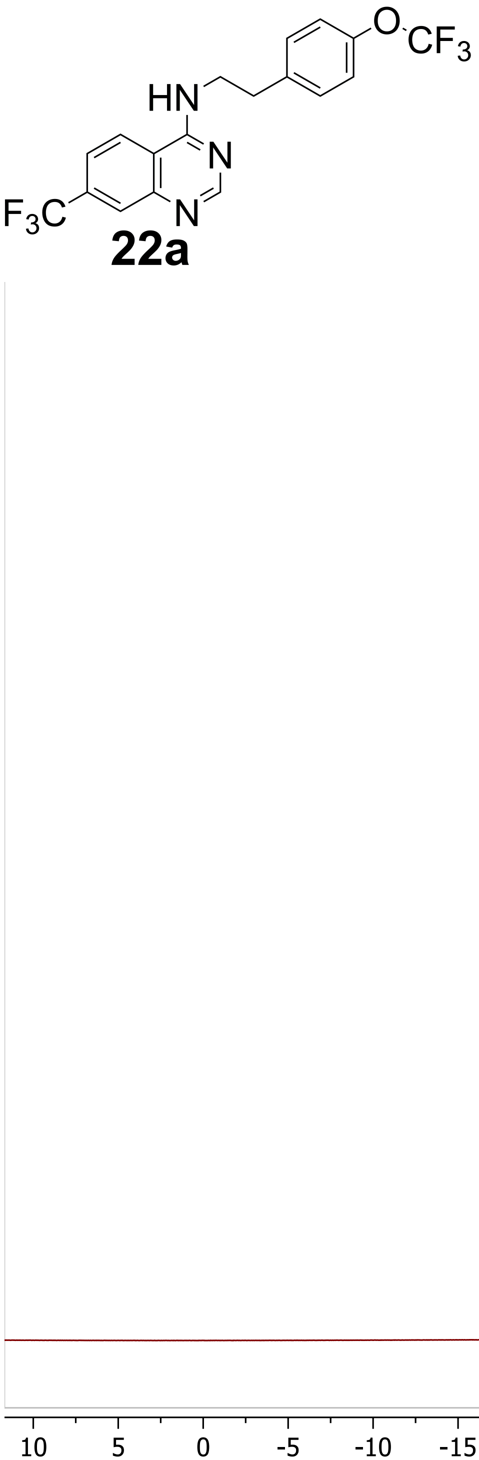
22a

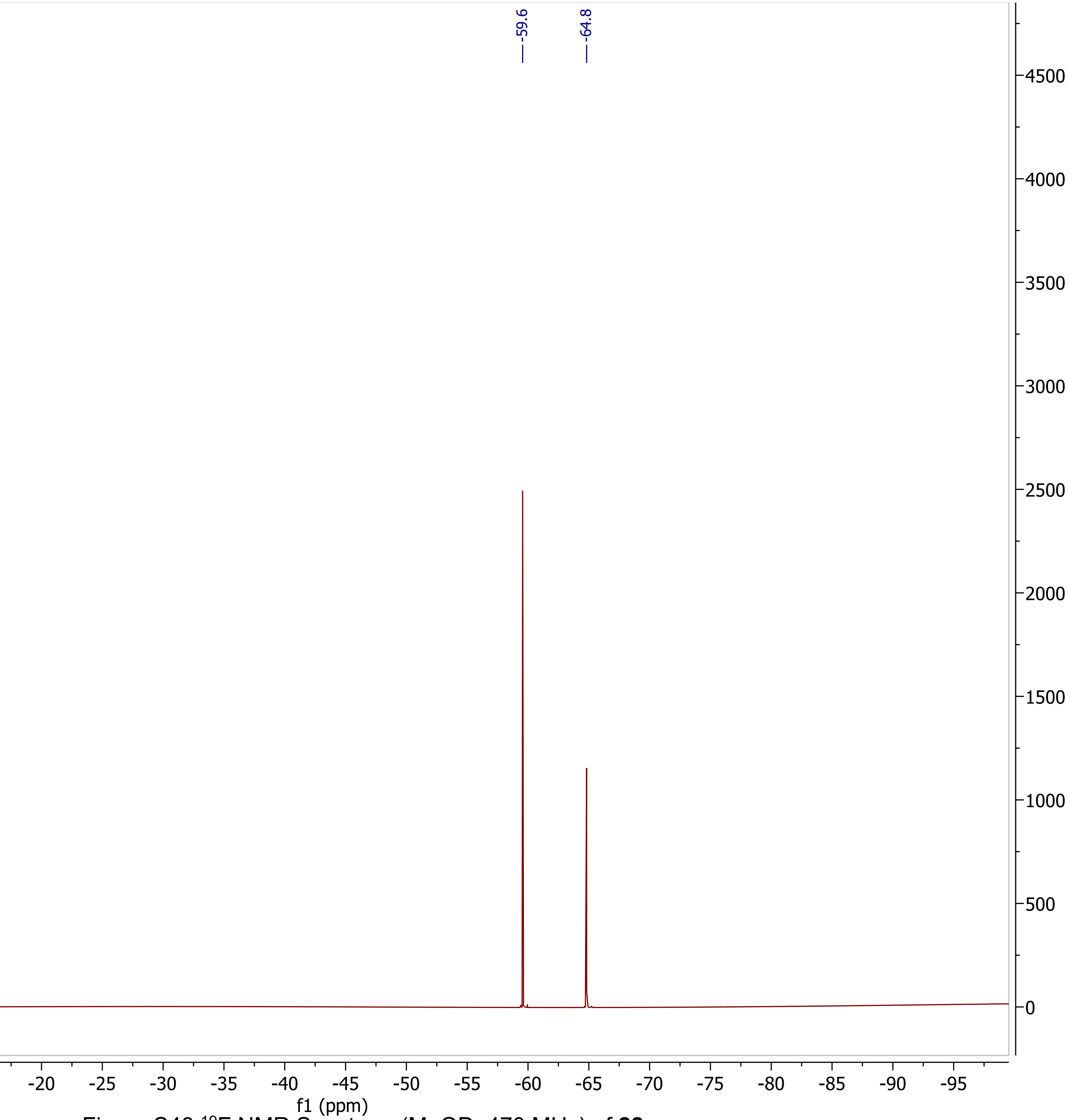

Figure S48 ^{19}F NMR Spectrum (MeOD, 470 MHz) of **22a**

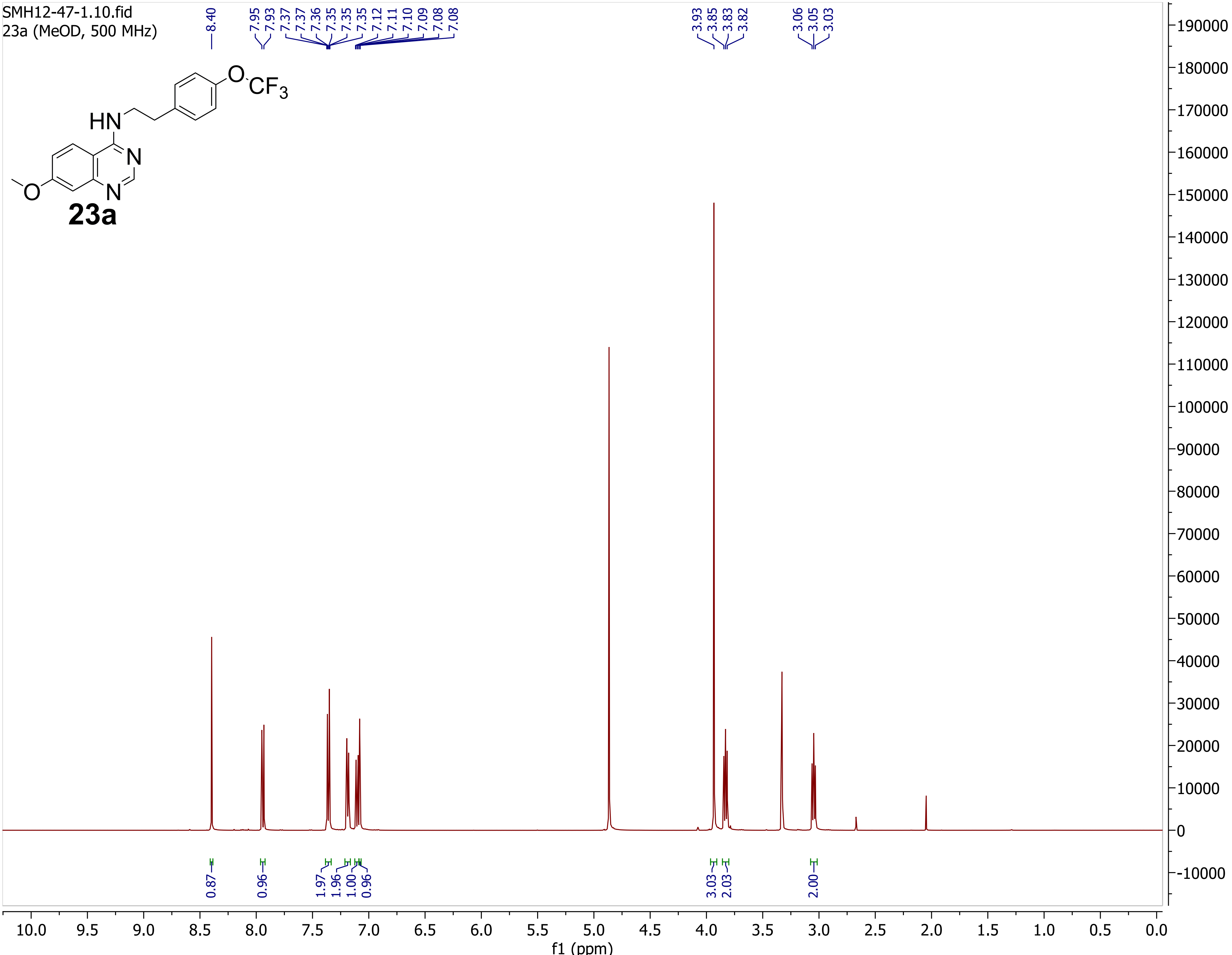

Figure S49 ^1H NMR Spectrum (MeOD, 500 MHz) of **23a**

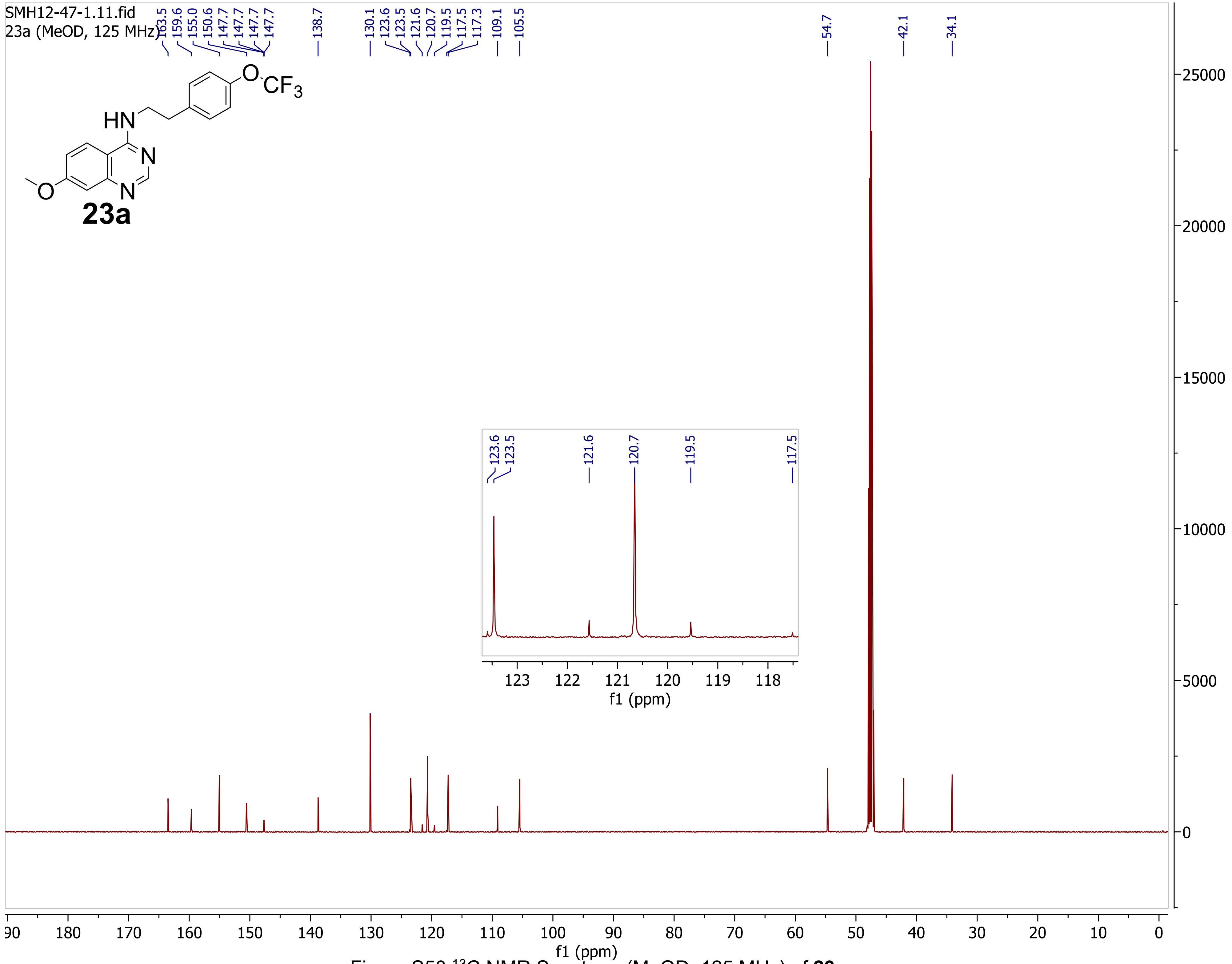

Figure S50 ^{13}C NMR Spectrum (MeOD, 125 MHz) of **23a**

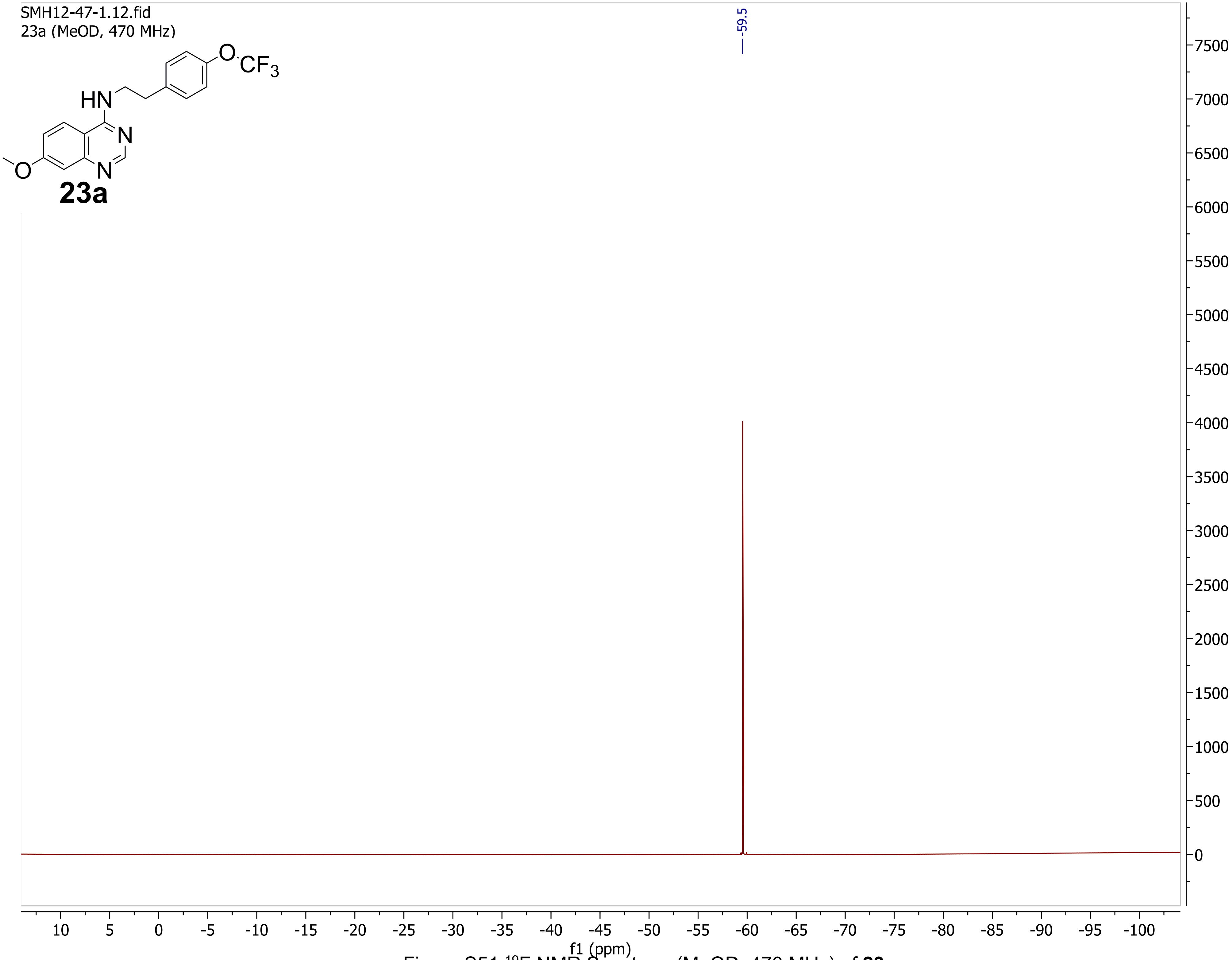

Figure S51 ^{19}F NMR Spectrum (MeOD, 470 MHz) of **23a**

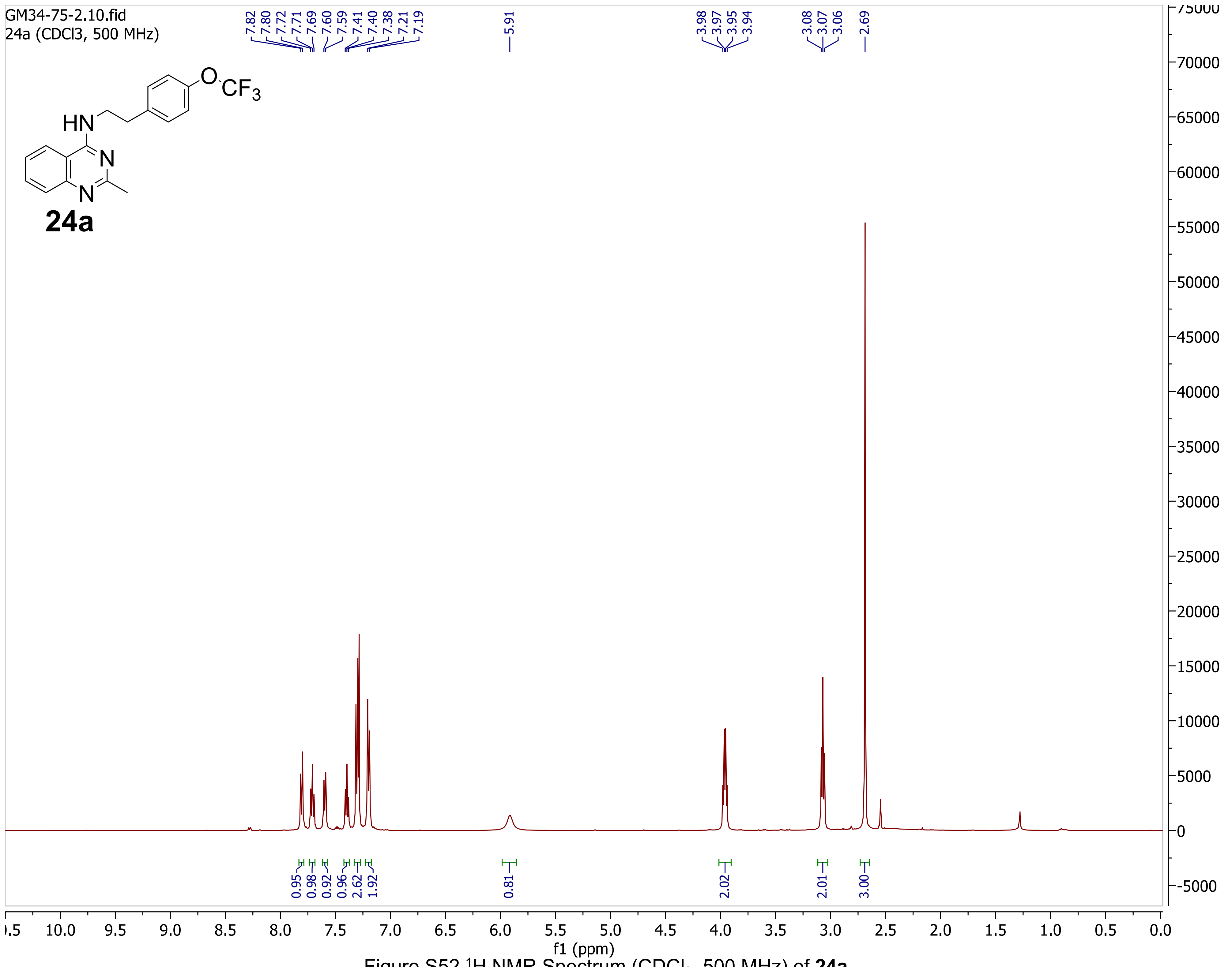

Figure S52 ^{1}H NMR Spectrum ($CDCl_3$, 500 MHz) of **24a**

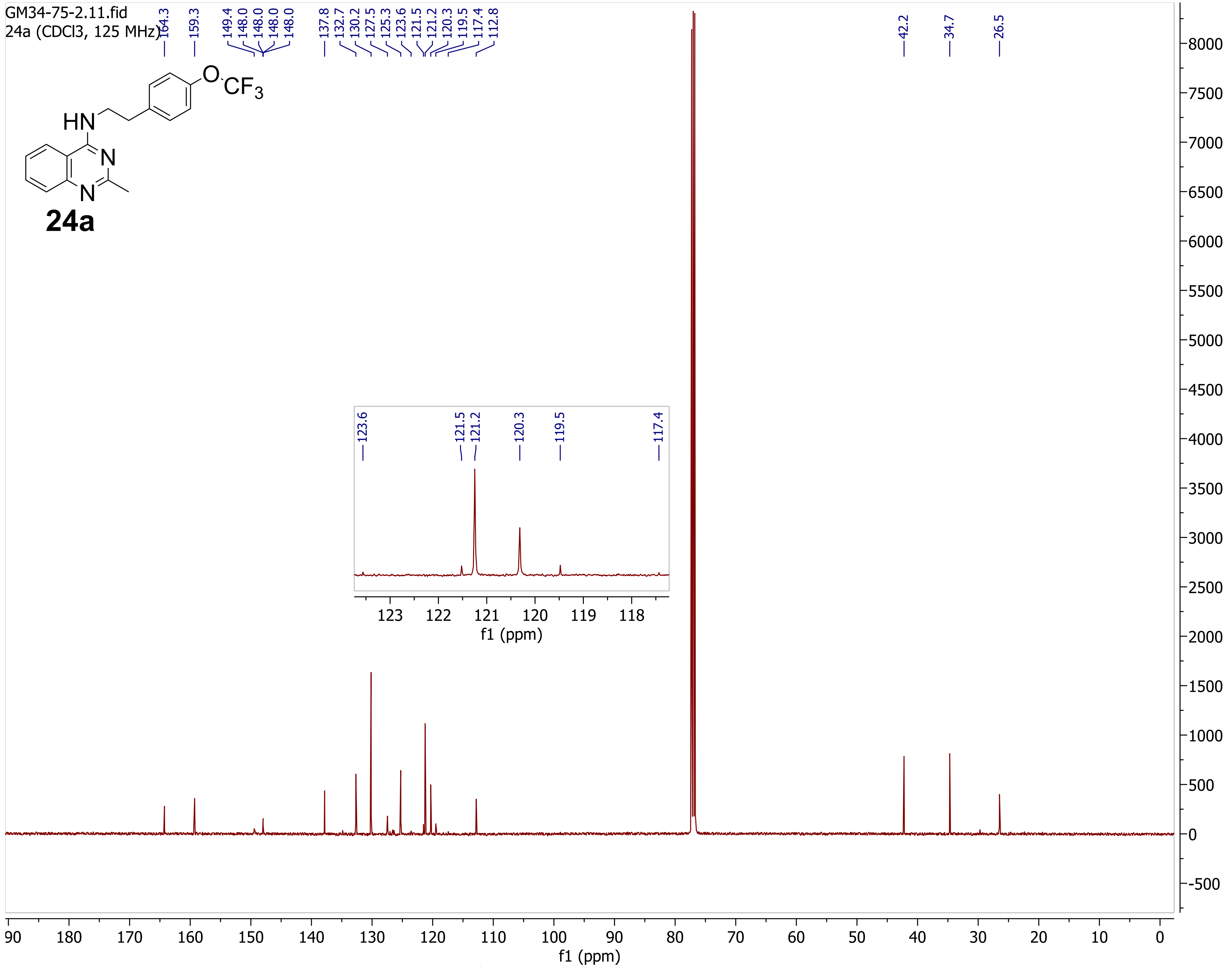

Figure S53 ^{13}C NMR Spectrum ($CDCl_3$, 125 MHz) of **24a**

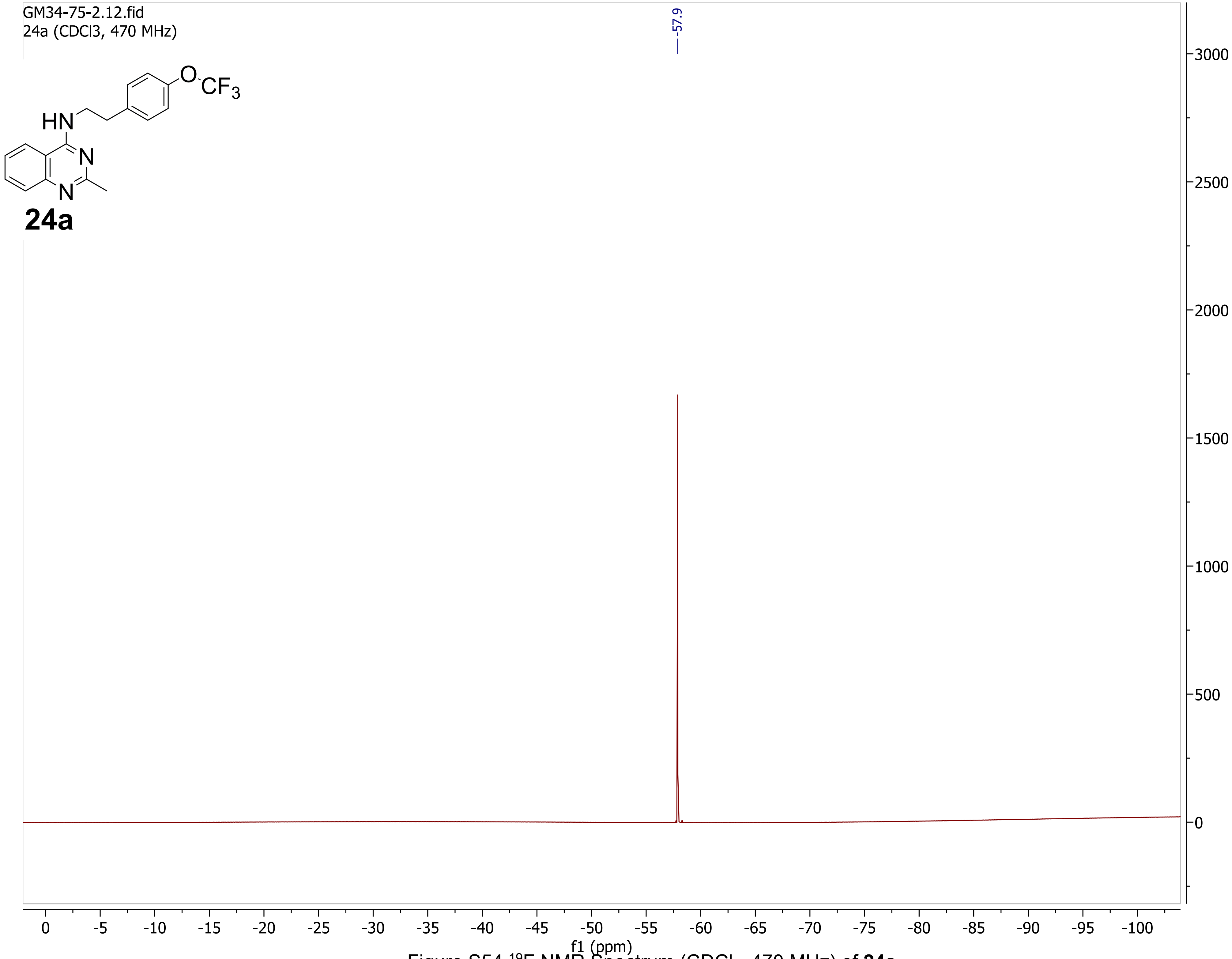

Figure S54 ^{19}F NMR Spectrum ($CDCl_3$, 470 MHz) of **24a**

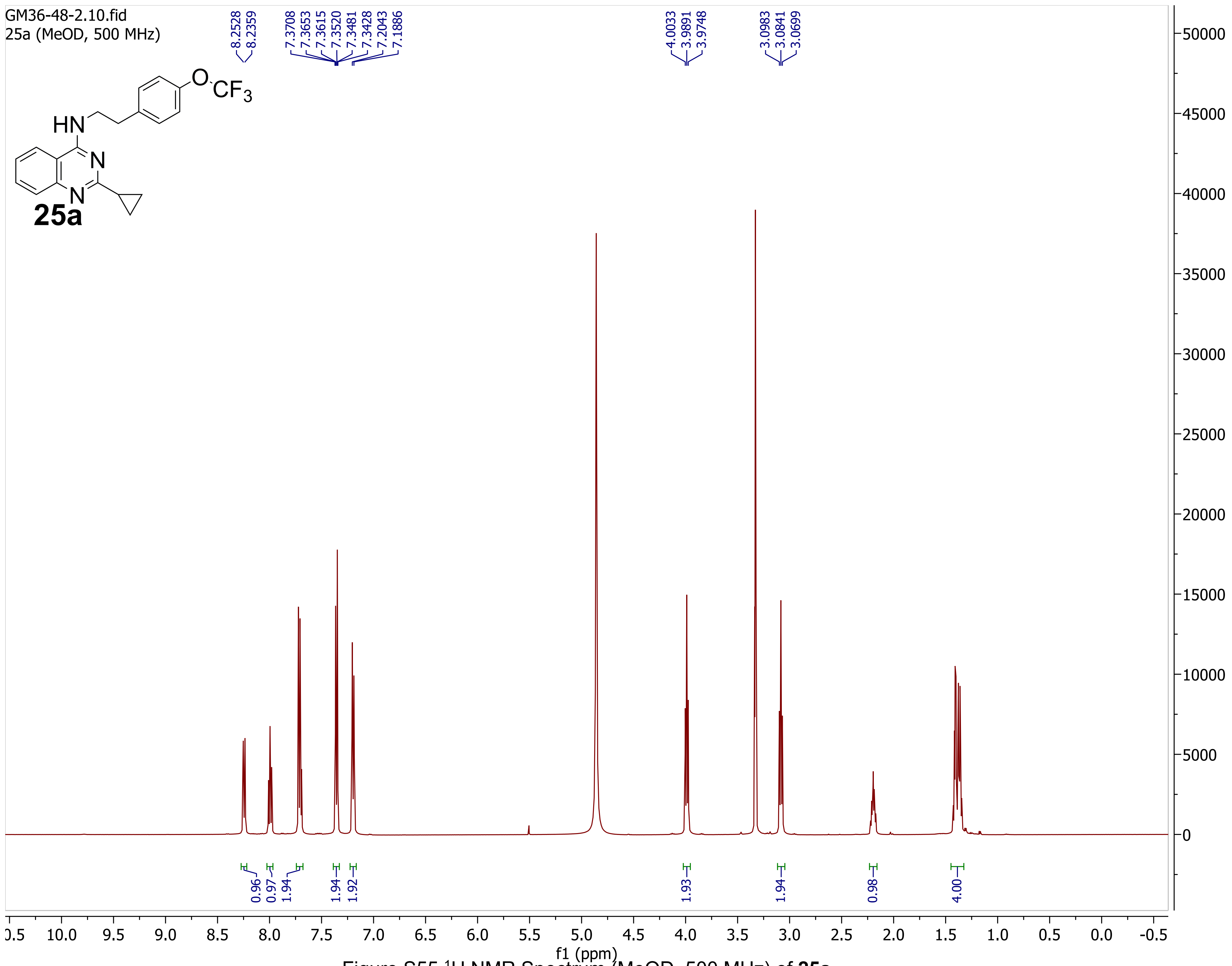

Figure S55 ^{1}H NMR Spectrum (MeOD, 500 MHz) of **25a**

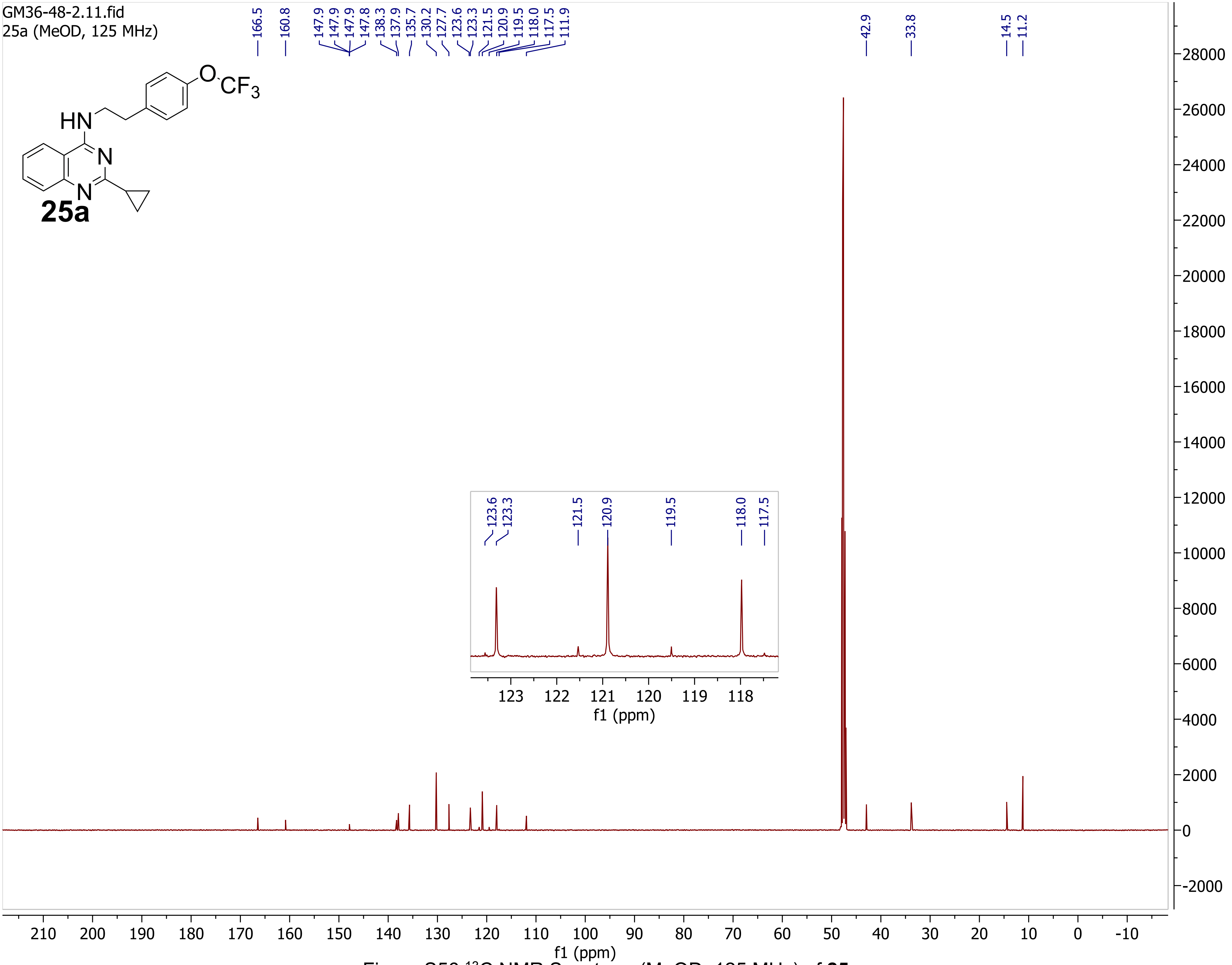

Figure S56 ^{13}C NMR Spectrum (MeOD, 125 MHz) of **25a**

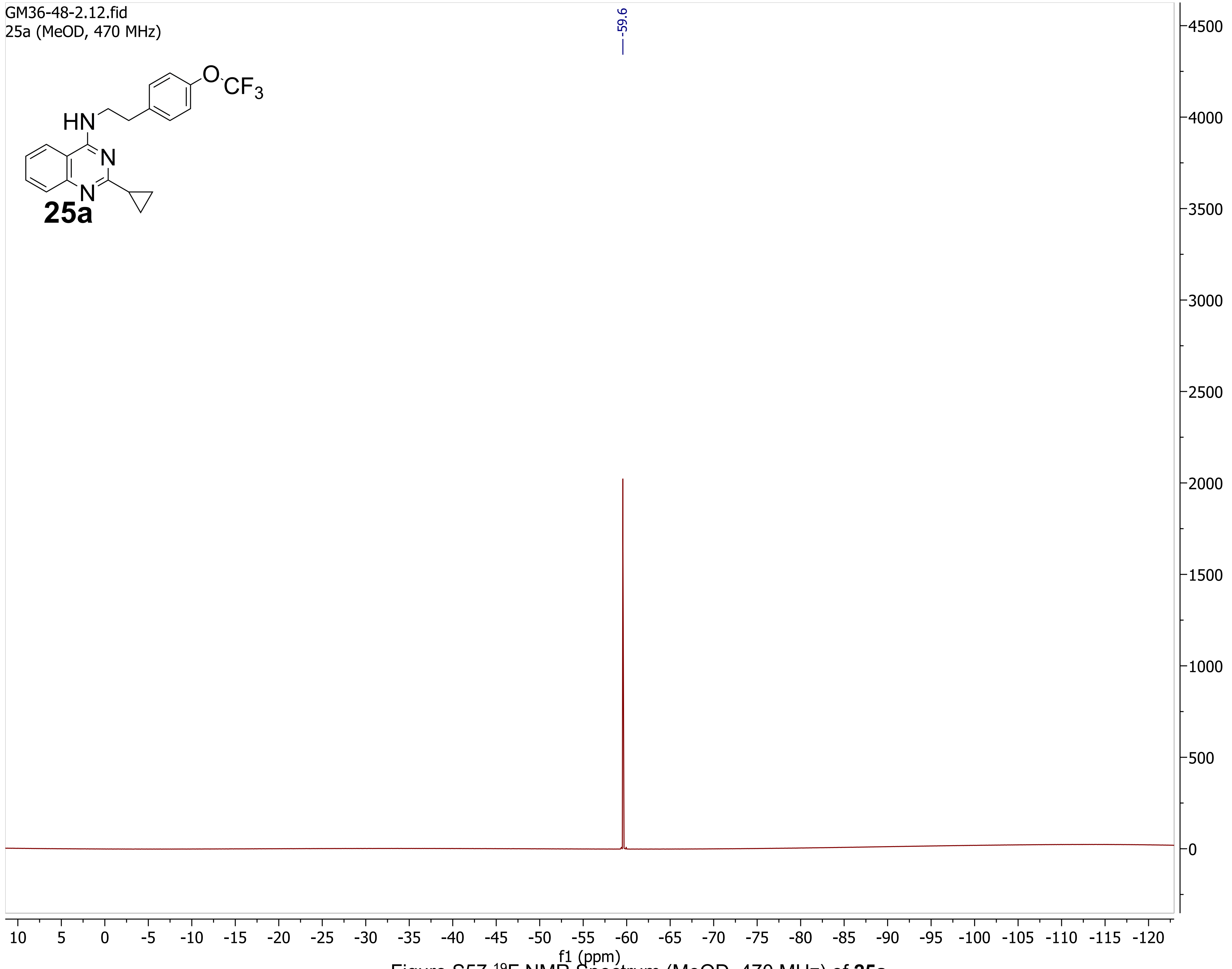

Figure S57 ^{19}F NMR Spectrum (MeOD, 470 MHz) of **25a**

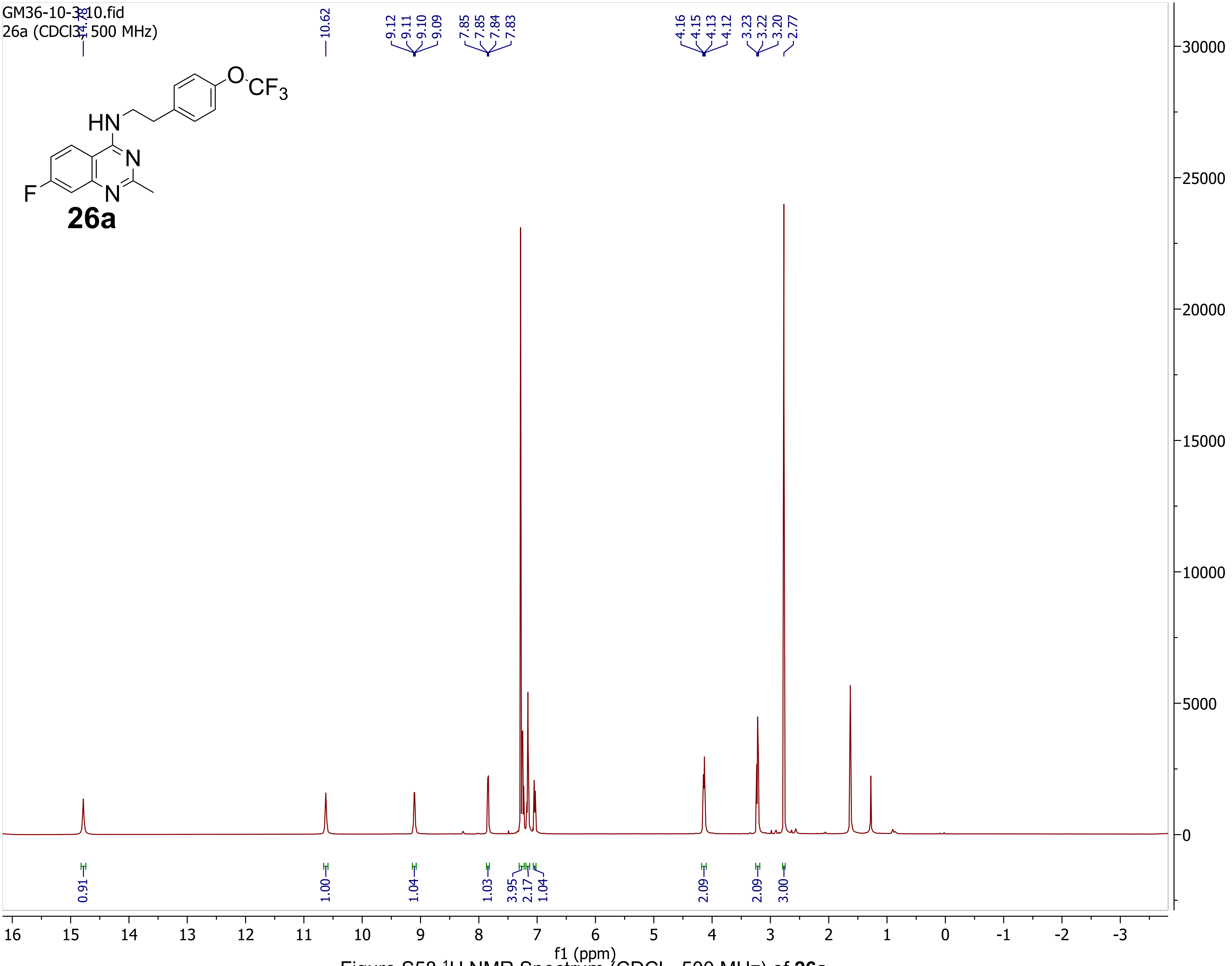

Figure S58 ^{1}H NMR Spectrum ($CDCl_3$, 500 MHz) of **26a**

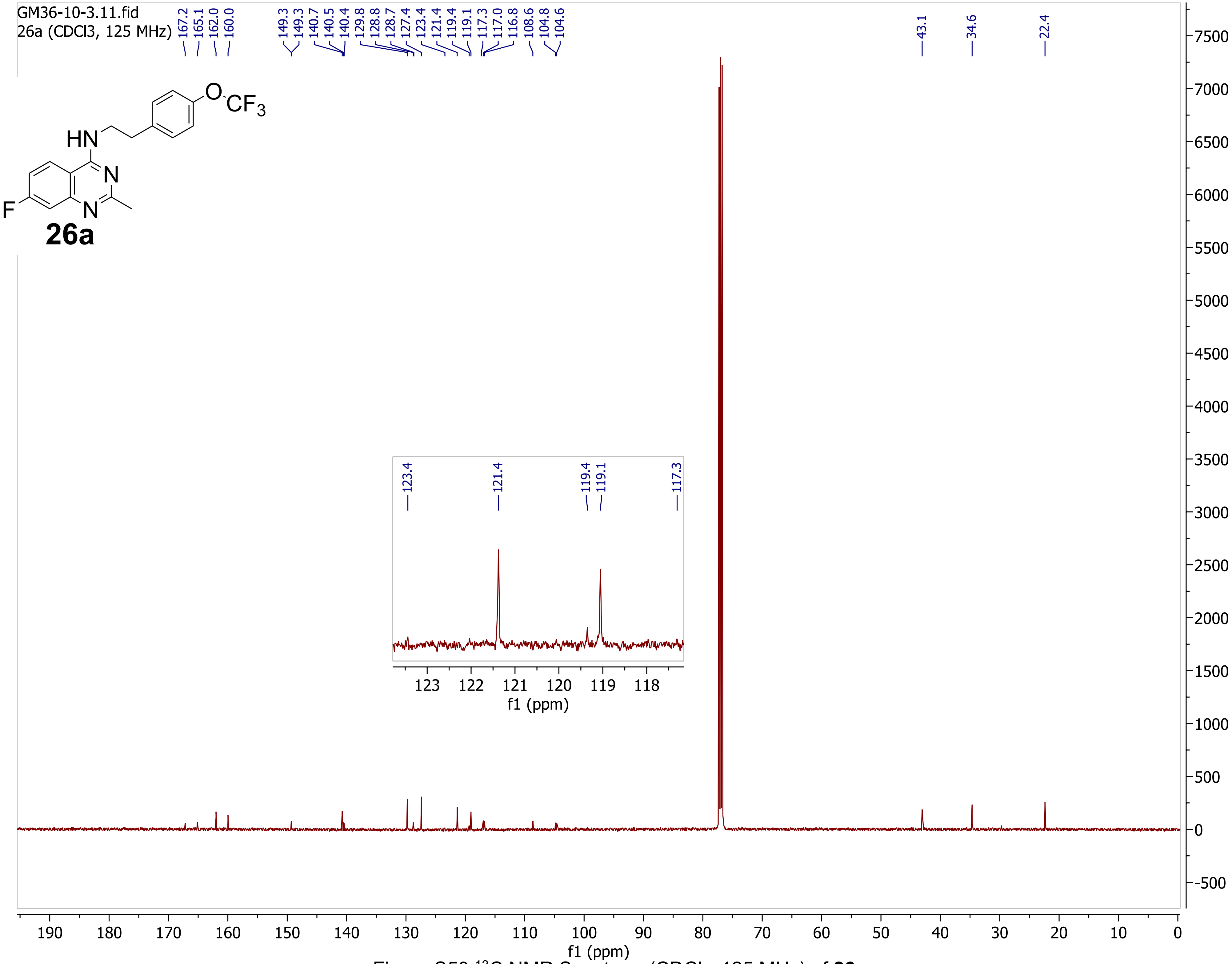

Figure S59 ^{13}C NMR Spectrum ($CDCl_3$, 125 MHz) of **26a**

GM36-10-3.12.fid
26a (CDCl3, 470 MHz)

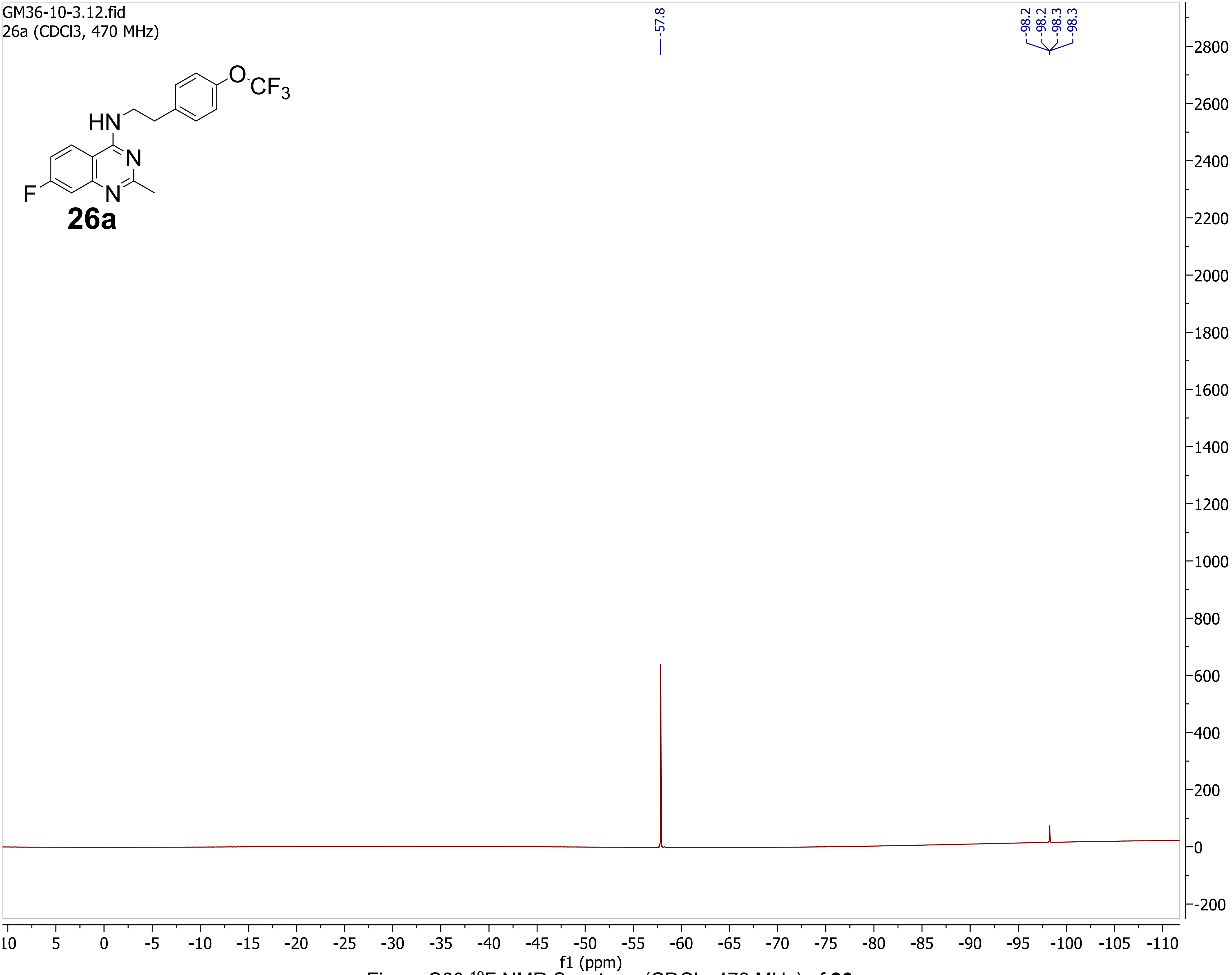

Figure S60 ^{19}F NMR Spectrum ($CDCl_3$, 470 MHz) of **26a**

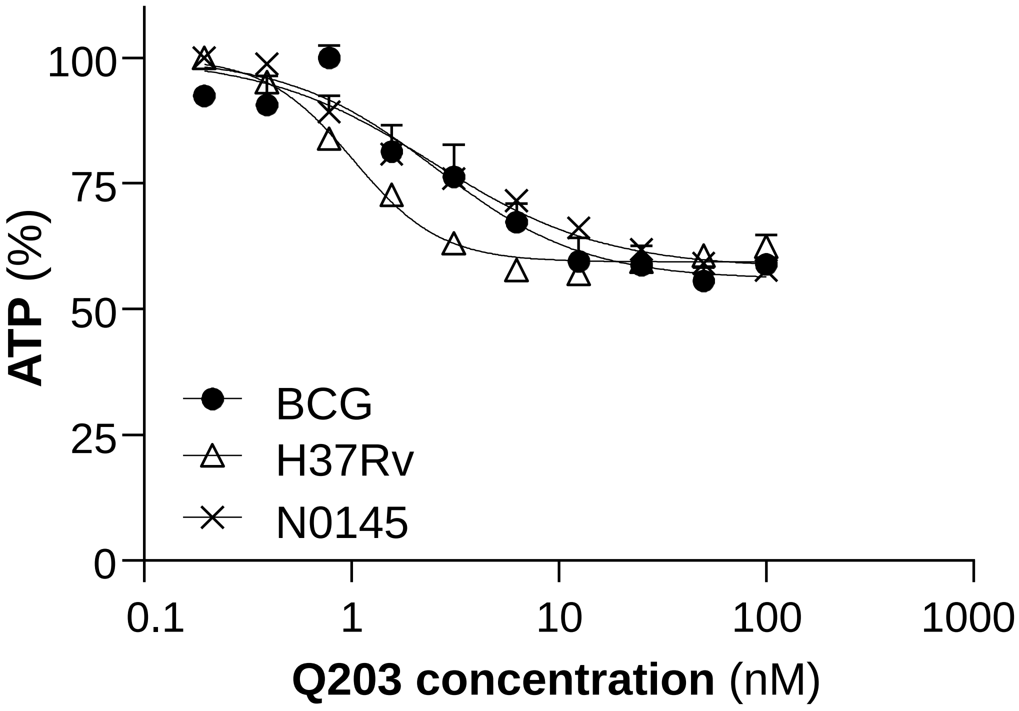

ATP dose response curves of Q203 in *M. bovis* BCG, *M. tuberculosis* H37Rv, and *M. tuberculosis* N0145. Q203 was tested in 10 points, two-fold serial dilution from a top concentration of 100 nM. ATP levels were measured after 15 hours of drug incubation. The ATP values were normalised to the untreated controls of each bacterial strain. Data are expressed as the mean ± S.D. for each condition of a representative experiment. Q203 ATP IC_{50} values were 2.6 nM for BCG, 1.0 nM for H37Rv, and 2.5 nM for N0145.

Figure S61 ATP dose response curves of Q203 in *M. bovis* BCG, *M. tuberculosis* H37Rv, and *M. tuberculosis* N0145
